# Zinc Status Alters Alzheimer's Disease Progression through NLRP3-Dependent Inflammation

**DOI:** 10.1523/JNEUROSCI.1980-20.2020

**Published:** 2021-03-31

**Authors:** Jack Rivers-Auty, Victor S. Tapia, Claire S. White, Michael J.D. Daniels, Samuel Drinkall, Paul T. Kennedy, Harry G. Spence, Shi Yu, Jack P. Green, Christopher Hoyle, James Cook, Amy Bradley, Alison E. Mather, Ruth Peters, Te-Chen Tzeng, Margaret J. Gordon, John H. Beattie, David Brough, Catherine B. Lawrence

**Affiliations:** ^1^Division of Neuroscience and Experimental Psychology, School of Biological Sciences, Faculty of Biology, Medicine and Health, Manchester Academic Health Science Centre, University of Manchester, Manchester M13 9PT, United Kingdom; ^2^Lydia Becker Institute of Immunology and Inflammation, University of Manchester, Manchester M13 9PT, United Kingdom; ^3^Tasmanian School of Medicine, College of Health and Medicine, University of Tasmania, Hobart, Tasmania 7000, Australia; ^4^Quadram Institute Bioscience, Norwich NR4 7UA, United Kingdom; ^5^University of East Anglia, Norwich NR4 7TJ, United Kingdom; ^6^School of Psychology, University of New South Wales, Sydney, New South Wales 2052, Australia; ^7^Neuroscience Research Australia, Sydney, New South Wales 2031, Australia; ^8^Immunology and Inflammation, Bristol-Myers Squibb (Celgene Corporation), Cambridge, Massachusetts 02140; ^9^Rowett Institute of Nutrition and Health, University of Aberdeen, Aberdeen AB25 2ZD, United Kingdom

**Keywords:** Alzheimer's disease, APP/PS1, inflammation, microglia, NLRP3, zinc

## Abstract

Alzheimer's disease is a devastating neurodegenerative disease with a dramatically increasing prevalence and no disease-modifying treatment. Inflammatory lifestyle factors increase the risk of developing Alzheimer's disease. Zinc deficiency is the most prevalent malnutrition in the world and may be a risk factor for Alzheimer's disease potentially through enhanced inflammation, although evidence for this is limited. Here we provide epidemiological evidence suggesting that zinc supplementation was associated with reduced risk and slower cognitive decline, in people with Alzheimer's disease and mild cognitive impairment. Using the APP/PS1 mouse model of Alzheimer's disease fed a control (35 mg/kg zinc) or diet deficient in zinc (3 mg/kg zinc), we determined that zinc deficiency accelerated Alzheimer's-like memory deficits without modifying amyloid β plaque burden in the brains of male mice. The NLRP3-inflammasome complex is one of the most important regulators of inflammation, and we show here that zinc deficiency in immune cells, including microglia, potentiated NLRP3 responses to inflammatory stimuli *in vitro*, including amyloid oligomers, while zinc supplementation inhibited NLRP3 activation. *APP/PS1* mice deficient in NLRP3 were protected against the accelerated cognitive decline with zinc deficiency. Collectively, this research suggests that zinc status is linked to inflammatory reactivity and may be modified in people to reduce the risk and slow the progression of Alzheimer's disease.

**SIGNIFICANCE STATEMENT** Alzheimer's disease is a common condition mostly affecting the elderly. Zinc deficiency is also a global problem, especially in the elderly and also in people with Alzheimer's disease. Zinc deficiency contributes to many clinical disorders, including immune dysfunction. Inflammation is known to contribute to the risk and progression of Alzheimer's disease; thus, we hypothesized that zinc status would affect Alzheimer's disease progression. Here we show that zinc supplementation reduced the prevalence and symptomatic decline in people with Alzheimer's disease. In an animal model of Alzheimer's disease, zinc deficiency worsened cognitive decline because of an enhancement in NLRP3-driven inflammation. Overall, our data suggest that zinc status affects Alzheimer's disease progression, and that zinc supplementation could slow the rate of cognitive decline.

## Introduction

The global prevalence of dementia (of which the most common type is Alzheimer's disease) is growing, with 152 million people predicted to be living with dementia by 2050 (from 50 million in 2018), and there are no effective treatments (Alzheimer's Association, 2015). Approximately 92% of people living with dementia also suffer from one or more long-term diseases (comorbidities) (Browne et al., 2017). It has been established that disease-associated inflammation increases the risk of developing dementia, and includes inflammation associated with several comorbidities for Alzheimer's disease, such as infection, diabetes, atherosclerosis, and obesity (Whitmer et al., 2005; Profenno et al., 2010). Zinc is an essential trace element obtained from the diet that regulates the expression and activation of many biological molecules. Maintenance of adequate zinc levels through a balanced diet is therefore important to health. Zinc deficiency affects up to 2 billion people worldwide and has profound effects on immune system function (Prasad, 2012). Given its prevalence, therefore, it is likely that zinc deficiency represents a significant comorbidity for Alzheimer's disease that could contribute to poorer quality of life and disease outcome. Previous associations between zinc deficiency and Alzheimer's disease include several studies reporting reduced plasma zinc levels in people with Alzheimer's disease (Ventriglia et al., 2015). Overall, these data suggest that people with Alzheimer's disease are at risk of zinc deficiency, although the potential effect zinc deficiency is having on the condition is currently debated.

Zinc is a critical micronutrient that plays an essential role in a vast number of physiological processes; ∼10% of proteins have zinc binding domains; zinc also plays a role in membrane stabilization and can act directly as a signaling molecule (Sensi et al., 2009; Kambe et al., 2015). Because of its physiological importance, its cytosolic and extracellular concentrations are tightly controlled by several mechanisms, including two main families of transporters, the zinc transporters and the Zrt-Irt-related proteins, as well as zinc storage proteins (e.g., metallothioneins) and vesical storage of free zinc (Sensi et al., 2009; Kambe et al., 2015). Typically, Zrt-Irt-related protein transporters act to increase cytosolic concentrations of zinc, whereas zinc transporters act to decrease cytosolic concentrations by movement of zinc out of the cell or into cellular compartments, including vesicles, Golgi, and endosomes (Sensi et al., 2009; Kambe et al., 2015). The ubiquity and diversity of molecular functions that involve zinc have made researching the interactions between zinc status and Alzheimer's disease difficult.

Several potential pathophysiological links of Alzheimer's disease and zinc status have been investigated. The role zinc binding has in amyloid fibril formation has been a substantial area of interest. Several *in vitro* and *in vivo* studies report that excessive zinc promotes the formation of amyloid oligomers and fibrils through direct zinc-amyloid interactions. Amyloid oligomers and fibrils are one of the pathologic hallmarks of Alzheimer's disease and considered a likely driver of the disease (Esler et al., 1996; Brown et al., 1997; Lee et al., 2018). These findings have driven preliminary Alzheimer's clinical trials on compounds, such as PBT2, which increase cellular uptake of zinc, thereby reducing extracellular zinc levels. These trials have had largely positive results (Faux et al., 2010).

Another focus of the Alzheimer's disease zinc field is the role of zinc proteases in beneficial and pathologic processes. Zinc is an essential cofactor in a range of proteases, including proteases that cleave the amyloid precursor protein into nonpathogenic peptides, including the a-disintegrin and metalloproteinases (Gough et al., 2011). Zinc is also a cofactor in proteases that digest the pathologic forms of amyloid into nonoligomerizing products, including the insulin-degrading enzyme (Gough et al., 2011). Finally, the zinc protease matrix metallopeptidase 9 plays a critical role in the healthy functioning of CNS, particularly the activation of BDNF (Yang et al., 2014; Frazzini et al., 2018). BDNF is an important neurotrophin that plays a role in neuronal plasticity and synapse formation. Previously, the administration of zinc chelators in mice has been shown to lower active BDNF levels impairing cognitive performance (Yang et al., 2014; Frazzini et al., 2018). While zinc and Alzheimer's disease has been an active field of research, little attention has been given to the known effects of zinc status on immune function, particularly neuroinflammation, an established driver of Alzheimer's disease pathology.

We previously reported that zinc depletion of macrophages activates an inflammatory complex called the NLRP3 (NACHT, LRR, and PYD domains-containing protein 3) inflammasome driving release of the pro-inflammatory cytokine interleukin-1β (IL-1β) from the cell (Summersgill et al., 2014). The NLRP3 inflammasome is formed in macrophages, and microglia in the brain, in response to pathogenic or damage-associated stress and drives an inflammatory response. The NLRP3 inflammasome complex is composed of NLRP3, the adaptor protein ASC (apoptosis-associated speck-like protein containing a caspase activation and recruitment domain), and the protease caspase-1, which is responsible for cleaving the inactive precursor pro-IL-1β to an active secreted IL-1β molecule (Brough et al., 2017; Heneka et al., 2018). The NLRP3 inflammasome is suggested to contribute to the damaging inflammation that worsens Alzheimer's disease (Heneka et al., 2013, 2018; Daniels et al., 2016; Dempsey et al., 2017; White et al., 2017). NLRP3 is also suggested to contribute to age-related cognitive decline (Youm et al., 2013).

This study aimed to test the hypothesis that zinc status can influence the development of Alzheimer's disease. We report here that zinc supplementation in people reduced the prevalence and symptomatic decline of Alzheimer's disease, and that zinc deficiency in an Alzheimer's disease mouse model accelerated cognitive decline through potentiation of NLRP3-dependent inflammation, an effect that was reversible. These data suggest that zinc deficiency is a treatable comorbidity, which, if managed correctly, potentially through the use of zinc supplementation, could slow the rate of cognitive decline in people with Alzheimer's disease.

## Materials and Methods

### 

#### Epidemiological study

##### Data acquisition

Data used in the preparation of this article were obtained from the Alzheimer's Disease Neuroimaging Initiative (ADNI) database (http://adni.loni.usc.edu). The ADNI was launched in 2003 as a public-private partnership, led by principal investigator Dr. Michael W. Weiner. The primary goal of ADNI has been to test whether serial MRI, PET, other biological markers, and clinical and neuropsychological assessment can be combined to measure the progression of mild cognitive impairment (MCI) and early Alzheimer's disease. The subjects were recruited from over 50 sites across the United States and Canada. ADNI has undergone three stages of recruitment each with differences in the imaging and biomarker analyses, named ADNI-1, ADNI-GO, and ADNI-2. Collectively, these protocols have recruited 1631 adults into the study consisting of age-appropriate cognitively normal individuals, people with early or late MCI, and people with early Alzheimer's disease. The follow-up duration of each group is specified in the protocols for ADNI-1, ADNI-2, and ADNI-GO with 120 months as the maximum. Subjects were evaluated on entry into the study, then at the 6 and 12 month time points, and yearly after this. For up-to-date information, see www.adni-info.org.

##### Epidemiological analyses

Epidemiological analyses were performed as described by Rivers-Auty et al. (2020). Briefly, datasets containing the medical history (RECMHIST.csv), recurrent medicines (RECCMEDS.csv), and patient summary data (ADNIMERGE.csv) were downloaded on the third of May 2018. String search methods were applied to identify patients using supplements. Baseline statistics were extracted, and logistic regression was performed on Alzheimer's disease prevalence to evaluate the association between supplement use and Alzheimer's disease risk. Negative binomial generalized linear mixed modeling (GLMM) was applied to the number of failures that occurred during the Mini-Mental State Examination (MMSE). Variables were included in the models both as a fixed effect and as an interaction effect with time. Variables included in the analyses were diagnosis (control, MCI, or Alzheimer's disease), age, gender, APOE4 genotype, education level, headaches, arthritis, diabetes, smoking, cardiovascular risk factors, and supplement use. The significance of variable inclusion for the logistic regression and GLMM was evaluated using the log likelihood ratio test, estimated by a χ^2^ distribution. To construct the negative binomial GLMM models, the package glmmadmb was used (Fournier et al., 2012; Skaug et al., 2013); logistic regression used the LME4 package (Bates et al., 2015), both on R version 3.5.1 (R Core Team, 2018) with RStudio version 1.1.453 (RStudio Team, 2016). Details of the full analysis can be found in the Extended Data, which contains the annotated R code and output of the epidemiological analysis of the Alzheimer's disease neuroimaging cohort study.

10.1523/JNEUROSCI.1980-20.2020.supplement The extended data contains the annotated R code and output of the epidemiological analysis of the Alzheimer's disease neuroimaging cohort study. Download Extended data, DOCX file.

#### Animal experiments

Animals were housed in individually ventilated cages maintained at 21 ± 2°C with 40%-50% humidity on a standard 12 h light/dark cycle and free access to water and food. Before experiment, all animals were on standard rodent chow (BK001 E, Special Diets Services). Genotyping was performed by Transnetyx, and these results were not revealed until the end of all behavioral, histologic, or physiological investigations had been completed. Thus, all experiments were performed blinded to genotype. All behavioral videos, physiological analyses, and histologic quantifications were recoded to blind the experimenter to both genotype and diet. Diet was randomly allocated by cage using the TrueRandom software. All behavioral experiments were performed with 40 dB white noise played in surround sound to avoid auditory cues. All animal experiments were conducted in accordance with the United Kingdom Animals (Scientific Procedures) Act 1986 and approved by the Home Office and the local Animal Ethical Review Group, University of Manchester.

##### The effect of zinc deficiency in APP/PS1 mice

*APPswe PSEN1*Δ*E9* (*APP/PS1*) transgenic mice on a C57BL/6J background were obtained from The Jackson Laboratory (#005864). Hemizygous male *APP/PS1* mice were bred with C57BL/6J female mice to generate litters of hemizygous *APP/PS1* and C57BL/6J (WT) littermate controls, of which the males were used in the experiments. At 3 months of age, the animals underwent baseline behavioral testing and were then randomly allocated to a zinc normal (ZN) diet (35 mg/kg, Table 1) or zinc deficient (ZD) diet (3 mg/kg, Table 1). Two-thirds of the mice were initially placed on a ZD diet and one-third were placed on a ZN diet. After 3 months on diet, another set of behavioral tests were performed. Then half the cages on a ZD diet were randomly allocated to be placed on the ZN diet. All mice remained on diet for another 3 months, which was followed by another set of behavioral experiments after which mice were killed by exsanguination and cardiac perfusion with saline under isoflurane (2%-5%) anesthesia. A power analysis was performed using the standardized effect size based on an ordinary least squares repeated-measures method. A moderate partial η^2^ of 0.15 was selected with an α of 0.05 and a β of 0.2. From this, it was found that an *n* of 10 was required. To account for attrition and fluctuations in genotype prevalence, a target of *n* = 13 was selected. Final numbers (*n*) in each group were as follows: WT ZN = 13; WT ZD = 15; WT ZD/ZN = 12; *APP/PS1* ZN = 13; *APP/PS1* ZD = 11; *APP/PS1* ZD/ZN = 11.

**Table 1.** Diet composition*^a^*

Diet component	g/kg
Egg white solids	212
Maltodextrin	75
Sucrose	327
Corn starch	186.5
Corn oil	100
Cellulose	46.236
Mineral mix (zinc absent)	35
Calcium phosphate, dibasic	3
Chromium potassium sulfate, dodecahydrate	0.01
Vitamin mix AIN 93	10
Biotin	0.004
p-Aminobenzoic acid	0.11
Vitamin C, ascorbic acid, coated (97.5%)	1.017
Choline bitartrate	3
Inositol	0.11
Zinc chloride (3 mg/kg) (ZD)	0.0052
Zinc chloride (35 mg/kg) (ZN)	0.0719

*^a^*The composition of the ZN (35 mg/kg) and ZD (3 mg/kg) diets were identical apart from the amount of zinc.

##### The effect of zinc deficiency in aged C57BL/6J mice

At 3 months (Young) or 20 months (Aged) of age, C57BL/6J mice had their baseline memory performance probed using the Y-maze task, before being randomly allocated to a ZD or ZN diet for 3 months (*n* = 15 per group). All mice were then evaluated in the Y-maze and culled by exsanguination and cardiac perfusion with saline under isoflurane (2%-5%) anesthesia. Using the same power analysis as above, an *n* of 15 was selected to account for the greater potential of attrition.

##### The effect of zinc deficiency in APP/PS1 mice deficient in NLRP3

Mice lacking *nlrp3* (*nlrp3*^−/−^) on a C57BL/6J background mice were provided by Vishva Dixit (Genentech) (Mariathasan et al., 2006). These mice were backcrossed with the *APP/PS1* line for 6 generations and then bred into two colonies containing *APP/PS1/nlrp3*^−/−^ hemizygous male and WT/*nlrp3*^−/−^ (WT are C57BL/6J) female breeders, and *APP/PS1/nlrp3*^+/+^ hemizygous male and WT/*nlrp3*^+/+^ female breeders. These were used to generate the experimental male mice used in this study. At 3 months of age, cages of mice were randomly allocated onto either a ZD or ZN diet. After 6 months on diet, memory of all mice was assessed with the Y-maze task. The animals were then killed by exsanguination and cardiac perfusion with saline under isoflurane (2%-5%) anesthesia. The primary outcome of Y-maze performance after 6 months on diet was selected. Using the data obtained in the zinc deficiency experiment above, the mean difference of 13.4 and a pooled SD of 7.6 was used with an α of 0.05 and a β of 0.2. This analysis found that an *n* of 9 mice per group was required for the Sidak corrected *post hoc* analysis with 6 comparisons. To account for attrition and fluctuations in genotype prevalence, a target *n* = 10 was selected. Final numbers (*n*) in each group were as follows: WT/*nlrp3*^+/+^ ZN = 12; WT/*nlrp3*^+/+^ ZD = 14; WT/*nlrp3*^−/−^ ZN = 8; WT/*nlrp3*^−/−^ ZD = 9; *APP/PS1/nlrp3*^+/+^ ZN = 8; *APP/PS1/nlrp3*^+/+^ ZD = 11; *APP/PS1/nlrp3*^−/−^ ZN = 13; *APP/PS1/nlrp3*^−/−^ ZD = 9.

##### Y-maze task

The Y-maze consisted of three connected small rooms each with a unique visual cue on the back wall. Mice were placed in the maze using the tube transfer method and allowed to explore for 8 min. The exploration was recorded, and the videos were then recoded and scored by an observer blinded to the experimental groups. A successful set of alternations was defined as entering all three rooms in succession with no repeated room entries. Performance was expressed as the percentage of total alternation sets which were successful. The mazes were thoroughly cleaned with 70% ethanol and then dried with paper towels between animals to avoid olfactory cues. Trials in which the mouse completed <9 entries were excluded.

##### Novel smell task

The novel smell task was performed in a cylindrical arena. The mice were first habituated to the arena without olfactory cues by being placed in the arena using the tube transfer method for 8 min. The following day, the mice were placed in the arena for 8 min with two olfactory cue dispensers with identical smells. They were then placed in holding cages for 8 min, while the arenas were cleaned with ethanol and dried, and the olfactory dispensers were changed to consist of a novel and familiar smell. The mice were then placed back in the area and recorded for 4 min. The exploration was recorded, and the videos were then recoded and timed using the Novel Object Timer program (https://jackauty.com/program/) by an observer blinded to the experimental groups as described by Daniels et al. (2016). Trials in which the mouse completed <4 s of exploration were excluded.

##### Tissue processing

Mice were terminally anesthetized with 2%-5% isoflurane (30% O_2_, 70% N_2_O). Plasma was collected via cardiac puncture using citrate-treated needles, followed by trans-cardiac perfusion with 0.9% saline. Femurs were taken for inductively coupled plasma mass spectrometry of the bone, and one epididymal fat pad was removed and weighed. Brains were dissected into hemispheres. The left hippocampus was extracted and snap frozen for RNA sequencing. RNA was extracted using a QIAGEN mini-kit as per instructions and tested for purity and concentration using the nano-drop method and analyzed for purity and digestion with a TAPE station. All samples had RNA integrity numbers of >8.5. RNAseq was performed as described by Hoyle et al. (2018) on an Illumina HiSeq4000 instrument. The right hemisphere was immerse-fixed in 4% PFA for 24 h and cryoprotected in 30% sucrose before being snap frozen and sectioned (15 µm) coronally every 90 µm from the rostral end of the cortex (for experiment in Figs. 2, 3*A-C*), or immerse-fixed in 4% PFA for 24 h, then dehydrated, paraffin-embedded, and sectioned (5 µm) every 100 µm from the central sulcus (for experiment in Fig. 5) and mounted onto Superfrost Plus slides (VWR). Six sections evenly spaced throughout the cortex were selected for immunohistochemistry. Paraffin sections were deparaffinized with xylene emersion and rehydrated in descending concentrations of ethanol. For all sections, antigen retrieval consisted of 30 min immersion in 0.2 mm citrate buffer, pH 6, at 96°C followed by 10 min in 90% formic acid. Sections were washed (3 × 5 min) with 0.1% Tween in PBS (PBST), blocked for 1 h in a 1% BSA (A9647 Sigma Millipore), 0.2 m PB solution, and then incubated overnight in 1:200 biotinylated 6e10 antibody (for amyloid β [Aβ] plaques; #SIG-39 340-200, Covance) or 1:1000 Iba1 (for microglia; Wako) in a 1% BSA, 0.2 m PB solution at 4°C. Sections were then washed (3 × 5 min, PBST), incubated with biotinylated anti-mouse secondary (Iba1), or/followed by 1:20 Strep-Avidin (P188503, RnD)/1% BSA, 0.2 m PB solution for 2 h at room temperature, washed and then visualized with a DAB nickel solution (D0426-50SET, Sigma Millipore). Sections were dehydrated in serial ethanol solutions of increasing concentration and then 100% xylene, and then mounted with DPX (DI5319/05, Thermo Fisher Scientific). The slides were scanned, and the images recoded. Aβ plaque burden, approximated as percentage stained area, was calculated using threshold-particle analyses performed on ImageJ. The microglial activation stage was performed as in Daniels et al. (2016).

##### Laser microdissection

Eight- to 9-month-old *APP/PS1* and C57B/6J littermate mice were terminally anesthetized with 2-5% isoflurane (30% O_2_, 70% N_2_O) followed by trans-cardiac perfusion with 0.9% saline. Brains were harvested, snap frozen using dry ice, and stored at −80°C until analyzed. Coronal 30 μm brain sections were taken from the hippocampal region using Leica Microsystems CM3050 S cryostat and mounted onto RNase-free membrane-coated microscopy slides (Molecular Machines & Industries). The slides were stained by immersion in a filtered Congo red solution (0.2% Congo red solution in 20% NaCl, 80% ethanol, pH 9.0) for 20 min at room temperature. Using the laser microdissection (LMD) MMI (Molecular Machines & Industries) SmartCut software, UV laser excised discs of a fixed 100 μm diameter with Congo red-stained amyloid plaques in the center were cut (see Fig. 3*H*). Plaque-free tissue from both the same animals and WT littermate controls were also cut and collected separately. Once cut, microdissected regions were acquired using mechanically operated adhesive MMI Isolation Caps (Molecular Machines & Industries), which were subsequently filled with the lysis buffer component of RNeasy Micro Kit (QIAGEN). On completion of LMD, sample-containing microcentrifuge tubes immediately underwent lysis via freeze-thaw cycling and brief (<5 s) sonication. Purified RNA was acquired as per RNeasy Micro Kit (QIAGEN) instructions with on-column DNase digestion and washing according to the manufacturer's guidelines. Images were collected on an Olympus IX83 inverted microscope modified for LMD capture.

##### Inductively coupled plasma mass spectrometry

Inductively coupled plasma mass spectrometry (Agilent Technologies, 7900) was used to assess zinc levels in the plasma and bone. The diets, bedding, drinking water, and cage materials were also measured for zinc content before the start of the experiments. As a result of these analyses, the bedding was changed to the low zinc nesting material Wood Wool (TAPVEI). Plasma samples were diluted in 1:10 with 0.1 m hydrochloric acid, centrifuged (2500 × *g*), and then analyzed with appropriate standards generated. Whole femurs were taken from the mice and wet-ashed by HNO_3_ (0.5 ml, concentrated) immersion at room temperature for 24 h, then dried at 60°C. After cooling, 0.2 ml of H_2_O_2_ was added and the samples were incubated for 1 h in a drying oven at 60°C. The solutions were diluted to 10 ml using deionized water and analyzed.

##### qRT-PCR

Isolated RNA was reverse-transcribed using Tetro cDNA Synthesis Kit (Bioline), with samples being incubated at 25°C for 10 min, 45°C for 30 min, and 85°C for 5 min. cDNA was subjected to qPCR using the 7900HT Fast Real-Time PCR detection system (Applied Biosystems) Power SYBR Green Master Mix (Applied Biosystems). A standard amplification program (1 × 2 min cycle at 50°C, 1 × 10 min cycle at 95°C, 50 × 15 s cycle at 95°C, and 1 × 1 min cycle at 60°C) was used for all amplifications. Primers used for the amplification were as follows: *trem2* (forward, TGGGGACCTCTCCACCAGTT; reverse, GTGGTGTTGAGGGCTTGG), *nlrp3* (forward, GCCCAAGGAGGAAGAAGAAG; reverse, TCCGGTTGGTGCTTAGACTT), and *il-1*β (forward, AACCTGCTGGTGTGTGACGTTC; reverse, CAGCACGAGGCTTTTTTGTTGT). Standard curves of serial control cDNA dilutions were used to determine the fold differences in target cDNA expression. *Hmbs* was used as the housekeeping gene (forward, GAAATCATTGCTATGTCCACCA; reverse, GCGTTTTCTAGCTCCTTGGTAA).

#### Cell isolation and culture

##### Bone-marrow derived macrophages (BMDMs)

All primary cells were harvested from 8- to 16-week-old, mixed sex, C57/BL6J mice (Charles River), which were killed with rising CO_2_ concentration. Murine BMDMs were generated as reported previously by Daniels et al. (2016). Briefly, BMDMs were collected by flushing bone marrow from femurs and cultured in DMEM (Invitrogen) supplemented with 10% FBS (Biowest), 1% PenStrep, 2 mm, 1% L-glutamine (Sigma Millipore), and 20% macrophage colony-stimulating factor-conditioned medium from L929 cells. Cells were grown for 7 d and then scraped, counted, and seeded for 24 h in 24- to 96-well plates at 1 × 10^6^ cells/ml in complete DMEM. After media change, cells were primed with bacterial endotoxin (lipopolysaccharide [LPS], 1 µg/ml) from *Escherichia coli* O26:B6, for 4 h. The media was changed to serum-free DMEM before treatment with pathway inhibitors (MCC950 10 μm or ZnCl_2_) or vehicle (DMSO) for 15 min followed by stimuli treatment for 4 h with TPEN (N,N,N′,N′-tetrakis(2-pyridinylmethyl)−1,2-ethanediamine), or silica (<15 µm, MIN-U-SIL 15; 300 μg/ml), or 24 h for amyloid 1-42 (Sigma Millipore, oligomerized at 37°C for 24 h in PBS before treatment; NB for amyloid, cells without LPS-priming were used). Supernatants were taken for ELISA IL-1β and Western blot. An in-well lysis was performed with 1% Triton for Western blot analyses of cytosolic and secreted proteins.

##### Mixed glia

Murine mixed glia cells were prepared from the brains of 2- to 4-day-old C57BL/6J mixed sex mice. Briefly, the cerebral hemispheres were dissected, and the meninges were then removed. The remaining tissue was homogenized in DMEM containing 10% FBS and PenStrep via repeated trituration. The resulting homogenate was centrifuged at 500 × *g* for 10 min, and the pellet was resuspended in fresh culture medium, seeded directly into 24-well plates, and incubated at 37°C, 90% humidity, and 5% CO_2_. After 5 d, the cells were washed, and fresh medium was placed on the cells. The medium was then replaced every 2 d. Cells were used after 2 weeks. Cells were primed with LPS (1 μg/ml, 3 h) in DMEM containing 10% FBS and PenStrep. For zinc chelation experiments, cells were washed once with serum-free DMEM before treatment with serum-free DMEM containing vehicle (DMSO) or TPEN (20 μm, 4 h) with or without silica (300 μg/ml) or MCC950 (10 μm). For the amyloid experiment, LPS-primed cells were made zinc-deficient (TPEN, 10 μm) and incubated in amyloid oligomers (10 µg/ml, 4 h). The supernatant was centrifuged at 12,000 × *g* for 10 min before analysis for IL-1β content by ELISA.

##### ELISA and Western blot

IL-1β release was quantified by ELISA (DuoSet, R&D Systems) according to the manufacturer's instructions. IL-1β, gasdermin D, and caspase-1 cleavage was determined by Western blot. All supernatants or in-well lysates were separated by 12-well, 1.5 mm Tris-glycine SDS/PAGE and then transferred onto nitrocellulose or PVDF membranes at 25 V using a semidry Trans-Blot Turbo system (Bio-Rad). Membrane blocking was performed using in 2% BSA in 1% (v/v) Tween 20/PBS (PBST). Membranes were then incubated with primary antibodies at 4°C overnight (IL-1β AF-401, R&D Systems; gasdermin D ab209845, Abcam; and caspase-1 EPR16883, Abcam), washed and then labeled with HRP-conjugated secondary antibodies (2 h at room temperature). Target proteins were then visualized with GE HealthcareECL detection reagent (GE Healthcare) using the G:Box Chemi XX6 (Syngene) system.

##### ASC oligomerization assay

Cells were lysed in-well with 1% Triton X-100 and protease inhibitor cocktail. Cell lysates were centrifuged at 6800 × *g* for 20 min at 4°C to separate the Triton X-100-soluble and -insoluble fraction. The Triton X-100-insoluble fraction was chemically crosslinked with 2 mm disuccinimidyl suberate (Thermo Fisher Scientific) for 30 min at room temperature. Crosslinked pellets were precipitated by centrifugation at 6800 × *g* for 20 min and resuspended in Laemmli buffer. Oligomerized ASCs were then detected by Western blot.

##### ASC speck formation

Adult microglia were extracted from 8- to 16-week-old, mixed sex, C57/BL6J mice expressing ASC-citrine on a CAG promoter using MACS MicroBeads on digested brains as per themanufacturer's instructions. Microglia were seeded for 2 h into 96-well plates at 0.5 × 10^5^/ml. They were primed with LPS (1 µg/ml, 2 h). All cells were treated with the pan-caspase inhibitor Z-VAD-FMK (50 μm) before simulation to prevent caspase-dependent cell death. Without media change, NBC19 (10 μm), MCC950 (10 μm), or vehicle (DMSO) was spiked into the appropriate wells, followed by TPEN administration (final concentration 10 μm). At 15 min intervals, images were captured using a 20×/0.61 S Plan Fluor objective for a total of 12 h. Speck formation was quantified using an IncuCyte ZOOM System (Essen Bioscience). All experiments performed in serum-free OptiMEM.

#### Experimental statistics

All data are presented as mean values ± SEM unless stated. For repeated-measures analyses, linear mixed modeling was used to evaluate the significance of the effect of independent factors (genotype, diet, and time) on the dependent variable. All factors and interactions were modeled as fixed effects and mouse identifier as a random variable. A within-subject design with random intercepts was used for all models. The significance of inclusion of an independent variable or interaction terms was evaluated using log-likelihood ratio (Bates et al., 2015). Holm-Sidak *post hoc* was then performed for planned pairwise comparisons using approximated least square means (Lenth, 2016). For the remaining data, statistical analyses were unpaired Welch corrected Student's *t* test, or one-way, two-way, or three-way ANOVA test depending on the number of experimental groups and independent variables in the analyses. Holm-Sidak *post hoc* was then performed for planned pairwise comparisons. Homoscedasticity and normality were evaluated graphically using predicted versus (Pearson) residuals and Q-Q plots, respectively, and transformations and/or corrections were applied where necessary. All analyses were performed using R (version 3.3.3) (R Core Team, 2018; RStudio Team, 2016).

## Results

### Zinc supplementation use is associated with reduced prevalence of Alzheimer's disease and slower cognitive decline during disease development

**Figure 1.**
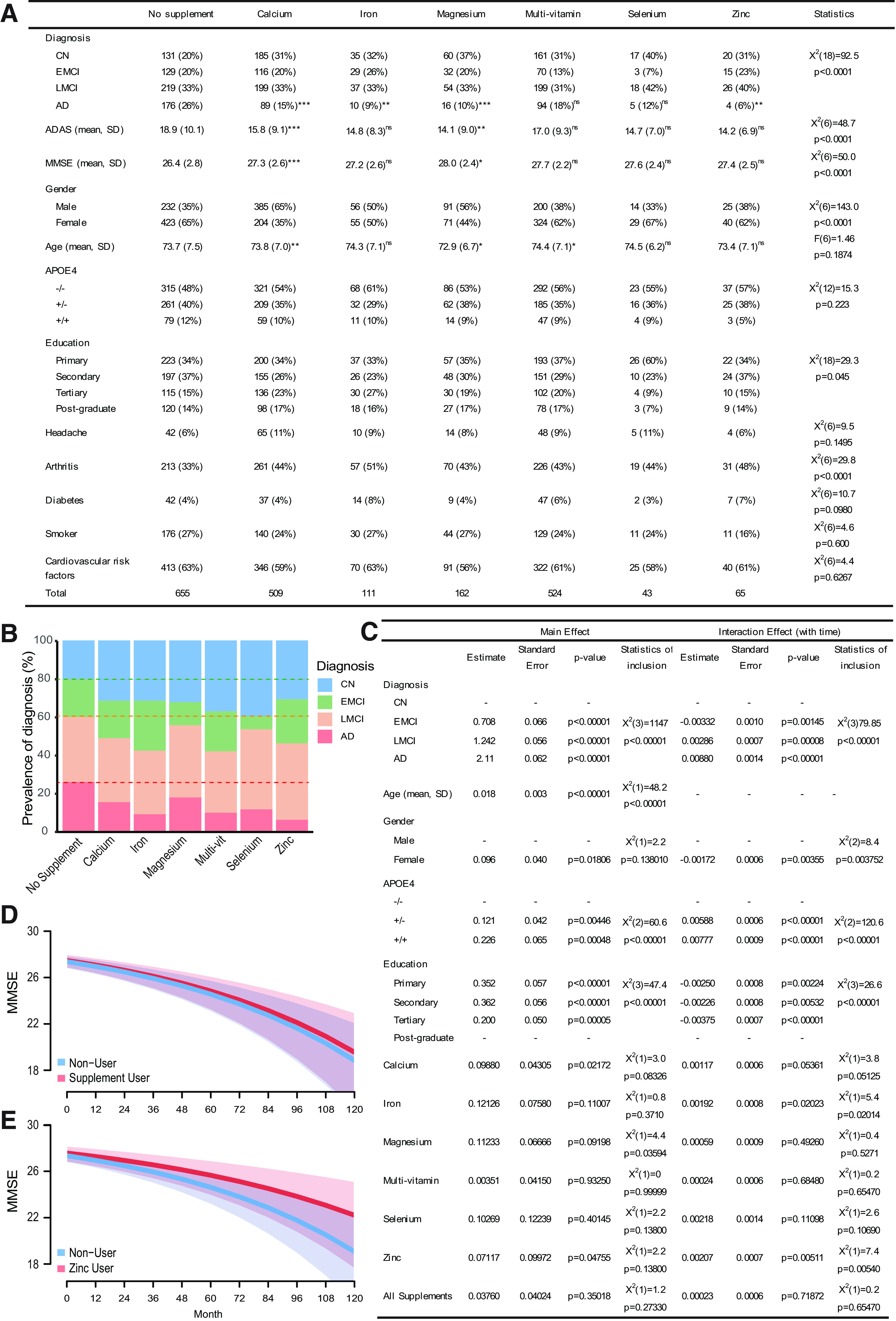
Zinc supplementation use was associated with reduced prevalence of Alzheimer's disease and slowed cognitive decline during disease development. ***A***, Table of baseline statistics by supplement showing nominal differences in nuisance variables, such as age, gender, APOE4 status, and significant differences in the prevalence of cognitive dysfunction. Shown are the number of participants for each variable with percentage of total in parentheses; or for numerical variables, the means and SD are shown. *p < 0.05; **p < 0.01; ***p < 0.001; compared with no supplement use. “Statistics” column reports the omnibus tests. ***B***, A stacked bar chart showing the proportions of cognitive diagnosis. Zinc supplement use was associated with the lowest proportion of Alzheimer's disease. This was significantly different from “no supplement” use group as reported in ***A***. Dashed lines indicate proportions of cognitive diagnoses in the “no supplement” group. ***C***, Summary table of the final negative binomial GLMM with MMSE failures as the dependent variable. Zinc supplement use was associated with significantly slowed cognitive decline. Shown are the maximum likelihood estimates (“Estimate” columns) with Laplace estimates of the SE (“Standard Error” columns), *Z* value and *p* value from the Wald approximation (“p-value” columns), as well as, the significance of inclusion of the variable in the model evaluated using the log-likelihood ratio test, χ^2^ (“Statistics of inclusion” columns). ***D***, ***E***, The effect of supplement use on predicted cognitive decline of an LMCI, 70-year-old female as measured by MMSE scores. Plots model the effect of zinc only supplement use (***E***) and any supplement (combined) use (***D***) with no supplement use modeled as the control group in both plots. CN, Cognitively normal; EMCI, early MCI; LMCI, late MCI; AD, Alzheimer's disease; ADAS, Alzheimer's Disease Assessment Scale. ns, not significant

The ADNI dataset was used to investigate the effects of mineral supplementation on both the prevalence of cognitive dysfunction, including early and late MCI, and Alzheimer's disease, as well as the progression of cognitive decline over time. Investigating the six most common mineral supplements (calcium, iron, magnesium, multivitamin, selenium, zinc), and using logistic regression adjusting for common confounding variables, we found that calcium, iron, magnesium, and zinc supplement use was associated with significantly reduced Alzheimer's disease prevalence compared with those not taking any supplements (Fig. 1*A*,*B*). To further investigate the potential effects of mineral supplementation, innovative statistical methods for analyzing cognitive decline, as measured by the MMSE, were used. From this, it was found that zinc supplementation was associated with significantly less cognitive decline (Fig. 1*C*,*E*). Other supplements, including the divalent cations calcium and magnesium, were not associated with significant changes in cognitive decline (Fig. 1*D*).

### Subclinical zinc deficiency accelerates memory deficits in an animal model of Alzheimer's disease without effecting plasma zinc or weight gain

**Figure 2.**
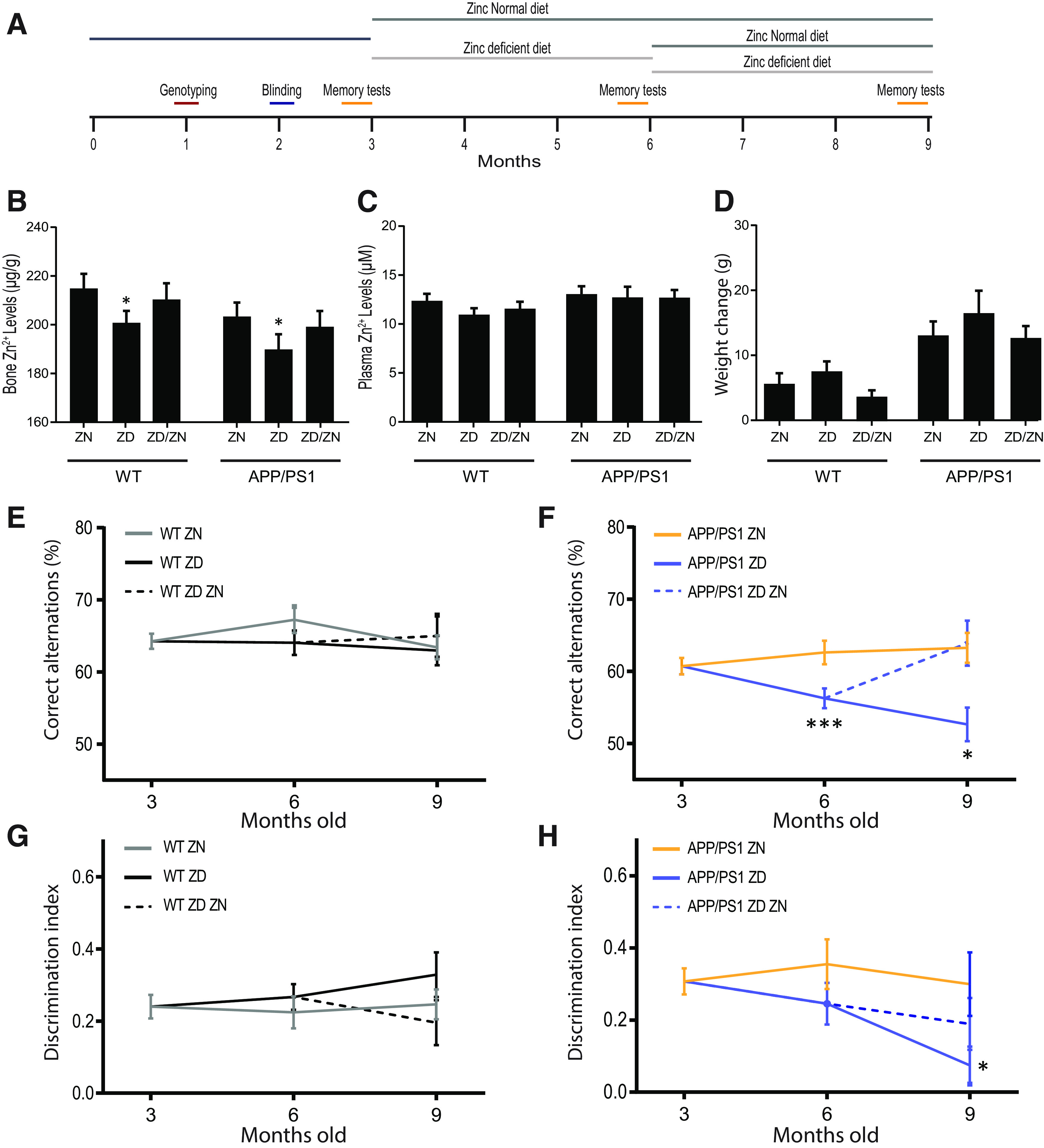
Subclinical zinc deficiency accelerates memory deficits in an animal model of Alzheimer's disease without effecting plasma zinc or weight gain. ***A***, Timeline of the zinc deficiency experiment in *APPswe/PS*δ*E9 (APP/PS1)* and WT mice. ***B***, ***C***, Inductively coupled plasma mass spectrometry revealed that 6 months on a ZD diet (3 mg/kg) induced sustained subclinical zinc deficiency as measured by reduced bone zinc levels (***B***) but not plasma zinc levels (***C***). ***D***, ZD diet did not affect growth of the animals with no effect on body weight change during the course of the study. ***E***, ***F***, The Y-maze memory task revealed that a ZD diet induced a significant memory deficit in the *APP/PS1* mice compared with mice on a ZN diet (35 mg/kg). These deficits were reversed by returning these mice onto a ZN for 3 months (ZD/ZN group; ***F***). ***G***, ***H***, The novel smell recognition task revealed significant deficits in the *APP/PS1* ZD group compared with the ZN group (***H***). Animals returned to the ZN diet at 6 months old did not return to cognitive normal performance. In WT mice, no deficits were observed in the Y-maze or the novel smell recognition tasks on any diet combination (***E***,***G***). *p < 0.05; ***p < 0.001; compared with the ZN group within the same genotype. Holm-Sidak-corrected *post hoc* analysis. Data are mean ± SEM. WT ZN, *n* = 13; WT ZD, *n* = 15; WT ZD/ZN, *n* = 12; *APP/PS1* ZN, *n* = 13; *APP/PS1* ZD, *n* = 11; *APP/PS1* ZD/ZN, *n* = 11.

We then sought to establish that the association between zinc supplementation and improved Alzheimer's disease outcome was causal by using the *APP/PS1* mouse model of Alzheimer's disease. WT control or *APP/PS1* mice were fed a diet containing the recommended zinc intake levels for mice (35 mg/kg, ZN) or a diet that would induce a mild subclinical zinc deficiency (3 mg/kg, ZD) (Fig. 2*A*). Animals were put on diet from 3 months of age and maintained for 6 months with memory tests (Y-maze and novel smell test) performed at 3 month intervals (Fig. 2*A*). To determine whether any effects of zinc deficiency were reversible, one group of mice was maintained on a ZD diet for 3 months and then placed on a normal zinc diet for 3 months (Fig. 2*A*). *APP/PS1* mice do not show altered cognitive behavior in short-term memory tasks, such as the Y-maze, T-maze, or novel object recognition before 9 months of age (Webster et al., 2014). At the end of the experimental period, animals were killed, zinc levels measured in the plasma and bone, and a number of histologic and physiological measures were taken, including RNAseq on hippocampal tissue, and analyses on Aβ plaque burden and microglia morphology. There was no change in plasma zinc between all groups (diet main effect: *F*_(2,68)_ = 0.86, *p* = 0.43), but a decrease in zinc content in the bone was seen in WT and *APP/PS1* mice fed a ZD diet, indicating a change in long-term zinc status (Fig. 2*B*,*C*) (diet main effect: *F*_(2,64)_ = 4.26, *p* = 0.018). Severe zinc deficiency can lead to weight loss, but the marginal ZD diet used here had no effect on body weight compared with normal diet (Fig. 2*D*) (diet main effect: *F*_(2,43)_ = 0.49, *p* = 0.616) or fat mass (data not shown, diet main effect: *F*_(2,74)_ = 0.14, *p* = 0.866) of the WT or *APP/PS1* mice. These data suggest that we had modeled a subclinical level of zinc deficiency (Beattie et al., 2012).

The mild zinc deficiency causes reversible cognitive decline in the *APP/PS1* mice while having no effect on the cognitive performance of the WT mice (Y-maze task three-way interaction: χ^2^_(6)_ = 19.33, *p* = 0.004). In WT mice, there was no effect of ZD diet on memory over 6 months as analyzed by the Y-maze (*Z* = −0.16, *p* = 1.000) and novel smell tests (*Z* = −0.16, *p* = 0.743) (Fig. 2*E*,*G*). As expected, the *APP/PS1* mice showed no significant reduction in cognitive performance over the course of the experiment on the ZN diet in the Y-maze (*Z* = −1.81, *p* = 0.281) or novel smell tests (*Z* = −0.05, *p* = 1.000) (Fig. 2*F*,*H*). However, *APP/PS1* mice on a ZD diet showed accelerated memory loss at 3 months in the Y-maze test (*Z* = −2.81, *p* = 0.024), and this was worse after 6 months on the ZD diet (*Z* = −3.75, *p* = 0.001) (Fig. 2*F*). This cognitive impairment measured by Y-maze of *APP/PS1* mice fed a ZD diet for 3 months was reversed by returning the mice to a ZN diet for 3 months (*Z* = −3.93, *p* < 0.001) (Fig. 2*F*). Similar, but less dramatic, trends were observed in the novel smell test in response to a ZD diet in the *APP/PS1* mice (Fig. 2*H*) (three-way interaction: χ^2^_(6)_ = 14.53, *p* = 0.024). These data suggest that zinc deficiency is causally accelerating cognitive decline in Alzheimer's disease and that these cognitive deficits are reversible. They also place our epidemiological observations in people in context, suggesting that the association of improved cognitive outcome in patients taking zinc supplements is because of a direct causal relationship between zinc status and Alzheimer's disease progression.

### Zinc deficiency does not alter plaque burden, microglia activation, or age-related cognitive decline

**Figure 3.**
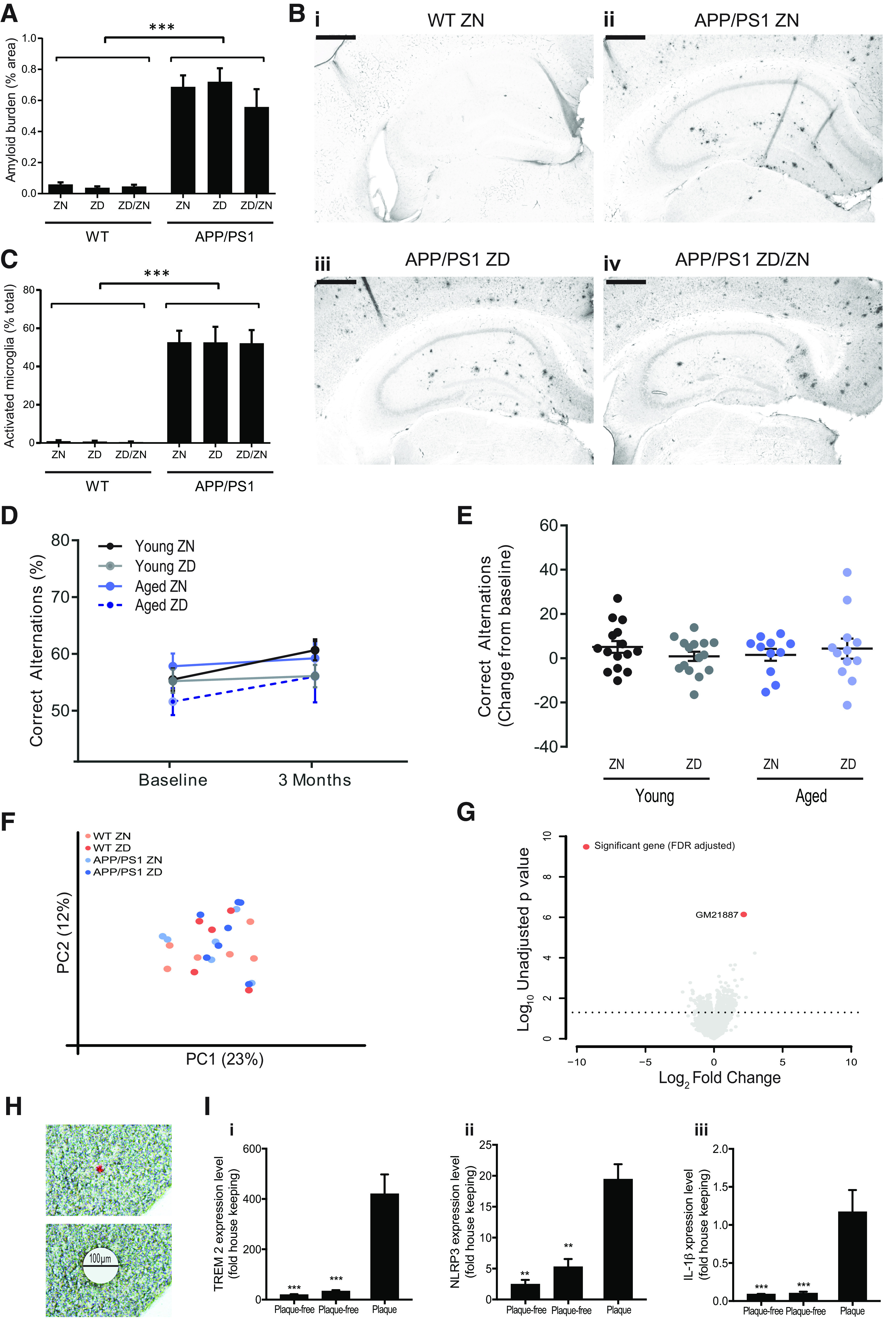
The effects of zinc deficiency alter the local response to the plaque without altering plaque burden or age-related cognitive decline. ***A***, Six months on a ZD (3 mg/kg) diet did not alter Aβ plaque burden in *APPswe/PS*δ*E9 (APP/PS1)* mice compared with ZN (35 mg/kg) controls. The zinc recovery group was also unchanged (ZD diet for 3 months followed by a ZN diet for 3 months; ZD/ZN). ***B***, Example images of Aβ plaque burden. ***C***, No differences were observed in microglia morphology between any diet combination, measured as percentage of microglia in an amoeboid phenotype. ***A-C***, Sections were incubated with 6e10 (Aβ) or Iba1 (microglia) antibodies, visualized with DAB nickel (3,3′-diaminobenzidine, nickel sulfate) staining averaged across 6 coronal hippocampal sections per mouse. Scale bars, 500 µm. ***p < 0.001, main effect of genotype (two-way ANOVA). ***D***, ***E***, Unlike in the *APP/PS1* mouse, zinc deficiency did not accelerate age-related cognitive decline, as measured by the Y-maze, in 20-month-old C57B/6J mice placed on a ZD diet for 3 months. Similarly, no changes were observed in the 3-month-old “young” C57B/6J controls. This suggested that zinc deficiency was altering an amyloid-specific response, such as NLRP3-dependent inflammation. ***F***, ***G***, Zinc content in the diet had no effect on the transcriptome in whole hippocampal tissue of *APP/PS1* and WT mice. ***F***, A principal component analysis (PCA) of the transcriptome data showing no clustering of the WT or *APP/PS1* mice or the ZD and ZN diet. ***G***, Volcano plot of genes comparing *APP/PS1* mice on a ZN or ZD diet showing minimal effects of diet with only one gene found to be significantly different following false discovery rate (FDR) adjustment (*n* = 6/group). ***H***, ***I***, To establish whether there was a local response in *APP/PS1* mice, periplaque regions (100 µm diameter) and plaque-free tissue from 8- to 9-month-old *APP/PS1* mice were isolated using laser capture (***H***) and probed for the plaque-associated gene *trem2* (***Ii***) and the inflammatory genes *nlrp3* (***Iii***) and *il-1*β (***Iiii***); and from this, it was found that the *nlrp3/il-1*β response is very tightly associated to the periplaque region (*n* = 3, for all groups). **p < 0.01; ***p < 0.001; versus plaque containing region. Plaques were identified with Congo red staining. Holm-Sidak-corrected *post hoc* analysis. Data are mean ± SEM. ***A-C***, WT ZN, *n* = 13; WT ZD, *n* = 15; WT ZD/ZN, *n* = 12; *APP/PS1* ZN, *n* = 13; *APP/PS1* ZD, *n* = 11; *APP/PS1* ZD/ZN, *n* = 11. ***D***, ***E***, Young ZN, *n* =15; Young ZD, *n* = 15; Aged ZN, *n* = 11; Aged ZD, *n* = 12 (<15 because of attrition).

The effects of zinc deficiency on memory were not because of altered Aβ plaque burden as plaque burden was not significantly different between *APP/PS1* mice fed a ZN or a ZD diet (Fig. 3*A*,*B*) (two-way interaction: *F*_(3,61)_ = 1.93, *p* = 0.154). Similarly, levels of microglia with activated morphology, typically associated with plaques, were not altered between treatment groups (Fig. 3*C*) (two-way interaction: *F*_(3,49)_ = 0.06, *p* = 0.940). We then hypothesized that the effects of zinc deficiency may be common with NLRP3-dependent age-related cognitive decline (Youm et al., 2013). Thus, we placed 20-month-old C57BL/6J mice on a ZD diet for 3 months, with 3-month-old mice as young controls. No acceleration in cognitive decline after a ZD diet was observed at either age in the Y-maze task (three-way interaction: χ^2^_(7)_ = 7.34, *p* = 0.394) or Morris water maze task (interaction term: *F*_(1)_ = 0.284, *p* = 0.570), indicating that the effect of zinc deficiency seen in the *APP/PS1* mice was specific to amyloidopathy and its downstream effects (Fig. 3*D*,*E*). To elucidate the mechanism of zinc deficiency-induced cognitive decline in *APP/PS1* mice, RNAseq was performed on the whole hippocampal homogenate. However, no substantial differences in transcriptome were observed between genotype or diet (Fig. 3*F*,*G*). We hypothesized therefore that transcriptional differences could be occurring primarily in the periplaque regions, which were masked in the hippocampal RNAseq data (Fig. 3*F*,*G*) by homogenizing the whole tissue. To investigate this potential locality of transcriptional changes, we utilized laser capture microdissection to probe gene expression in the periplaque regions of 8- to 9-month-old *APP/PS1* mice (Fig. 3*H*). Performing qPCR on the established the Alzheimer's disease-linked gene *trem2* and the inflammatory genes for *nlrp3* and *il-1*β, we confirmed that transcriptional changes were largely confined to the periplaque regions (Fig. 3*I*) (periplaque main effect: *trem2* χ^2^_(1)_ = 28.40, *p* < 0.001; *il-1*β χ^2^_(1)_ = 32.21, *p* < 0.001; *nlrp3* χ^2^_(1)_ = 13.93, *p* < 0.001). Given that NLRP3 activation has been strongly linked to memory impairment in Alzheimer's disease (Heneka et al., 2013; Daniels et al., 2016) and that NLRP3 is activated by zinc deficiency (Summersgill et al., 2014), we hypothesized that accelerated memory impairment in the *APP/PS1* mice on the ZD diet is because of a periplaque *nlrp3* response (Heneka et al., 2013; Daniels et al., 2016; Dempsey et al., 2017; Venegas et al., 2017; White et al., 2017).

### Zinc deficiency activates the NLRP3 inflammasome and potentiates NLRP3 responses to stimuli, while zinc supplementation inhibits NLRP3 activation

**Figure 4.**
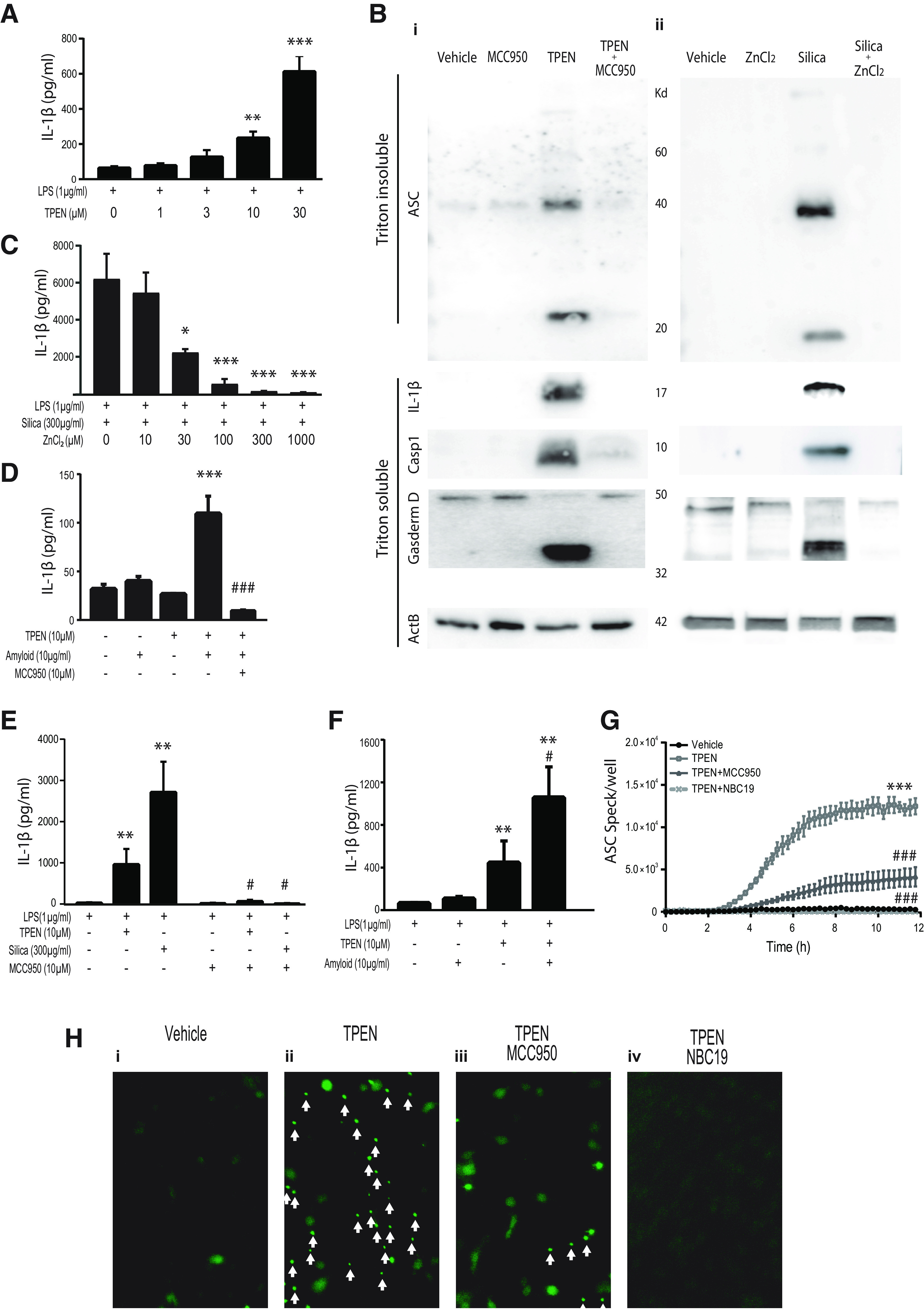
Zinc deficiency activates the NLRP3 inflammasome and potentiates NLRP3 responses to stimuli, while zinc supplementation inhibits NLRP3 activation. ***A-D***, Primary BMDM experiments demonstrating that zinc deficiency activates the NLRP3 inflammasome and zinc supplementation inhibits NLRP3 inflammasome activation. ***A***, Enhanced IL-1β release from ZD macrophages (BMDM), which were primed with LPS (1 µg/ml, 4 h) and then treated with increasing concentrations of the zinc chelation agent TPEN for 4 h. ***B***, BMDMs were primed (LPS 1 µg/ml, 4 h) and treated with TPEN (10 μm) with and without NLRP3 inhibition by MCC950 (10 μm) (***Bi***) or silica (300 µg/ml) with and without ZnCl_2_ (200 μm) supplementation (***Bii***); the cells were lysed in the media and probed for ASC oligomerization, IL-1β cleavage (17 kDa), (casp 1) activation (p10), and gasdermin D cleavage. ***C***, Zinc supplementation inhibits IL-1β release; BMDMs were primed (LPS 1 µg/ml, 4 h) and treated with silica (300 µg/ml) in the presence of increasing concentrations of ZnCl_2_. ***D***, BMDMs treated with TPEN (10 μm) and amyloid oligomers (10 µg/ml, NB no LPS priming) for 24 h release IL-1β, which is inhibited by MCC950 treatment (10 μm). *p < 0.05; **p < 0.01; ***p < 0.001; versus no treatment or LPS control. ^###^*p* < 0.001 versus activated group (Amyloid+TPEN). ***E***, ***F***, Confirmation experiments in mixed glial cultures demonstrating that LPS-primed BMDMs and mixed glial cultures have similar NLRP3 responses to zinc deficiency. ***E***, ZD primary mixed glia release IL-1β in an NLRP3-dependent manner; primary murine mixed glia were primed with LPS (1 µg/ml, 4 h) and then treated with TPEN (10 μm) or silica (300 µg/ml) for 4 h with and without the NLRP3 inhibitor MCC950 (10 μm). ***F***, LPS (1 µg/ml, 4 h) primed mixed glia release more IL-1β in response to amyloid oligomers (10 µg/ml, 4 h) when zinc deficiency is induced (TPEN, 10 μm). **p < 0.01 versus LPS alone control. ^#^*p* < 0.05 versus activated group (LPS/silica or LPS/TPEN). ***G***, ***H***, ZD (TPEN 10 μm) primary ASC-citrine expressing microglia (primed with LPS; 1 µg/ml, 2 h) form inflammasome specks over time (***G***,***Hii***, white arrows), which is inhibited by the NLRP3 inhibitors MCC950 (10 μm) (***Hiii***) and NBC19 (2 μm) (***Hiv***). ***p < 0.001 versus vehicle control (LPS alone). ^###^*p* < 0.001 versus activated group (LPS/TPEN). Holm-Sidak-corrected *post hoc* analysis following one-way (***A***,***C***,***D***), two-way (***E***,***F***), or repeated-measures (***G***) ANOVA. Data are mean ± SEM of 4 independent experiments.

To investigate the potential regulation of NLRP3 by zinc status, *in vitro* methods were used. We previously established that primary BMDMs have similar NLRP3 responses as microglia, mixed glial cultures, and *ex vivo* brain tissue slices, therefore represent a robust and high throughput method for investigating NLRP3 activation (Daniels et al., 2016; Hoyle et al., 2020). BMDMs were primed with LPS and then treated with the zinc chelator TPEN (1-30 μm, 4 h) to cause zinc depletion. Analysis of the supernatant showed TPEN-induced zinc depletion induced-1β release in a concentration-dependent manner (*F*_(4,20)_ = 25.28, *p* < 0.001) (Fig. 4*A*). Zinc depletion (TPEN 10 μm, 4 h) in LPS-primed BMDMs also caused oligomerization of ASC, caspase-1 activation, and gasdermin-D cleavage, and this was NLRP3-dependent as it was inhibited by the selective NLRP3 inhibitor MCC950 (Fig. 4*Bi*). Conversely, zinc supplementation (with ZnCl_2_) in LPS-primed BMDMs blocked established NLRP3 activators, including silica, by inhibiting IL-1β release (*F*_(5,12)_ = 122.0, *p* < 0.001), ASC oligomerization, caspase-1 and gasdermin D cleavage (Fig. 4*Bii*,*C*). We then investigated potential interactions between Aβ and zinc depletion. Using BMDMs, we found that only Aβ42 oligomer treatment followed by TPEN was sufficient to induce IL-1β secretion, which was inhibited by MCC950 (Fig. 4*D*). The effects of TPEN were then confirmed in LPS-primed mixed glial cultures, and again TPEN treatment induced IL-1β release, which was inhibited by MCC950 (*F*_(5,12)_ = 20.18, *p* < 0.001) (Fig. 4*E*). Using LPS-primed mixed glia, we also found that TPEN was also able to exacerbate Aβ42 oligomer-induced IL-1β secretion (*F*_(3,8)_ = 8.06, *p* = 0.008) (Fig. 4*F*). To investigate whether TPEN-induced zinc depletion caused inflammasome specks in microglia, ASC-citrine-expressing microglia were isolated from adult mice (Tzeng et al., 2016). Zinc depletion resulted in speck formation, which was observed with live cell imaging and the NLRP3 inhibitors MCC950 (Coll et al., 2015) and NBC19 (Baldwin et al., 2017) were found to inhibit ASC speck formation (Fig. 4*G*,*H*) (MCC950, *Z* = −6.34, *p* < 0.001; NBC19, *Z* = −26.80, *p* < 0.001). Collectively, these experiments indicate that zinc status can influence NLRP3 activity. Zinc deficiency acts as an NLRP3 activating and potentiating stimuli in macrophages, microglia, and mixed glia, while zinc supplementation is inhibitory of NLRP3 activation. Critically, we found that the combination of zinc deficiency and amyloidopathy is sufficient to induce NLRP3 activation and IL-1β release in mixed glia (and macrophages).

### Zinc deficiency accelerates cognitive decline in the APP/PS1 mouse model of Alzheimer's disease through an NLRP3-dependent mechanism

**Figure 5.**
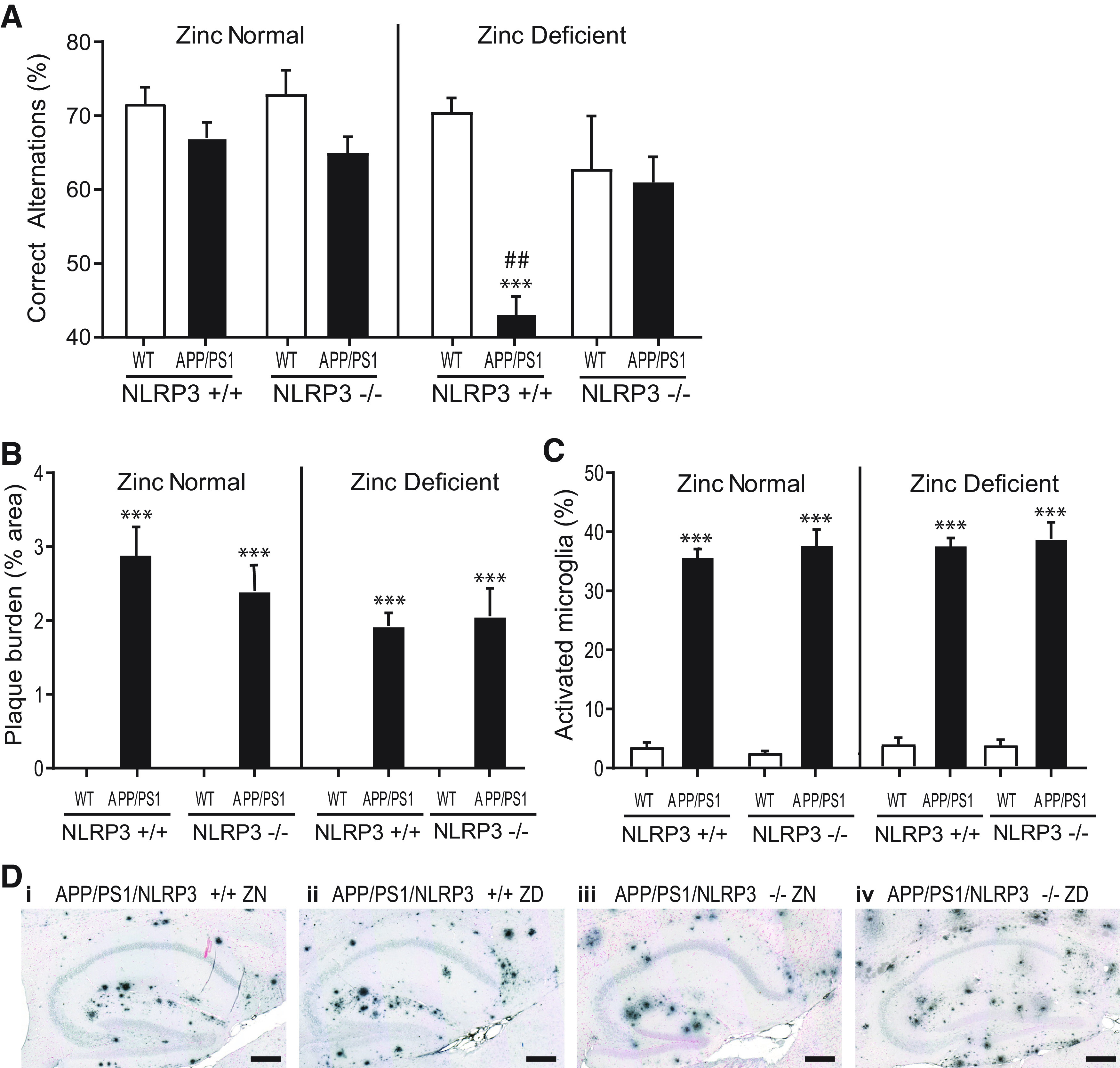
Zinc deficiency accelerates cognitive decline in the *APP/PS1* mouse model of Alzheimer's disease through an NLRP3-dependent mechanism. ***A***, *APP/PS1* and C57BL/6J (WT) littermate mice were crossed with *nlrp3*^−/−^ mice. Three-month-old mice from each genotype combination were placed on a ZD (3 mg/kg) or ZN (35 mg/kg) diet for 6 months and were then assessed using the Y-maze memory task. Memory deficits were seen in the *APP/PS1* mice on a ZD diet but not on a ZN diet. However, *APP/PS1*/NLRP3^−/−^ mice on a ZD diet did not have memory deficits. ***B***, ***D***, No effect of diet or genotype was seen on Aβ plaque burden in *APP/PS1* mice, or on (***C***) microglia morphology measured as percentage of microglia in an amoeboid phenotype in adjacent sections. ***B***, ***C***, Sections were incubated with 6e10 (Aβ) and Iba1 (microglia) antibodies, respectively, visualized with DAB nickel (3,3′-diaminobenzidine, nickel sulfate) staining averaged across 6 sagittal hippocampal sections per mouse. Scale bars, 250 µm. ***p <0.001 versus WT littermates of the same NLRP3 genotype and diet. ^##^*p* < 0.01 versus *nlrp3*^−/−^ group of the same diet and *APP/PS1* genotype conditions. Holm-Sidak-corrected *post hoc* analysis following three-way ANOVA (NLRP3 genotype, diet, APP/PS1 genotype). Data are mean ± SEM. WT/*nlrp3*^+/+^ ZN, *n* = 12; WT/*nlrp3*^+/+^ ZD, *n* = 14; WT/*nlrp3*^−/−^ ZN, *n* = 8; WT/*nlrp3*^−/−^ ZD, *n* = 9; *APP/PS1/nlrp3*^+/+^ ZN, *n* = 8; *APP/PS1/nlrp3*^+/+^ ZD, *n* = 11; *APP/PS1/nlrp3*^−/−^ ZN, *n* = 13; *APP/PS1/nlrp3*^−/−^ ZD, *n* = 9.

As we had established that zinc deficiency could potentiate NLRP3 responses *in vitro*, we hypothesized that zinc deficiency accelerated cognitive impairment in the *APP/PS1* mouse via the NLRP3 inflammasome. To test this hypothesis, we crossed WT and *APP/PS1* mice with *nlrp3*^−/−^ mice to generate four experimental groups of mice: WT, *nlrp3*^−/−^, *APP/PS1*, and *APP/PS1/nlrp3*^−/−^. Three-month-old animals were fed either a ZN or ZD diet for 6 months before memory assessment using the Y-maze. As above, there was no effect of a ZD diet on the memory of WT mice (*t*_(72)_ = −0.33, *p* = 1.000) or in *nlrp3*^−/−^ mice (*t*_(72)_ = −0.81, *p* = 1.000) (Fig. 5*A*). Also, as previously noted, the ZD diet caused memory impairment in *APP/PS1* mice (*t*_(72)_ = −8.06, *p* < 0.001) (Fig. 5*A*). However, the zinc deficiency-induced memory impairment in *APP/PS1* mice was abolished in *APP/PS1/nlrp3*^−/−^ mice (*t*_(72)_ = 4.54, *p* < 0.001) (Fig. 5*A*). These data suggest that the effects of zinc deficiency on memory depend on NLRP3. These protective effects of NLRP3 deficiency on memory after zinc deficiency were independent of plaque burden or microglia activation as there was no significant difference in these parameters in *APP/PS1* mice with or without *nlrp3* (three-way interaction term: plaque *F*_(1,46)_ = 1.15, *p* = 0.290; microglia *F*_(1,46)_ = 0.49, *p* = 0.488) (Fig. 5*B–D*).

## Discussion

Using patient data, animal models, and cell culture methods, we have provided evidence that zinc status is important in Alzheimer's disease progression, and specifically that zinc deficiency accelerated cognitive decline in an Alzheimer's disease model through potentiating inflammatory responses mediated by the NLRP3 inflammasome.

Our epidemiological evidence is supported by numerous previous studies which report that people with Alzheimer's disease have lower zinc serum levels (Ventriglia et al., 2015). Ventriglia et al. (2015) performed a meta-analysis resulting in a pooled sample of 1064 people with Alzheimer's disease and 1894 healthy controls and found that the serum or plasma zinc levels were lower in people with Alzheimer's disease with a standard mean difference of −0.39, providing initial evidence of a cause association of zinc deficiency and Alzheimer's disease. The present study takes this result further, demonstrating reduced cognitive decline over time in people with MCI and Alzheimer's disease in individuals who are taking zinc supplements. While we acknowledge that this association could be explained by an unaccounted hidden variable, including lifestyle factors or unknown disease-modifying medicines, most other mineral supplements were not associated with an improvement in cognitive decline. To probe this association and establish whether zinc status had a causal effect on cognitive decline, we conducted *in vivo* experiments using animal models of amyloidopathy.

There is clinical and preclinical evidence that excessive zinc supplementation can lead to health complications by being directly toxic, inhibiting copper absorption and through an interaction with amyloidopathy mechanisms (Fosmire, 1990; Yang et al., 2013; Flinn et al., 2014; Craven et al., 2018). There is also robust evidence for high zinc deficiency prevalence among the elderly and in particular people with Alzheimer's disease; hence, we hypothesize that the association of zinc supplementation with improved cognitive outcomes reported here was likely because of the mitigation of zinc deficiency, rather than zinc supplementation exceeding recommended daily intake values (Briefel et al., 2000; Ventriglia et al., 2015). Therefore, we designed the *in vivo* experiments to investigate the effects of mild subclinical zinc deficiency on cognitive decline in the *APP/PS1* mouse model of Alzheimer's disease and addressed whether any effects could be reversed by normalizing the levels of zinc. From this, we found that ZD *APP/PS1* mice had accelerated cognitive decline, but these deficits could largely be reversed by returning the mice to a diet that contained the recommended daily intake of zinc.

The literature describing the effects of zinc on amyloid deposition is complex (Esler et al., 1996; Brown et al., 1997; Garai et al., 2007; Rezaei-Ghaleh et al., 2011; Abelein et al., 2015; Lee et al., 2018). We report no changes in plaque deposition induced through zinc deficiency; therefore, we hypothesized that zinc was modulating the response to the Alzheimer's amyloidopathy modeled by the *APP/PS1* mice and not the amyloidopathy itself. Using the same animal model, Stoltenberg et al. (2007) reported a mild increase in plaque size in ZD animals. However, the zinc deficiency induced by Stoltenberg et al. (2007) was more severe, resulting in a 20% reduction in serum zinc levels. The present study induced a mild subclinical zinc deficiency, which caused no change to serum zinc levels and was only detected through reduced zinc levels in the bone. Brain levels of zinc were not measured here because of the complication of amyloid plaques sequestering zinc. Indeed, Stoltenberg et al. (2007) could not detect changes in cortical zinc even with the more severe zinc deficiency likely because of this sequestration effect.

We saw an increase in *nlrp3* expression in the periplaque region in the hippocampus of the *APP/PS1* mice. This observation and previous research support an importance of NLRP3 in the response to amyloidopathy (Heneka et al., 2013; Daniels et al., 2016). Using cellular models, we demonstrated that zinc deficiency potentiated NLRP3 responses to stimuli, including amyloid oligomers and that zinc inhibited NLRP3 responses to activators, such as silica. The concentration of amyloid used was supraphysiological to observe measurable inflammatory responses in the short time periods possible with the culture methods utilized. This limitation was addressed by translating these results *in vivo*. We established NLRP3 as the critical mechanism by which zinc deficiency accelerated cognitive decline in the *APP/PS1* mouse model through crossing *nlrp3*^−/−^ and *APP/PS1* mouse lines. We demonstrated that the cognitive decline induced by zinc deficiency was completely abated in the *APP/PS1* mice deficient in NLRP3. Interestingly, we saw no changes in amyloid deposition in the *APP/PS1/nlrp3*^−/−^ mice. Heneka et al. (2013) generated the same mouse strain and did show reduced plaque burden; however, in that study, 16-month-old mice were used. Therefore, it may be the later stages of the pathology in which NLRP3 plays a role in plaque deposition. Given the excellent research linking NLRP3 to the aging process (Youm et al., 2013), the age difference between Heneka et al. (2013) and the present study could explain this difference.

Previous research has demonstrated that zinc chelation with clioquinol impedes the matrix metallopeptidase 9 processing of pro-BDNF, perturbing synapse formation and cognitive performance (Frazzini et al., 2018). Furthermore, zinc supplementation in the 3xTg mouse model of Alzheimer's disease was found to prevent cognitive decline through increasing BDNF levels and mitigating tau, amyloid, and mitochondrial Alzheimer's disease-associated pathology (Corona et al., 2010). The RNAseq performed here found no effects of zinc deficiency on expression of genes downstream of BDNF signaling (e.g., neuropeptide Y). One explanation for this incongruence is the time frame and severity of zinc deficiency. Zinc chelation likely induced a more severe reduction in bioavailable zinc than what was observed in the present study. Additionally, Corona et al. (2010) administered zinc supplementation to mice for 11 months, starting at 1-month old mice. This was both a longer period than the present study and began at an age where neurodevelopment is ongoing. So, while in the present study we see no evidence of BDNF modulation, it is likely this pathway is affected in other modalities of zinc deficiency and supplementation.

Managing Alzheimer's disease is currently one of the greatest societal problems. An increasing prevalence linked to an aging population, with limited treatment options, is creating a global crisis. The NLRP3 inflammasome has been identified as a therapeutic target for the treatment of Alzheimer's disease, and there are many pharmaceutical and academic initiatives underway to develop new small-molecule inhibitors of the NLRP3 inflammasome (e.g., MCC950 and NBCs) (Coll et al., 2015; Baldwin et al., 2017). The possibility of drug repurposing has also been identified for targeting NLRP3 in Alzheimer's disease (Daniels et al., 2016). Here, we show that, coupled to these initiatives, the nutritional status, particularly of zinc, could be assessed and used to treat groups of people with Alzheimer's disease shown to be at risk of zinc deficiency. In theory, zinc supplementation or dietary improvements could be a cheap, rapid, low-risk, and easy enhancement to patient care.
