# Supplementary material for: Zinc Status Alters Alzheimer's Disease Progression through NLRP3-Dependent Inflammation
Source: J Neurosci. 2021 Mar 31;41(13):3025–38. doi: 10.1523/JNEUROSCI.1980-20.2020 (PMC8018890; doi:10.1523/JNEUROSCI.1980-20.2020)
Supplement: Supplementary file 1 — The extended data contains the annotated R code and output of the epidemiological analysis of the Alzheimer's disease neuroimaging cohort study. Download Extended data, DOCX file. [file ns-JN-RM-1980-20-s01.docx]

**Extended data**

Full compiled analyses of the epidemiological ADNI dataset including R code used are included below.

# Epidemiology

## Packages use

require(knitr)

require(lme4)

require(LMERConvenienceFunctions)

require(lmerTest)

require(MASS)

require(bbmle)

require(R2admb)

require(glmmADMB)

require(coefplot)

require(car)

require(corrplot)

library(reshape2)

## Data Cleaning

### Recurrent medicine data set

The RECCMEDs data set contains all regularly taken medicines.

Meds<-read.csv("C:/Users/jrivers/Dropbox/Science/Projects/Epidemiology ADNI/Analysis/RECCMEDSnew.csv", header=T)

#### Data Cleaning Oral Route only

There are multiple routes of administration of supplements, however, topical skin application will fail to reach biological relevant concentrations in the plasma in the patient and will only have action at the site of application. Furthermore, intravenous administration will unlikely be taken at a frequency that will be relevant for the slow and progressive nature of AD. Therefore, oral administration was selected as the administration route of interest and a data-frame containing only supplements administered orally was created. Then patient IDs were extracted from this data frame for each of the Supplement of interest.

Route<-c("po","PO","P.O.","P.O","p.o","p.o.","6","oral","Oral","ORAL","orally")
Meds$route<-ifelse(Meds$CMROUTE %in% Route,1,0)
Meds<-Meds[Meds$route==1,]

#### Data Cleaning

This code turns all taken supplements and medicines in upper case factors.

Meds$CMMED<-as.factor(toupper(Meds$CMMED))
Meds$CMMEDO<-as.factor(toupper(Meds$CMMEDO))
length(levels(Meds$CMMED))

## [1] 3673

#levels(Meds$CMMED)[400:700]

#### Data Cleaning Zinc

This code identifies all participants who are taking zinc supplements.

zinc<-c("ZINC")
Meds$zinc<-numeric(length(Meds$ID))
Meds$zinc[Meds$CMMED %in% zinc | Meds$CMMEDO %in% zinc] <- 1
zincIDS<-Meds[Meds$zinc==1,3]
zincIDS<-unique(zincIDS)
length(zincIDS)

## [1] 62

supplementsrow<-c()
for(i in 1:1){
supplementsrow<-append(supplementsrow,grep(zinc[i],Meds$CMMED ))
supplementsrow<-append(supplementsrow,grep(zinc[i],Meds$CMMEDO ))
}

zincIDS<-Meds[supplementsrow,3]
zincIDS<-sort(unique(zincIDS))
Meds$zinc<-c()
Meds$zinc<-(Meds$RID %in% zincIDS)*Meds$CMCONT
zincIDS<-unique(Meds[Meds$zinc==1,3])
length(zincIDS)

## [1] 99

#### Data Cleaning Multivitamin

This code identifies all participants who are taking multivitamin supplements.

multivit<-c("MUITIVITAMIN","MULTIVIT","MULIT-VITAMIN","MULITVITAMIN","MULT. VIT","MULTI-VATMIN","MULTI-VITAMIN","MULTI ITAMIN","MULTI VITAMINE","MULTI VIT","MULTIVIAMIN","MULTI VITS","MULTIPLE VITAMINS","MULTIVITIAMIN","MULTIVITAMIS","MULTIVITS","MULTIVITAMIS","MULTIVITAMIN","MULTIPLE VITAMIN","CENTRUM","MVI", "ONE A DAY", "ONE-A-DAY","MINERAL", "MINERALS")
Meds$multivit<-numeric(length(Meds$ID))
Meds$multivit[Meds$CMMED %in% multivit | Meds$CMMEDO %in% multivit] <- 1
multivitIDS<-Meds[Meds$multivit==1,3]
multivitIDS<-unique(multivitIDS)
length(multivit)

## [1] 25

supplementsrow<-c()
for(i in 1:25){
supplementsrow<-append(supplementsrow,grep(multivit[i],Meds$CMMED ))
supplementsrow<-append(supplementsrow,grep(multivit[i],Meds$CMMEDO ))
}

multivitIDS<-Meds[supplementsrow,3]
multivitIDS<-sort(unique(multivitIDS))
length(multivitIDS)

## [1] 1431

multivitIDS<-unique(Meds[Meds$multivit==1,3])
length(multivitIDS)

## [1] 745

#### Data Cleaning Selenium

This code identifies all participants who are taking selenium supplements.

Selenium<-c("SELENIUM")
Meds$Selenium<-numeric(length(Meds$ID))
Meds$Selenium[Meds$CMMED %in% Selenium | Meds$CMMEDO %in% Selenium] <- 1
SeleniumIDS<-Meds[Meds$Selenium==1,3]
SeleniumIDS<-unique(SeleniumIDS)
length(SeleniumIDS)

## [1] 57

length(Selenium)

## [1] 1

supplementsrow<-c()
for(i in 1:1){
supplementsrow<-append(supplementsrow,grep(Selenium[1],Meds$CMMED ))
supplementsrow<-append(supplementsrow,grep(Selenium[1],Meds$CMMEDO ))
}

SeleniumIDS<-Meds[supplementsrow,3]
SeleniumIDS<-sort(unique(SeleniumIDS))


Meds$Selenium<-c()
Meds$Selenium<-(Meds$RID %in% SeleniumIDS)*Meds$CMCONT
SeleniumIDS<-unique(Meds[Meds$Selenium==1,3])
length(SeleniumIDS)

## [1] 58

#### Data Cleaning Iron

This code identifies all participants who are taking iron supplements.

iron<-c("IRON")
Meds$iron<-numeric(length(Meds$ID))
Meds$iron[Meds$CMMED %in% iron | Meds$CMMEDO %in% iron] <- 1
ironIDS<-Meds[Meds$iron==1,3]
ironIDS<-unique(ironIDS)
length(ironIDS)

## [1] 97

length(iron)

## [1] 1

supplementsrow<-c()
for(i in 1:1){
supplementsrow<-append(supplementsrow,grep(iron[i],Meds$CMMED ))
supplementsrow<-append(supplementsrow,grep(iron[i],Meds$CMMEDO ))
}

ironIDS<-Meds[supplementsrow,3]
ironIDS<-sort(unique(ironIDS))


Meds$iron<-c()
Meds$iron<-(Meds$RID %in% ironIDS)*Meds$CMCONT
ironIDS<-unique(Meds[Meds$iron==1,3])
length(ironIDS)

## [1] 147

#### Data Cleaning Magnesium

This code identifies all participants who are taking magnesium supplements.

magnese<-c("MAGNESIUM","MAGNESUIM","CAL-MAG-ZINC","CAL MAG","CAL MAG T", "MG")
Meds$magnese<-numeric(length(Meds$ID))
Meds$magnese[Meds$CMMED %in% magnese | Meds$CMMEDO %in% magnese] <- 1
magneseIDS<-Meds[Meds$magnese==1,3]
magneseIDS<-unique(magneseIDS)
length(magneseIDS)

## [1] 135

length(magnese)

## [1] 6

supplementsrow<-c()
for(i in 1:6){
supplementsrow<-append(supplementsrow,grep(magnese[i],Meds$CMMED ))
supplementsrow<-append(supplementsrow,grep(magnese[i],Meds$CMMEDO ))
}

magneseIDS<-Meds[supplementsrow,3]
magneseIDS<-sort(unique(magneseIDS))


Meds$magnese<-c()
Meds$magnese<-(Meds$RID %in% magneseIDS)*Meds$CMCONT
magneseIDS<-unique(Meds[Meds$magnese==1,3])
length(magneseIDS)

## [1] 244

#### Data Cleaning Calcium

This code identifies all participants who are taking calcium supplements.

calcium<-c("CALCIUM",
"CALCITRATE",
"CALCITRIOL",
"CALCITROL",
"CA + MG + K",
"CA 500D VITAMIN SUPPLEMENT",
"CA W/ VITAMIN D",
"CA, MG, K",
"CACIUM +D",
"CAL-MAG-ZINC",
"CAL MAG",
"CAL MAG T",
"CALCARB + VITAMIN D",
"CALTRATE")
Meds$calcium<-numeric(length(Meds$ID))
Meds$calcium[Meds$CMMED %in% calcium | Meds$CMMEDO %in% calcium] <- 1
calciumIDS<-Meds[Meds$calcium==1,3]
calciumIDS<-unique(calciumIDS)
length(calciumIDS)

## [1] 432

supplementsrow<-c()
for(i in 1:14){
supplementsrow<-append(supplementsrow,grep(calcium[i],Meds$CMMED ))
supplementsrow<-append(supplementsrow,grep(calcium[i],Meds$CMMEDO ))
}

calciumIDS<-Meds[supplementsrow,3]
calciumIDS<-sort(unique(calciumIDS))


Meds$calcium<-c()
Meds$calcium<-(Meds$RID %in% calciumIDS)*Meds$CMCONT
calciumIDS<-unique(Meds[Meds$calcium==1,3])
length(calciumIDS)

## [1] 872

### Combing supplements data frame with outcome variable data frame

The ADNIMERGE dataset is a complied data set of key variables from the ADNI study including information on cognitive decline and confounding variables. Information about Supplement supplements use was merged with the ADNIMERGE dataset.

summary<-read.csv("C:/Users/jrivers/Dropbox/Science/Projects/Epidemiology ADNI/Analysis/ADNIMERGEnew.csv", header=T)
Finalsupplements<-summary[,which(names(summary) %in% c("RID","DX_bl","AGE","PTGENDER","PTEDUCAT","APOE4","CDRSB","ADAS11","ADAS13","MMSE","Hippocampus","WholeBrain","CDRSB_bl","ADAS11_bl","ADAS13_bl","MMSE_bl","M"))]
Finalsupplements$zinc<-(Finalsupplements$RID %in% zincIDS)*1
Finalsupplements$Selenium<-(Finalsupplements$RID %in% SeleniumIDS)*1
Finalsupplements$iron<-(Finalsupplements$RID %in% ironIDS)*1
Finalsupplements$multi<-(Finalsupplements$RID %in% multivitIDS)*1
Finalsupplements$calcium<-(Finalsupplements$RID %in% calciumIDS)*1
Finalsupplements$magnesium<-(Finalsupplements$RID %in% magneseIDS)*1
Finalsupplements$x<-Finalsupplements$zinc+Finalsupplements$Selenium+Finalsupplements$iron+Finalsupplements$multi+Finalsupplements$calcium+Finalsupplements$magnesium
Finalsupplements$Supplement<-1*(Finalsupplements$x>0)
Finalsupplements$No.Supplement<-(Finalsupplements$Supplement-1)^2
str(Finalsupplements)

## 'data.frame': 13278 obs. of 26 variables:
## $ RID : int 2 3 3 3 3 4 4 4 4 4 ...
## $ DX_bl : Factor w/ 6 levels "","AD","CN","EMCI",..: 3 2 2 2 2 5 5 5 5 5 ...
## $ AGE : num 74.3 81.3 81.3 81.3 81.3 67.5 67.5 67.5 67.5 67.5 ...
## $ PTGENDER : Factor w/ 2 levels "Female","Male": 2 2 2 2 2 2 2 2 2 2 ...
## $ PTEDUCAT : int 16 18 18 18 18 10 10 10 10 10 ...
## $ APOE4 : int 0 1 1 1 1 0 0 0 0 0 ...
## $ CDRSB : num 0 4.5 6 3.5 8 1 0.5 1 1 1 ...
## $ ADAS11 : num 10.7 22 19 24 25.7 ...
## $ ADAS13 : num 18.7 31 30 35 37.7 ...
## $ MMSE : int 28 20 24 17 19 27 28 26 27 25 ...
## $ Hippocampus : int 8336 5319 5446 5157 5139 6869 6439 6451 6373 6213 ...
## $ WholeBrain : int 1229740 1129830 1100060 1095640 1088560 1154980 1116280 1117390 1095210 1085350 ...
## $ CDRSB_bl : num 0 4.5 4.5 4.5 4.5 1 1 1 1 1 ...
## $ ADAS11_bl : num 10.7 22 22 22 22 ...
## $ ADAS13_bl : num 18.7 31 31 31 31 ...
## $ MMSE_bl : int 28 20 20 20 20 27 27 27 27 27 ...
## $ M : int 0 0 6 12 24 0 6 12 18 36 ...
## $ zinc : num 0 0 0 0 0 0 0 0 0 0 ...
## $ Selenium : num 0 0 0 0 0 0 0 0 0 0 ...
## $ iron : num 0 0 0 0 0 0 0 0 0 0 ...
## $ multi : num 1 1 1 1 1 0 0 0 0 0 ...
## $ calcium : num 0 0 0 0 0 0 0 0 0 0 ...
## $ magnesium : num 0 0 0 0 0 0 0 0 0 0 ...
## $ x : num 1 1 1 1 1 0 0 0 0 0 ...
## $ Supplement : num 1 1 1 1 1 0 0 0 0 0 ...
## $ No.Supplement: num 0 0 0 0 0 1 1 1 1 1 ...

### Searching for confounding pre-existing condition

The RECMHIST is a dataset of pre-existing condition. Diabetes and vascular diseases have been linked to changes in AD incidence and progression. Additionally, arthritis and headaches were the leading indications of supplement use. Therefore, patient IDs where extracted and merged with the cognitive scoring dataset to include these variables in the analyses.

#### Diabetes

This code identifies all participants who have been diagnosed with diabetes.

medhist<-read.csv("C:/Users/jrivers/Dropbox/Science/Projects/Epidemiology ADNI/Analysis/RECMHISTnew.csv", header=T)
diseases<-data.frame(table(medhist$MHDESC))
diabetes<-c("Disbetes", "diabetes","Diabetes", "DIABETES", "diabetic","Diabetic","DIABETIC","diabetes", "diabetic","DIABETIC","DIABETES")

diseaserow<-c()
for(i in 1:11){
 diseaserow<-append(diseaserow,grep(diabetes[i],medhist$MHDESC ))
 diseaserow<-append(diseaserow,grep(diabetes[i],medhist$MHDESC ))
}

diabetesIDS<-medhist[diseaserow,3]
diabetesIDS<-sort(unique(diabetesIDS))


medhist$diabetes<-c()
medhist$diabetes<-(medhist$RID %in% diabetesIDS)*1
diabetesIDS<-unique(medhist[medhist$diabetes==1,3])
length(diabetesIDS)

## [1] 234

#### Cardiovascular

This code identifies all participants who have been diagnosed with cardiovascular related diseases including high cholesterol, high blood pressure, heart disease and stroke.

cardiovasc<-c("cholest", "pressure","hypertension", "Cholest", "Pressure","Hypertension","stroke","Stroke","Heart attack","heart attack","Hyoertension", "blood pressure", "cholesterol", "Cholesterol", "PRESSURE", "hypercholesterolemia","Hypercholesterolemia","pressure","CHOLESTEROL")

diseaserow<-c()
for(i in 1:19){
 diseaserow<-append(diseaserow,grep(cardiovasc[i],medhist$MHDESC ))
 diseaserow<-append(diseaserow,grep(cardiovasc[i],medhist$MHDESC ))
}

cardiovascIDS<-medhist[diseaserow,3]
cardiovascIDS<-sort(unique(cardiovascIDS))


medhist$cardiovasc<-c()
medhist$cardiovasc<-(medhist$RID %in% cardiovascIDS)*1
cardiovascIDS<-unique(medhist[medhist$cardiovasc==1,3])
length(cardiovascIDS)

## [1] 1441

#### Arthritis

This code identifies all participants who have been diagnosed with arthritis.

arthrit<-c("Arthritis", "arthritis","ARTHRITIS", "arthritic", "Arthritic","Arthitis","osteoarthritis","Osteoarthritis", "OSTEOARTHRITIS","ARTHRITIC","Arthritric")

diseaserow<-c()
for(i in 1:11){
 diseaserow<-append(diseaserow,grep(arthrit[i],medhist$MHDESC ))
}

arthritIDS<-medhist[diseaserow,3]
arthritIDS<-sort(unique(arthritIDS))


medhist$arthrit<-c()
medhist$arthrit<-(medhist$RID %in% arthritIDS)*1
arthritIDS<-unique(medhist[medhist$arthrit==1,3])
length(arthritIDS)

## [1] 896

#### Migraine headache

This code identifies all participants who have been diagnosed with migraines or headaches.

headache<-c("headache", "Headache","HEADACHE", "Migraine", "migraine","MIGRAINE")


diseaserow<-c()
for(i in 1:11){
 diseaserow<-append(diseaserow,grep(headache[i],medhist$MHDESC ))
}

headacheIDS<-medhist[diseaserow,3]
headacheIDS<-sort(unique(headacheIDS))


medhist$headache<-c()
medhist$headache<-(medhist$RID %in% headacheIDS)*1
headacheIDS<-unique(medhist[medhist$headache==1,3])
length(headacheIDS)

## [1] 205

#### Smoker

This code identifies all participants who smoke cigarettes.

smoke<-c("Smoker", "smoker","SMOKER", "SMOKE", "Smoke","smoke","Smoking","smoking","SMOKING")

diseaserow<-c()
for(i in 1:9){
 diseaserow<-append(diseaserow,grep(smoke[i],medhist$MHDESC ))
}

smokeIDS<-medhist[diseaserow,3]
smokeIDS<-sort(unique(smokeIDS))


medhist$smoke<-c()
medhist$smoke<-(medhist$RID %in% smokeIDS)*1
smokeIDS<-unique(medhist[medhist$smoke==1,3])
length(smokeIDS)

## [1] 579

### Final supplements table with disease summary

This code collates the disease and comorbidity table.

Finalsupplements$diab<-(Finalsupplements$RID %in% diabetesIDS)*1
Finalsupplements$arthrit<-(Finalsupplements$RID %in% arthritIDS)*1
Finalsupplements$vasc<-(Finalsupplements$RID %in% cardiovascIDS)*1
Finalsupplements$smoke<-(Finalsupplements$RID %in% smokeIDS)*1
Finalsupplements$headache<-(Finalsupplements$RID %in% headacheIDS)*1

### Renaming columns and data cleaning

This code cleans the final data table, renaming column names to be more recognisable.

names(Finalsupplements)[1]<-"ID"
names(Finalsupplements)[2]<-"diagn"
names(Finalsupplements)[4]<-"Gender"
names(Finalsupplements)[5]<-"Yrs.edu"
names(Finalsupplements)[11]<-"Hippo"
names(Finalsupplements)[12]<-"Brain"
names(Finalsupplements)[13]<-"CDRSB.bl"
names(Finalsupplements)[14]<-"ADAS11.bl"
names(Finalsupplements)[15]<-"ADAS13.bl"
names(Finalsupplements)[16]<-"MMSE.bl"
names(Finalsupplements)[17]<-"M"
Finalsupplements$diagn<-as.character(Finalsupplements$diagn)
Finalsupplements$diagn[Finalsupplements$diagn=="CN"]<-"1CN"
Finalsupplements$diagn[Finalsupplements$diagn=="EMCI"]<-"2EMCI"
Finalsupplements$diagn[Finalsupplements$diagn=="LMCI"]<-"3LMCI"
Finalsupplements$diagn[Finalsupplements$diagn=="AD"]<-"4AD"
Finalsupplements<-Finalsupplements[Finalsupplements$diagn!="SMC",]
Finalsupplements$diagn<-as.factor(Finalsupplements$diagn)

### Centering data

Future analyses required the centering of data to aid convergence of the model by adjusting the explanatory variables to comparable values.

Finalsupplements$Yrs.ed.Z<-(Finalsupplements$Yrs.edu-mean(Finalsupplements$Yrs.edu))/sd(Finalsupplements$Yrs.edu)
Finalsupplements$AGE.Z<-(Finalsupplements$AGE-mean(Finalsupplements$AGE))/sd(Finalsupplements$AGE)
Finalsupplements$M.Z<-(Finalsupplements$M-mean(Finalsupplements$M))/sd(Finalsupplements$M)
Finalsupplements$AGEraw<-(Finalsupplements$AGE)
Finalsupplements$AGE<-(Finalsupplements$AGE-mean(Finalsupplements$AGE))
write.csv(Finalsupplements, file="C:/Users/jrivers/Dropbox/Science/Projects/Epidemiology ADNI/Zinc/Finalzinc.csv")

### Removing missing data from explanatory variables

This code removes missing values from the data table, ensures the data is recorded properly and stores the new data table.

Fulldata<-read.csv("C:/Users/jrivers/Dropbox/Science/Projects/Epidemiology ADNI/Zinc/Finalzinc.csv", header=T)

data<-Fulldata[!is.na(Fulldata$APOE4),]
data<-data[!is.na(data$Gender),]
data<-data[!is.na(data$AGE),]
data<-data[!is.na(data$Yrs.edu),]
data<-data[!is.na(data$diagn),]
data$APOE4<-as.factor(data$APOE4)
data$ID<-as.factor(data$ID)
data$edu.cat[data$Yrs.edu<13]<-"4early"
data$edu.cat[data$Yrs.edu>=13 & data$Yrs.edu<16]<-"3mid"
data$edu.cat[data$Yrs.edu>=16& data$Yrs.edu<18]<-"2tertiary"
data$edu.cat[data$Yrs.edu>=18]<-"1post"
data$neg.b.MMSE<-30-data$MMSE
fulldata<-data
write.csv(data, file="C:/Users/jrivers/Dropbox/Science/Projects/Epidemiology ADNI/Zinc/CleanedFinalDataZinc2.csv")

## Constructing table of participants at the beginning of the study

### Variables tabled by diagnosis

This code constructs the baseline statistics table for the study.

initialdata<-fulldata[fulldata$M==0,]
str(initialdata)

## 'data.frame': 1619 obs. of 38 variables:
## $ X : int 1 2 6 11 16 21 23 27 32 35 ...
## $ ID : Factor w/ 1619 levels "2","3","4","5",..: 1 2 3 4 5 6 8 9 10 11 ...
## $ diagn : Factor w/ 5 levels "","1CN","2EMCI",..: 2 5 4 2 4 5 5 2 2 2 ...
## $ AGE : num 0.5094 7.5094 -6.2906 -0.0906 6.6094 ...
## $ Gender : Factor w/ 2 levels "Female","Male": 2 2 2 2 1 2 1 1 2 2 ...
## $ Yrs.edu : int 16 18 10 16 13 10 12 12 18 9 ...
## $ APOE4 : Factor w/ 3 levels "0","1","2": 1 2 1 1 1 2 2 1 2 2 ...
## $ CDRSB : num 0 4.5 1 0 0.5 6 5 0 0 0 ...
## $ ADAS11 : num 10.67 22 14.33 8.67 18.67 ...
## $ ADAS13 : num 18.7 31 21.3 14.7 25.7 ...
## $ MMSE : int 28 20 27 29 25 20 24 29 29 28 ...
## $ Hippo : int 8336 5319 6869 7075 5348 6729 5485 6730 6732 7309 ...
## $ Brain : int 1229740 1129830 1154980 1116630 927510 875798 1033540 861749 942730 936539 ...
## $ CDRSB.bl : num 0 4.5 1 0 0.5 6 5 0 0 0 ...
## $ ADAS11.bl : num 10.67 22 14.33 8.67 18.67 ...
## $ ADAS13.bl : num 18.7 31 21.3 14.7 25.7 ...
## $ MMSE.bl : int 28 20 27 29 25 20 24 29 29 28 ...
## $ M : int 0 0 0 0 0 0 0 0 0 0 ...
## $ zinc : int 0 0 0 1 0 0 0 0 0 0 ...
## $ Selenium : int 0 0 0 0 0 0 0 0 0 0 ...
## $ iron : int 0 0 0 0 1 0 0 0 0 0 ...
## $ multi : int 1 1 0 1 0 0 0 0 1 0 ...
## $ calcium : int 0 0 0 1 1 0 1 1 1 0 ...
## $ magnesium : int 0 0 0 0 0 0 0 0 0 0 ...
## $ x : int 1 1 0 3 2 0 1 1 2 0 ...
## $ Supplement : int 1 1 0 1 1 0 1 1 1 0 ...
## $ No.Supplement: int 0 0 1 0 0 1 0 0 0 1 ...
## $ diab : int 1 0 0 0 0 1 0 0 1 0 ...
## $ arthrit : int 1 1 1 0 0 0 0 0 0 0 ...
## $ vasc : int 0 1 1 0 1 1 0 0 1 1 ...
## $ smoke : int 0 0 1 1 0 0 0 0 0 0 ...
## $ headache : int 0 0 0 0 0 0 0 0 0 0 ...
## $ Yrs.ed.Z : num 0.00513 0.71259 -2.11726 0.00513 -1.05607 ...
## $ AGE.Z : num 0.0727 1.0718 -0.8978 -0.0129 0.9433 ...
## $ M.Z : num -0.991 -0.991 -0.991 -0.991 -0.991 ...
## $ AGEraw : num 74.3 81.3 67.5 73.7 80.4 75.4 73.9 78.5 80.8 65.4 ...
## $ edu.cat : chr "2tertiary" "1post" "4early" "2tertiary" ...
## $ neg.b.MMSE : num 2 10 3 1 5 10 6 1 1 2 ...

Particpants<-length(initialdata$ID)

Gender<-table(initialdata$diagn, by=initialdata$Gender)

Mean.Age<-round(c(0,mean(initialdata$AGE[initialdata$diagn=="1CN"]),mean(initialdata$AGE[initialdata$diagn=="2EMCI"]),mean(initialdata$AGE[initialdata$diagn=="3LMCI"]),mean(initialdata$AGE[initialdata$diagn=="4AD"])),2)
SD.AGE<-round(c(0,sd(initialdata$AGE[initialdata$diagn=="1CN"]),sd(initialdata$AGE[initialdata$diagn=="2EMCI"]),sd(initialdata$AGE[initialdata$diagn=="3LMCI"]),sd(initialdata$AGE[initialdata$diagn=="4AD"])),2)
Mean.Age.total<-round(mean(initialdata$AGE,2))
SD.AGE.total<-round(sd(initialdata$AGE),2)

calcium<-table(initialdata$diagn,by=initialdata$calcium)[,2]
Selenium<-table(initialdata$diagn,by=initialdata$Selenium)[,2]
Iron<-table(initialdata$diagn,by=initialdata$iron)[,2]
Magnesium<-table(initialdata$diagn,by=initialdata$magnesium)[,2]
MultiVitamin<-table(initialdata$diagn,by=initialdata$multi)[,2]
Zinc<-table(initialdata$diagn,by=initialdata$zinc)[,2]
No.Supplement<-table(initialdata$diagn,by=initialdata$No.Supplement)[,2]

Cardiovascular.Pathology<-table(initialdata$diagn,by=initialdata$vasc)[,2]
Diabetes<-table(initialdata$diagn,by=initialdata$diab)[,2]
Smoker<-table(initialdata$diagn,by=initialdata$smoke)[,2]
Headache<-table(initialdata$diagn,by=initialdata$headache)[,2]
Arthritis<-table(initialdata$diagn,by=initialdata$arthrit)[,2]

APOE4.1<-table(initialdata$diagn,by=initialdata$APOE4)[,2]
APOE4.2<-table(initialdata$diagn,by=initialdata$APOE4)[,3]
Early.Education<-table(initialdata$diagn,by=initialdata$edu.cat)[,4]
Mid.Education<-table(initialdata$diagn,by=initialdata$edu.cat)[,3]
Tertiary.Education<-table(initialdata$diagn,by=initialdata$edu.cat)[,2]
Postgraduate.Education<-table(initialdata$diagn,by=initialdata$edu.cat)[,1]


Mean.MMSE<-round(c(0,mean(initialdata$MMSE[initialdata$diagn=="1CN"],na.rm=TRUE),mean(initialdata$MMSE[initialdata$diagn=="2EMCI"],na.rm=TRUE),mean(initialdata$MMSE[initialdata$diagn=="3LMCI"],na.rm=TRUE),mean(initialdata$MMSE[initialdata$diagn=="4AD"],na.rm=TRUE)),2)
SD.MMSE<-round(c(0,sd(initialdata$MMSE[initialdata$diagn=="1CN"],na.rm=TRUE),sd(initialdata$MMSE[initialdata$diagn=="2EMCI"],na.rm=TRUE),sd(initialdata$MMSE[initialdata$diagn=="3LMCI"],na.rm=TRUE),sd(initialdata$MMSE[initialdata$diagn=="4AD"],na.rm=TRUE)),2)

Mean.MMSE.total<-round(mean(initialdata$MMSE,2))
SD.MMSE.total<-round(sd(initialdata$MMSE),2)

Mean.ADAS13<-round(c(0,mean(initialdata$ADAS13[initialdata$diagn=="1CN"],na.rm=TRUE),mean(initialdata$ADAS13[initialdata$diagn=="2EMCI"],na.rm=TRUE),mean(initialdata$ADAS13[initialdata$diagn=="3LMCI"],na.rm=TRUE),mean(initialdata$ADAS13[initialdata$diagn=="4AD"],na.rm=TRUE)),2)

SD.ADAS13<-round(c(0,sd(initialdata$ADAS13[initialdata$diagn=="1CN"],na.rm=TRUE),sd(initialdata$ADAS13[initialdata$diagn=="2EMCI"],na.rm=TRUE),sd(initialdata$ADAS13[initialdata$diagn=="3LMCI"],na.rm=TRUE),sd(initialdata$ADAS13[initialdata$diagn=="4AD"],na.rm=TRUE)),2)

Mean.ADAS.total<-round(mean(initialdata$ADAS13,na.rm=TRUE),2)
SD.ADAS.total<-round(sd(initialdata$ADAS13,na.rm=TRUE),2)

table<-t(cbind(Gender, Mean.Age, SD.AGE,calcium,Selenium,Iron,Magnesium,MultiVitamin,Zinc,No.Supplement,Cardiovascular.Pathology,Diabetes,Smoker, Arthritis, Headache, APOE4.1,APOE4.2,Early.Education,Mid.Education,Tertiary.Education,Postgraduate.Education, Mean.MMSE,SD.MMSE,Mean.ADAS13,SD.ADAS13))[,2:5]
table

## 1CN 2EMCI 3LMCI 4AD
## Female 206.00 136.00 219.00 149.00
## Male 209.00 170.00 345.00 185.00
## Mean.Age 1.00 -2.54 0.23 1.15
## SD.AGE 5.73 7.40 7.50 7.82
## calcium 185.00 116.00 199.00 89.00
## Selenium 17.00 3.00 18.00 5.00
## Iron 35.00 29.00 37.00 10.00
## Magnesium 60.00 32.00 54.00 16.00
## MultiVitamin 161.00 70.00 199.00 94.00
## Zinc 20.00 15.00 26.00 4.00
## No.Supplement 131.00 129.00 219.00 176.00
## Cardiovascular.Pathology 271.00 182.00 348.00 195.00
## Diabetes 37.00 37.00 48.00 28.00
## Smoker 109.00 69.00 150.00 84.00
## Arthritis 183.00 126.00 208.00 109.00
## Headache 36.00 30.00 49.00 23.00
## APOE4.1 103.00 110.00 234.00 157.00
## APOE4.2 11.00 21.00 73.00 65.00
## Early.Education 44.00 45.00 102.00 82.00
## Mid.Education 89.00 74.00 88.00 68.00
## Tertiary.Education 120.00 76.00 167.00 100.00
## Postgraduate.Education 162.00 111.00 207.00 84.00
## Mean.MMSE 29.07 28.33 27.18 23.18
## SD.MMSE 1.12 1.57 1.81 2.06
## Mean.ADAS13 9.33 12.63 18.69 29.96
## SD.ADAS13 4.33 5.40 6.52 8.05

t(cbind(Mean.Age.total,SD.AGE.total,Mean.MMSE.total,SD.MMSE.total,Mean.ADAS.total,SD.ADAS.total))

## [,1]
## Mean.Age.total 0.00
## SD.AGE.total 7.25
## Mean.MMSE.total 28.00
## SD.MMSE.total 2.69
## Mean.ADAS.total 17.41
## SD.ADAS.total 9.59

### Age of education level

This code tables educations status by age to ensure these variables are colinear.

mean.AGEraw<-round(c(mean(initialdata$AGEraw[initialdata$edu.cat=="1post"],na.rm=TRUE),mean(initialdata$AGEraw[initialdata$edu.cat=="2tertiary"],na.rm=TRUE),mean(initialdata$AGEraw[initialdata$edu.cat=="3mid"],na.rm=TRUE),mean(initialdata$AGEraw[initialdata$edu.cat=="4early"],na.rm=TRUE)),2)

sd.AGEraw<-round(c(sd(initialdata$AGEraw[initialdata$edu.cat=="1post"],na.rm=TRUE),sd(initialdata$AGEraw[initialdata$edu.cat=="2tertiary"],na.rm=TRUE),sd(initialdata$AGEraw[initialdata$edu.cat=="3mid"],na.rm=TRUE),sd(initialdata$AGEraw[initialdata$edu.cat=="4early"],na.rm=TRUE)),2)

Education<-c("Post-graduate","Tertiary","Mid", "Early")

data.frame(Education, mean.AGEraw, sd.AGEraw)

## Education mean.AGEraw sd.AGEraw
## 1 Post-graduate 73.31 7.31
## 2 Tertiary 73.94 7.19
## 3 Mid 73.56 7.53
## 4 Early 75.35 6.74

### Tabling supplements by dependent variables

#### MMSE

This code creates the baseline statistics for the MMSE cognitive score by supplement use.

calcium<-mean(initialdata$MMSE.bl[initialdata$calcium==1])
calcium.sd<-sd(initialdata$MMSE.bl[initialdata$calcium==1])
Selenium<-mean(initialdata$MMSE.bl[initialdata$Selenium==1])
Selenium.sd<-sd(initialdata$MMSE.bl[initialdata$Selenium==1])
iron<-mean(initialdata$MMSE.bl[initialdata$iron==1])
iron.sd<-sd(initialdata$MMSE.bl[initialdata$iron==1])
multi<-mean(initialdata$MMSE.bl[initialdata$multi==1])
multi.sd<-sd(initialdata$MMSE.bl[initialdata$multi==1])
magnesium<-mean(initialdata$MMSE.bl[initialdata$magnesium==1])
magnesium.sd<-sd(initialdata$MMSE.bl[initialdata$magnesium==1])
zinc<-mean(initialdata$MMSE.bl[initialdata$zinc==1])
zinc.sd<-sd(initialdata$MMSE.bl[initialdata$zinc==1])

No.Supplement<-mean(initialdata$MMSE.bl[initialdata$No.Supplement==1])
No.Supplement.sd<-sd(initialdata$MMSE.bl[initialdata$No.Supplement==1])

MMSE.table<-data.frame(calcium,calcium.sd,Selenium,Selenium.sd,iron,iron.sd,multi,multi.sd,magnesium,magnesium.sd,zinc,zinc.sd,No.Supplement,No.Supplement.sd)

MMSE.table

## calcium calcium.sd Selenium Selenium.sd iron iron.sd multi multi.sd
## 1 27.49915 2.486443 28.09302 2.635056 27.4955 1.994875 27.21183 2.617593
## magnesium magnesium.sd zinc zinc.sd No.Supplement No.Supplement.sd
## 1 27.88889 2.18123 27.81538 2.05314 26.66107 2.84319

lm0<-glm.nb(round(30-MMSE.bl)~1,data=initialdata)

lm1<-glm.nb(round(30-MMSE.bl)~calcium+ Selenium+iron+multi+magnesium+zinc,data=initialdata)
summary(lm1)

##
## Call:
## glm.nb(formula = round(30 - MMSE.bl) ~ calcium + Selenium + iron +
## multi + magnesium + zinc, data = initialdata, init.theta = 1.716737536,
## link = log)
##
## Deviance Residuals:
## Min 1Q Median 3Q Max
## -1.9202 -0.9739 -0.2947 0.4819 2.4851
##
## Coefficients:
## Estimate Std. Error z value Pr(>|z|)
## (Intercept) 1.19624 0.03338 35.833 < 2e-16 ***
## calcium -0.19197 0.05214 -3.682 0.000232 ***
## Selenium -0.34769 0.16583 -2.097 0.036030 *
## iron -0.11837 0.09770 -1.212 0.225680
## multi -0.04194 0.05206 -0.806 0.420530
## magnesium -0.24896 0.08984 -2.771 0.005585 **
## zinc -0.08338 0.13672 -0.610 0.541942
## ---
## Signif. codes: 0 '***' 0.001 '**' 0.01 '*' 0.05 '.' 0.1 ' ' 1
##
## (Dispersion parameter for Negative Binomial(1.7167) family taken to be 1)
##
## Null deviance: 1893.7 on 1618 degrees of freedom
## Residual deviance: 1852.1 on 1612 degrees of freedom
## AIC: 7134.4
##
## Number of Fisher Scoring iterations: 1
##
##
## Theta: 1.717
## Std. Err.: 0.108
##
## 2 x log-likelihood: -7118.389

par(mfrow=c(2,2)); plot(lm1)


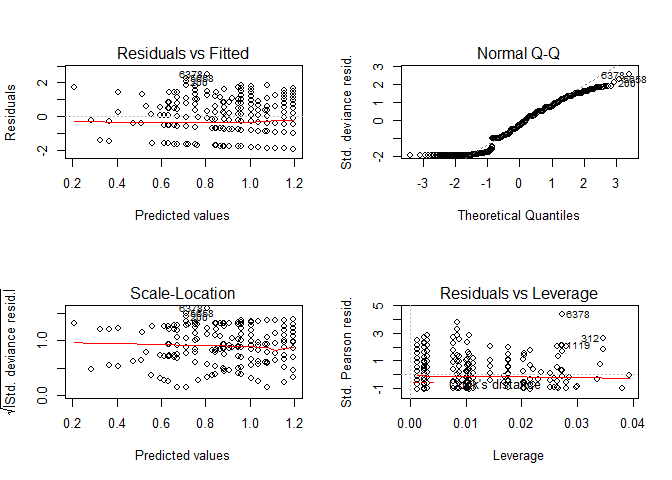


This figure demonstrates that there is no relationship between the residuals and fitted values. Indicating the validity of the model. Normality of the residuals is not expected in the logistic model.

anova(lm0,lm1)

## Likelihood ratio tests of Negative Binomial Models
##
## Response: round(30 - MMSE.bl)
## Model theta Resid. df
## 1 1 1.634106 1618
## 2 calcium + Selenium + iron + multi + magnesium + zinc 1.716738 1612
## 2 x log-lik. Test df LR stat. Pr(Chi)
## 1 -7159.351
## 2 -7118.389 1 vs 2 6 40.96231 2.945592e-07

calcium.lm<-glm.nb(round(30-MMSE.bl)~ Selenium+iron+multi+magnesium+zinc,data=initialdata)
Selenium.lm<-glm.nb(round(30-MMSE.bl)~calcium+ iron+multi+magnesium+zinc,data=initialdata)
iron.lm<-glm.nb(round(30-MMSE.bl)~calcium+ Selenium+multi+magnesium+zinc,data=initialdata)
multi.lm<-glm.nb(round(30-MMSE.bl)~calcium+ Selenium+iron+magnesium+zinc,data=initialdata)
magnesium.lm<-glm.nb(round(30-MMSE.bl)~calcium+ Selenium+iron+multi+zinc,data=initialdata)
zinc.lm<-glm.nb(round(30-MMSE.bl)~calcium+ Selenium+iron+multi+magnesium,data=initialdata)

supplements<-c("calcium","Selenium","iron","multi","magnesium","Zinc")
table<-cbind(supplements,rbind(anova(calcium.lm,lm1)[2,6:8],
anova(Selenium.lm,lm1)[2,6:8],
anova(iron.lm,lm1)[2,6:8],
anova(multi.lm,lm1)[2,6:8],
anova(magnesium.lm,lm1)[2,6:8],
anova(zinc.lm,lm1)[2,6:8]))
Adjusted.P.Value<-p.adjust(table[,4],method="bonferroni")
table<-cbind(table,Adjusted.P.Value)
table

## supplements df LR stat. Pr(Chi) Adjusted.P.Value
## 2 calcium 1 13.2560432 0.0002717025 0.001630215
## 21 Selenium 1 4.3596413 0.0368000524 0.220800314
## 22 iron 1 1.4413228 0.2299253986 1.000000000
## 23 multi 1 0.6509230 0.4197828831 1.000000000
## 24 magnesium 1 7.3803540 0.0065940188 0.039564113
## 25 Zinc 1 0.3658387 0.5452813360 1.000000000

#### Diagnosis

This code creates a table of cognitive diagnosis by supplement. This indicates that no supplement use was associated with higher prevalence of Alzheimer’s diseae.

calcium<-table(initialdata$diagn,by=initialdata$calcium)[,2]
Selenium<-table(initialdata$diagn,by=initialdata$Selenium)[,2]
iron<-table(initialdata$diagn,by=initialdata$iron)[,2]
multi<-table(initialdata$diagn,by=initialdata$multi)[,2]
magnesium<-table(initialdata$diagn,by=initialdata$magnesium)[,2]
zinc<-table(initialdata$diagn,by=initialdata$zinc)[,2]
No.Supplement<-table(initialdata$diagn,by=initialdata$No.Supplement)[,2]

Diagnosis.table<-data.frame(t(cbind(calcium,Selenium,iron,multi,magnesium,zinc,No.Supplement)))
Diagnosis.table<-Diagnosis.table[,2:5]
names(Diagnosis.table)<-c("CN","EMCI","LMCI","AD")
Diagnosis.table$CN.percent<-Diagnosis.table[,1]/(Diagnosis.table[,2]+Diagnosis.table[,3]+Diagnosis.table[,4]+Diagnosis.table[,1])*100

Diagnosis.table$EMCI.percent<-Diagnosis.table[,2]/(Diagnosis.table[,2]+Diagnosis.table[,3]+Diagnosis.table[,4]+Diagnosis.table[,1])*100

Diagnosis.table$LMCI.percent<-Diagnosis.table[,3]/(Diagnosis.table[,2]+Diagnosis.table[,3]+Diagnosis.table[,4]+Diagnosis.table[,1])*100

Diagnosis.table$AD.percent<-Diagnosis.table[,4]/(Diagnosis.table[,2]+Diagnosis.table[,3]+Diagnosis.table[,4]+Diagnosis.table[,1])*100

Diagnosis.table$total<-(Diagnosis.table[,5]+Diagnosis.table[,3]+Diagnosis.table[,4]+Diagnosis.table[,1])

Diagnosis.table

## CN EMCI LMCI AD CN.percent EMCI.percent LMCI.percent AD.percent
## calcium 185 116 199 89 31.40917 19.694397 33.78608 15.110357
## Selenium 17 3 18 5 39.53488 6.976744 41.86047 11.627907
## iron 35 29 37 10 31.53153 26.126126 33.33333 9.009009
## multi 161 70 199 94 30.72519 13.358779 37.97710 17.938931
## magnesium 60 32 54 16 37.03704 19.753086 33.33333 9.876543
## zinc 20 15 26 4 30.76923 23.076923 40.00000 6.153846
## No.Supplement 131 129 219 176 20.00000 19.694656 33.43511 26.870229
## total
## calcium 504.40917
## Selenium 79.53488
## iron 113.53153
## multi 484.72519
## magnesium 167.03704
## zinc 80.76923
## No.Supplement 546.00000

chisq<-chisq.test(Diagnosis.table[,1:4])


chisq

##
## Pearson's Chi-squared test
##
## data: Diagnosis.table[, 1:4]
## X-squared = 92.461, df = 18, p-value = 5.206e-12

contrib<-100*chisq$residuals^2/chisq$statistic
contrib

## CN EMCI LMCI AD
## calcium 2.1191768 0.6429226 0.26518429 3.61093794
## Selenium 2.0571406 3.2719374 0.62678503 1.14078313
## iron 0.4318355 3.9756137 0.09450069 5.69391984
## multi 1.1389076 7.6516748 1.44215438 0.04827222
## magnesium 4.6777993 0.1924188 0.13791992 6.83576554
## zinc 0.1465392 0.8625156 0.50364536 5.68863482
## No.Supplement 17.3822021 0.7152373 0.49134132 28.15423438

res1 <- cor.mtest(contrib, conf.level = 0.95)

col2 <- colorRampPalette(c("#67001F", "#B2182B", "#D6604D", "#F4A582",
 "#FDDBC7", "#FFFFFF", "#D1E5F0", "#92C5DE",
 "#4393C3", "#2166AC", "#053061"))


pearson<-chisq$residuals

#pdf("Risidual.#pdf", width=16/2.54, heigh=12/2.54 , useDingbats=F)
corrplot(contrib, p.mat = res1$p, insig = "blank",pch.col=1,is.cor=F,tl.col=1, cl.pos="b", number.digits=2,number.cex=0.5,cl.cex=1,cl.length=5, cl.lim=c(0,50), col=rev(col2(200)))


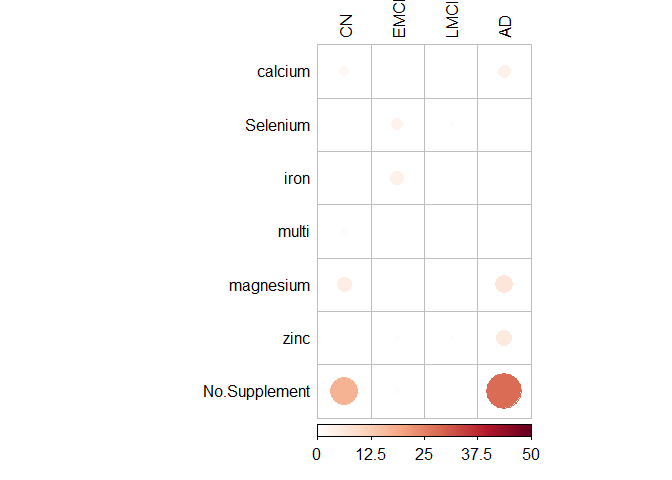


This figure demonstrates that no supplement use has the highest impact of the chi-squared statistic and thus p value.

#dev.off()

#pdf("Percentage.#pdf", width=16/2.54, heigh=12/2.54, useDingbats=F)

corrplot(pearson, p.mat = res1$p, insig = "blank",pch.col=1,is.cor=F,tl.col=1, cl.pos="b", number.digits=2,number.cex=0.5,cl.cex=1,cl.length=5, cl.lim=c(-8,8),col=rev(col2(200)))


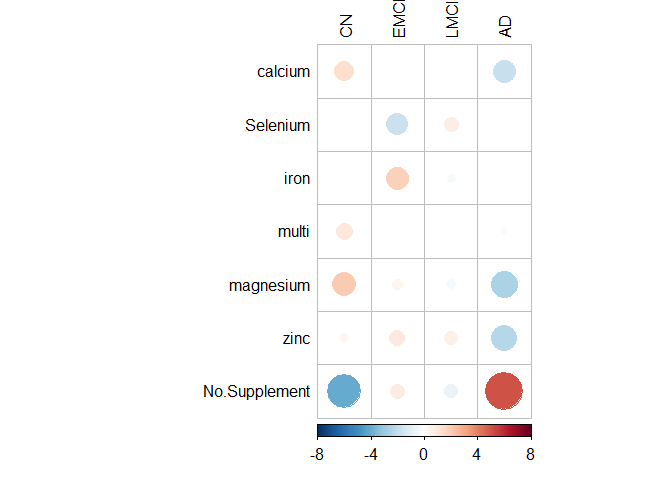


This figure demonstrates the directional association with Alzheimer’s disease prevalence. No supplement use was associated with elevated risk, while zinc and magnesium use was associated with low Alzheimer’s disease prevalence.

#dev.off()

Diagnosis.table$Supplement<-rownames(Diagnosis.table)

melt.diag<-melt(Diagnosis.table,id="Supplement")
percent<-c("CN.percent","EMCI.percent","LMCI.percent","AD.percent")
melt.diag<-melt.diag[(melt.diag$variable%in%percent),]

positions<-c("No.Supplement","calcium","Selenium","iron","multi","magnesium","zinc")

#pdf("Proportions.#pdf", width=16/2.54, heigh=12/2.54, useDingbats=F)
qplot(x=Supplement,y=value,fill=variable,data=melt.diag,geom="col")+ theme(axis.text.x = element_text(size=10, angle=60, hjust=1), plot.background=element_rect(0),panel.background=element_rect(0),axis.line=element_line(1, size=1))+
 scale_y_continuous(expand = c(0, 0), breaks=seq(0, 100,by=20),limits=c(0,100))+
 scale_fill_manual(values=c("#7cafe2","#4b912299","#e4630066","#f78a8a"))+
 scale_x_discrete(limits = positions)


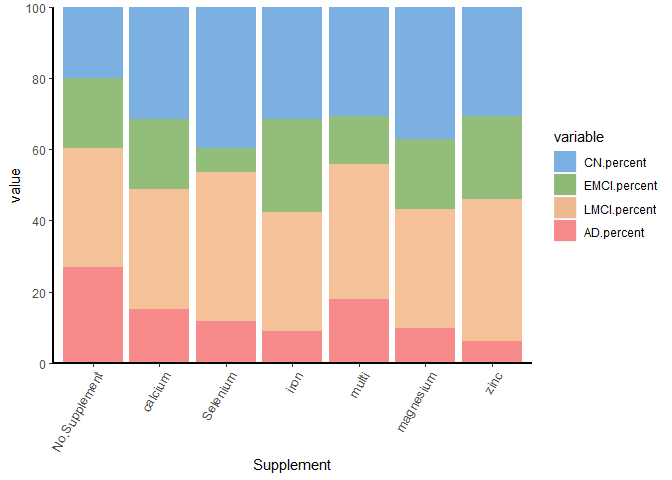


This figure demonstrates the directional association with Alzheimer’s disease prevalence. No supplement use was associated with elevated risk, while zinc, iron and magnesium use was associated with low Alzheimer’s disease prevalence.

#dev.off()

##### Logistic regression of diagnosis by supplement

This code performs logistic regressions on Alzheimer’s disease prevalence among supplement user types. Adjusting for significant nuisance variables.

initialdata$AD<-(initialdata$diagn=="4AD")*1
colnames(initialdata)

## [1] "X" "ID" "diagn" "AGE"
## [5] "Gender" "Yrs.edu" "APOE4" "CDRSB"
## [9] "ADAS11" "ADAS13" "MMSE" "Hippo"
## [13] "Brain" "CDRSB.bl" "ADAS11.bl" "ADAS13.bl"
## [17] "MMSE.bl" "M" "zinc" "Selenium"
## [21] "iron" "multi" "calcium" "magnesium"
## [25] "x" "Supplement" "No.Supplement" "diab"
## [29] "arthrit" "vasc" "smoke" "headache"
## [33] "Yrs.ed.Z" "AGE.Z" "M.Z" "AGEraw"
## [37] "edu.cat" "neg.b.MMSE" "AD"

baselog <- glm(AD ~ AGE + Gender + APOE4 + diab + arthrit + vasc + smoke + headache + edu.cat, data = initialdata , family = binomial(link = "logit"))

calciumlog <- glm(AD ~ AGE + Gender + APOE4 + diab + arthrit + vasc + smoke + headache + edu.cat + calcium , data = initialdata , family = binomial(link = "logit"))

ironlog<-glm(AD ~ AGE + Gender + APOE4 + diab + arthrit + vasc + smoke + headache + edu.cat + iron , data = initialdata , family = binomial(link = "logit"))

magnesiumlog<-glm(AD ~ AGE + Gender + APOE4 + diab + arthrit + vasc + smoke + headache + edu.cat + magnesium, data = initialdata , family = binomial(link = "logit"))

multilog<-glm(AD ~ AGE + Gender + APOE4 + diab + arthrit + vasc + smoke + headache + edu.cat + multi, data = initialdata , family = binomial(link = "logit"))

seleniumlog<-glm(AD ~ AGE + Gender + APOE4 + diab + arthrit + vasc + smoke + headache + edu.cat + Selenium, data = initialdata , family = binomial(link = "logit"))

zinclog <- glm(AD ~ AGE + Gender + APOE4 + diab + arthrit + vasc + smoke + headache + edu.cat + zinc , data = initialdata , family = binomial(link = "logit"))


calcium.prevalence<-c(anova(baselog, calciumlog, test="Chisq")[2,3:5],summary(calciumlog)$coefficients[14,1])

iron.prevalence<-c(anova(baselog, ironlog, test="Chisq")[2,3:5],summary(ironlog)$coefficients[14,1])

magnesium.prevalence<-c(anova(baselog, magnesiumlog, test="Chisq")[2,3:5],summary(magnesiumlog)$coefficients[14,1])

multi.prevalence<-c(anova(baselog, multilog, test="Chisq")[2,3:5],summary(multilog)$coefficients[14,1])

selenium.prevalence<-c(anova(baselog, seleniumlog, test="Chisq")[2,3:5],summary(seleniumlog)$coefficients[14,1])

zinc.prevalence<-c(anova(baselog, zinclog, test="Chisq")[2,3:5],summary(zinclog)$coefficients[14,1])

Logistic.table<-rbind(calcium.prevalence,iron.prevalence,magnesium.prevalence,multi.prevalence,selenium.prevalence,zinc.prevalence)

colnames(Logistic.table)<-c("DF", "Deviance", "P-Value", "Coefficient")

Logistic.table

## DF Deviance P-Value Coefficient
## calcium.prevalence 1 18.34742 1.840685e-05 -0.6303473
## iron.prevalence 1 9.383195 0.002189835 -0.9549798
## magnesium.prevalence 1 12.95828 0.0003185089 -0.9096091
## multi.prevalence 1 1.858948 0.1727463 -0.1914683
## selenium.prevalence 1 1.5908 0.2072119 -0.5866441
## zinc.prevalence 1 8.582941 0.003393268 -1.294433

#### APOE

This code investigates the prevalence of APOE4 genotype among supplement user types.

calcium<-table(initialdata$APOE4,by=initialdata$calcium)[,2]
Selenium<-table(initialdata$APOE4,by=initialdata$Selenium)[,2]
iron<-table(initialdata$APOE4,by=initialdata$iron)[,2]
multi<-table(initialdata$APOE4,by=initialdata$multi)[,2]
magnesium<-table(initialdata$APOE4,by=initialdata$magnesium)[,2]
zinc<-table(initialdata$APOE4,by=initialdata$zinc)[,2]
No.Supplement<-table(initialdata$APOE4,by=initialdata$No.Supplement)[,2]


APOE4.table<-data.frame(t(cbind(calcium,Selenium,iron,multi,magnesium,zinc,No.Supplement)))
names(APOE4.table)<-c("WT","HET","HOMO")
APOE4.table$WT.Percent<-APOE4.table[,1]/(APOE4.table[,2]+APOE4.table[,3]+APOE4.table[,1])*100
APOE4.table$HET.Percent<-APOE4.table[,2]/(APOE4.table[,2]+APOE4.table[,3]+APOE4.table[,1])*100
APOE4.table$HOMO.Percent<-APOE4.table[,3]/(APOE4.table[,2]+APOE4.table[,3]+APOE4.table[,1])*100

APOE4.table

## WT HET HOMO WT.Percent HET.Percent HOMO.Percent
## calcium 321 209 59 54.49915 35.48387 10.016978
## Selenium 23 16 4 53.48837 37.20930 9.302326
## iron 68 32 11 61.26126 28.82883 9.909910
## multi 292 185 47 55.72519 35.30534 8.969466
## magnesium 86 62 14 53.08642 38.27160 8.641975
## zinc 37 25 3 56.92308 38.46154 4.615385
## No.Supplement 315 261 79 48.09160 39.84733 12.061069

APOE4.table.chisq<-chisq.test(APOE4.table[,1:3])

## Warning in chisq.test(APOE4.table[, 1:3]): Chi-squared approximation may be
## incorrect

APOE4.table.chisq

##
## Pearson's Chi-squared test
##
## data: APOE4.table[, 1:3]
## X-squared = 15.344, df = 12, p-value = 0.2232

#### Education

This code investigates the education status among supplement user types. Indicating supplement use is equal across the education spectrum.

calcium<-table(initialdata$edu.cat,by=initialdata$calcium)[,2]
Selenium<-table(initialdata$edu.cat,by=initialdata$Selenium)[,2]
iron<-table(initialdata$edu.cat,by=initialdata$iron)[,2]
multi<-table(initialdata$edu.cat,by=initialdata$multi)[,2]
magnesium<-table(initialdata$edu.cat,by=initialdata$magnesium)[,2]
zinc<-table(initialdata$edu.cat,by=initialdata$zinc)[,2]
No.Supplement<-table(initialdata$edu.cat,by=initialdata$No.Supplement)[,2]

Education.table<-data.frame(t(cbind(calcium,Selenium,iron,multi,magnesium,zinc,No.Supplement)))
names(Education.table)<-c("Primary","Secondary","Tertiary","Post-grad")
Education.table$Primary.percent<-Education.table[,1]/(Education.table[,2]+Education.table[,3]+Education.table[,4]+Education.table[,1])*100
Education.table$Secondary.percent<-Education.table[,2]/(Education.table[,2]+Education.table[,3]+Education.table[,4]+Education.table[,1])*100
Education.table$Tertiary.percent<-Education.table[,3]/(Education.table[,2]+Education.table[,1]+Education.table[,3]+Education.table[,4])*100
Education.table$Postgrad.percent<-Education.table[,4]/(Education.table[,2]+Education.table[,1]+Education.table[,3]+Education.table[,4])*100
Education.table

## Primary Secondary Tertiary Post-grad Primary.percent
## calcium 200 155 136 98 33.95586
## Selenium 26 10 4 3 60.46512
## iron 37 26 30 18 33.33333
## multi 193 151 102 78 36.83206
## magnesium 57 48 30 27 35.18519
## zinc 22 24 10 9 33.84615
## No.Supplement 223 197 115 120 34.04580
## Secondary.percent Tertiary.percent Postgrad.percent
## calcium 26.31579 23.089983 16.638370
## Selenium 23.25581 9.302326 6.976744
## iron 23.42342 27.027027 16.216216
## multi 28.81679 19.465649 14.885496
## magnesium 29.62963 18.518519 16.666667
## zinc 36.92308 15.384615 13.846154
## No.Supplement 30.07634 17.557252 18.320611

Education.table.chisq<-chisq.test(Education.table[,1:4])
Education.table.chisq

##
## Pearson's Chi-squared test
##
## data: Education.table[, 1:4]
## X-squared = 29.295, df = 18, p-value = 0.04489

#### Gender

This code investigates the gender proportions among supplement user types.

calcium<-table(initialdata$Gender,by=initialdata$calcium)[,2]
Selenium<-table(initialdata$Gender,by=initialdata$Selenium)[,2]
iron<-table(initialdata$Gender,by=initialdata$iron)[,2]
multi<-table(initialdata$Gender,by=initialdata$multi)[,2]
magnesium<-table(initialdata$Gender,by=initialdata$magnesium)[,2]
zinc<-table(initialdata$Gender,by=initialdata$zinc)[,2]
No.Supplement<-table(initialdata$Gender,by=initialdata$No.Supplement)[,2]


Gender.table<-data.frame(t(cbind(calcium,Selenium,iron,multi,magnesium,zinc,No.Supplement)))
names(Gender.table)<-c("Male","Female")
Gender.table$Male.Percent<-Gender.table[,1]/(Gender.table[,2]+Gender.table[,1])*100
Gender.table$Female.Percent<-Gender.table[,2]/(Gender.table[,2]+Gender.table[,1])*100


Gender.table

## Male Female Male.Percent Female.Percent
## calcium 385 204 65.36503 34.63497
## Selenium 14 29 32.55814 67.44186
## iron 56 55 50.45045 49.54955
## multi 200 324 38.16794 61.83206
## magnesium 91 71 56.17284 43.82716
## zinc 25 40 38.46154 61.53846
## No.Supplement 232 423 35.41985 64.58015

Gender.table.chisq<-chisq.test(Gender.table[,1:2])
Gender.table.chisq

##
## Pearson's Chi-squared test
##
## data: Gender.table[, 1:2]
## X-squared = 142.95, df = 6, p-value < 2.2e-16

contrib<-100*Gender.table.chisq$residuals^2/Gender.table.chisq$statistic
contrib

## Male Female
## calcium 30.8448859 26.9960040
## Selenium 1.2839936 1.1237745
## iron 0.2374102 0.2077857
## multi 5.6809655 4.9720841
## magnesium 2.1913336 1.9178949
## zinc 0.6568857 0.5749183
## No.Supplement 12.4316544 10.8804096

res1 <- cor.mtest(contrib, conf.level = 0.95)

col2 <- colorRampPalette(c("#67001F", "#B2182B", "#D6604D", "#F4A582",
 "#FDDBC7", "#FFFFFF", "#D1E5F0", "#92C5DE",
 "#4393C3", "#2166AC", "#053061"))


pearson<-Gender.table.chisq$residuals


corrplot(contrib, p.mat = res1$p, insig = "blank",pch.col=1,is.cor=F,tl.col=1, cl.pos="b", number.digits=2,number.cex=0.5,cl.cex=1,cl.length=3, cl.lim=c(0,50), col=rev(col2(200)))


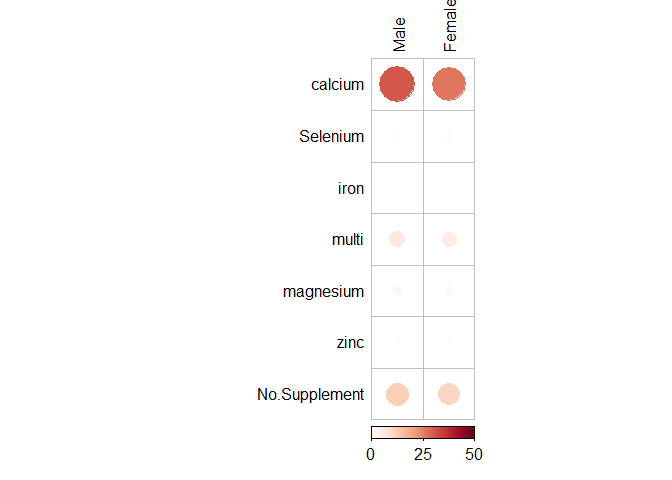


This figure demonstrates what supplement user groups have the most impact on the significant difference in gender proportions across these groups.

corrplot(pearson, p.mat = res1$p, insig = "blank",pch.col=1,is.cor=F,tl.col=1, cl.pos="b", number.digits=2,number.cex=0.5,cl.cex=1,cl.length=3, cl.lim=c(-8,8),col=rev(col2(200)))


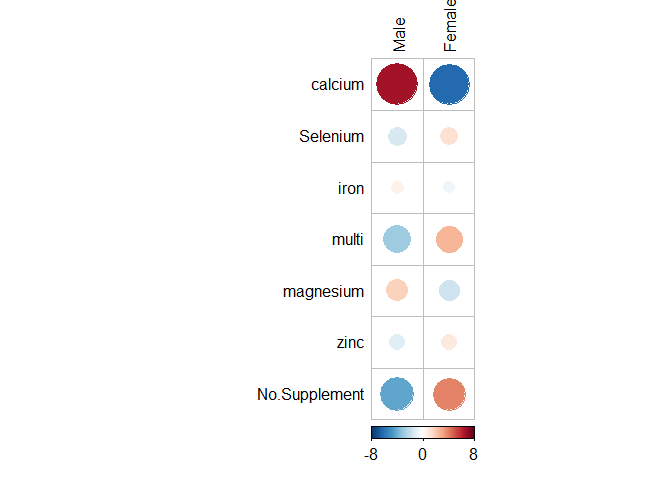


This figure demonstrates the directional relationship between supplement use and gender proportions. Indicating the males are more likely to take calcium supplements and while females are more likely to take no supplements.

#### AGE

This code investigates mean age differences across supplement user groups.

calcium<-mean(initialdata$AGEraw[initialdata$calcium==1])
calcium.sd<-sd(initialdata$AGEraw[initialdata$calcium==1])
Selenium<-mean(initialdata$AGEraw[initialdata$Selenium==1])
Selenium.sd<-sd(initialdata$AGEraw[initialdata$Selenium==1])
iron<-mean(initialdata$AGEraw[initialdata$iron==1])
iron.sd<-sd(initialdata$AGEraw[initialdata$iron==1])
multi<-mean(initialdata$AGEraw[initialdata$multi==1])
multi.sd<-sd(initialdata$AGEraw[initialdata$multi==1])
magnesium<-mean(initialdata$AGEraw[initialdata$magnesium==1])
magnesium.sd<-sd(initialdata$AGEraw[initialdata$magnesium==1])
zinc<-mean(initialdata$AGEraw[initialdata$zinc==1])
zinc.sd<-sd(initialdata$AGEraw[initialdata$zinc==1])

No.Supplement<-mean(initialdata$AGEraw[initialdata$No.Supplement==1])
No.Supplement.sd<-sd(initialdata$AGEraw[initialdata$No.Supplement==1])

AGE.table<-data.frame(calcium,calcium.sd,Selenium,Selenium.sd,iron,iron.sd,multi,multi.sd,magnesium,magnesium.sd,zinc,zinc.sd,No.Supplement,No.Supplement.sd)

AGE.table

## calcium calcium.sd Selenium Selenium.sd iron iron.sd multi multi.sd
## 1 73.80951 7.005713 74.48837 6.182048 74.27387 7.077053 74.44847 7.103045
## magnesium magnesium.sd zinc zinc.sd No.Supplement No.Supplement.sd
## 1 72.91605 6.681047 73.41077 7.095578 73.67084 7.534876

lm0<-lm(AGEraw~1,data=initialdata)
lm1<-lm(AGEraw~calcium+ Selenium+iron+multi+magnesium+zinc,data=initialdata)
summary(lm1)

##
## Call:
## lm(formula = AGEraw ~ calcium + Selenium + iron + multi + magnesium +
## zinc, data = initialdata)
##
## Residuals:
## Min 1Q Median 3Q Max
## -19.4502 -4.4234 0.0856 5.0856 17.6856
##
## Coefficients:
## Estimate Std. Error t value Pr(>|t|)
## (Intercept) 73.71440 0.25515 288.902 <2e-16 ***
## calcium -0.09186 0.38829 -0.237 0.8130
## Selenium 0.64447 1.14290 0.564 0.5729
## iron 0.40612 0.71672 0.567 0.5710
## multi 0.83575 0.38981 2.144 0.0322 *
## magnesium -1.09013 0.64281 -1.696 0.0901 .
## zinc -0.12808 0.97665 -0.131 0.8957
## ---
## Signif. codes: 0 '***' 0.001 '**' 0.01 '*' 0.05 '.' 0.1 ' ' 1
##
## Residual standard error: 7.247 on 1612 degrees of freedom
## Multiple R-squared: 0.005414, Adjusted R-squared: 0.001712
## F-statistic: 1.463 on 6 and 1612 DF, p-value: 0.1874

anova(lm0,lm1)

## Analysis of Variance Table
##
## Model 1: AGEraw ~ 1
## Model 2: AGEraw ~ calcium + Selenium + iron + multi + magnesium + zinc
## Res.Df RSS Df Sum of Sq F Pr(>F)
## 1 1618 85132
## 2 1612 84671 6 460.92 1.4625 0.1874

par(mfrow=c(2,2)); plot(lm1)


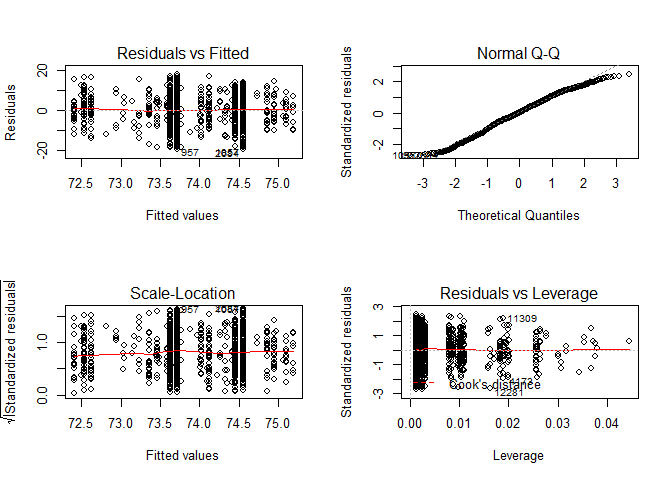


This figure demonstrates that there is no relationship between the residuals and fitted values. Indicating the validity of the model. The residuals were approximately normal, within the tolerances of the model given the large total sample size.

calcium.lm1<-lm(AGEraw~ Selenium+iron+multi+magnesium+zinc,data=initialdata)
Selenium.lm1<-lm(AGEraw~calcium+ iron+multi+magnesium+zinc,data=initialdata)
iron.lm1<-lm(AGEraw~calcium+ Selenium+multi+magnesium+zinc,data=initialdata)
multi.lm1<-lm(AGEraw~calcium+ Selenium+iron+magnesium+zinc,data=initialdata)
magnesium.lm1<-lm(AGEraw~calcium+ Selenium+iron+multi+zinc,data=initialdata)
zinc.lm1<-lm(AGEraw~calcium+ Selenium+iron+multi+magnesium,data=initialdata)

supplements<-c("calcium","Selenium","iron","multi","magnesium","Zinc")
table<-cbind(supplements,rbind(anova(calcium.lm1,lm1)[2,5:6],
anova(Selenium.lm1,lm1)[2,5:6],
anova(iron.lm1,lm1)[2,5:6],
anova(multi.lm1,lm1)[2,5:6],
anova(magnesium.lm1,lm1)[2,5:6],
anova(zinc.lm1,lm1)[2,5:6]))
Adjusted.P.Value<-p.adjust(table[,3],method="bonferroni")
table<-cbind(table,Adjusted.P.Value)
table

## supplements F Pr(>F) Adjusted.P.Value
## 2 calcium 0.05597282 0.81300824 1.0000000
## 21 Selenium 0.31797836 0.57290356 1.0000000
## 22 iron 0.32107555 0.57104088 1.0000000
## 23 multi 4.59662506 0.03218415 0.1931049
## 24 magnesium 2.87605726 0.09009900 0.5405940
## 25 Zinc 0.01719964 0.89567507 1.0000000

#### Smoking status

This code investigates the prevalence of smoker across supplement user groups.

calcium<-table(initialdata$smoke,by=initialdata$calcium)[,2]
Selenium<-table(initialdata$smoke,by=initialdata$Selenium)[,2]
iron<-table(initialdata$smoke,by=initialdata$iron)[,2]
multi<-table(initialdata$smoke,by=initialdata$multi)[,2]
magnesium<-table(initialdata$smoke,by=initialdata$magnesium)[,2]
zinc<-table(initialdata$smoke,by=initialdata$zinc)[,2]
No.Supplement<-table(initialdata$smoke,by=initialdata$No.Supplement)[,2]

Smoking.table<-data.frame(t(cbind(calcium,Selenium,iron,multi,magnesium,zinc,No.Supplement)))
names(Smoking.table)<-c("Non-smoker","Smoker")
Smoking.table$percent<-Smoking.table[,2]/(Smoking.table[,2]+Smoking.table[,1])*100
Smoking.table

## Non-smoker Smoker percent
## calcium 449 140 23.76910
## Selenium 32 11 25.58140
## iron 81 30 27.02703
## multi 395 129 24.61832
## magnesium 118 44 27.16049
## zinc 54 11 16.92308
## No.Supplement 479 176 26.87023

chisq.test(Smoking.table[,1:2])

##
## Pearson's Chi-squared test
##
## data: Smoking.table[, 1:2]
## X-squared = 4.5981, df = 6, p-value = 0.5963

#### Arthritis

This code investigates the prevalence of arthritis across supplement user groups.

calcium<-table(initialdata$arthrit,by=initialdata$calcium)[,2]
Selenium<-table(initialdata$arthrit,by=initialdata$Selenium)[,2]
iron<-table(initialdata$arthrit,by=initialdata$iron)[,2]
multi<-table(initialdata$arthrit,by=initialdata$multi)[,2]
magnesium<-table(initialdata$arthrit,by=initialdata$magnesium)[,2]
zinc<-table(initialdata$arthrit,by=initialdata$zinc)[,2]
No.Supplement<-table(initialdata$arthrit,by=initialdata$No.Supplement)[,2]

Arthritis.table<-data.frame(t(cbind(calcium,Selenium,iron,multi,magnesium,zinc,No.Supplement)))
names(Arthritis.table)<-c("Non-Arthritis","Arthritis")
Arthritis.table$percent<-Arthritis.table[,2]/(Arthritis.table[,2]+Arthritis.table[,1])*100
Arthritis.table

## Non-Arthritis Arthritis percent
## calcium 328 261 44.31239
## Selenium 24 19 44.18605
## iron 54 57 51.35135
## multi 298 226 43.12977
## magnesium 92 70 43.20988
## zinc 34 31 47.69231
## No.Supplement 442 213 32.51908

chisq.test(Arthritis.table[,1:2])

##
## Pearson's Chi-squared test
##
## data: Arthritis.table[, 1:2]
## X-squared = 29.768, df = 6, p-value = 4.351e-05

chisq<-chisq.test(Arthritis.table[,1:2])


chisq

##
## Pearson's Chi-squared test
##
## data: Arthritis.table[, 1:2]
## X-squared = 29.768, df = 6, p-value = 4.351e-05

contrib<-100*chisq$residuals^2/chisq$statistic
contrib

## Non-Arthritis Arthritis
## calcium 4.1013456 5.9485879
## Selenium 0.2782078 0.4035124
## iron 7.0007325 10.1538561
## multi 1.6008226 2.3218316
## magnesium 0.5296762 0.7682419
## zinc 1.7475255 2.5346094
## No.Supplement 25.5513687 37.0596818

res1 <- cor.mtest(contrib, conf.level = 0.95)

col2 <- colorRampPalette(c("#67001F", "#B2182B", "#D6604D", "#F4A582",
 "#FDDBC7", "#FFFFFF", "#D1E5F0", "#92C5DE",
 "#4393C3", "#2166AC", "#053061"))


pearson<-chisq$residuals


corrplot(contrib, p.mat = res1$p, insig = "blank",pch.col=1,is.cor=F,tl.col=1, cl.pos="b", number.digits=2,number.cex=0.5,cl.cex=1,cl.length=3, cl.lim=c(0,50), col=rev(col2(200)))


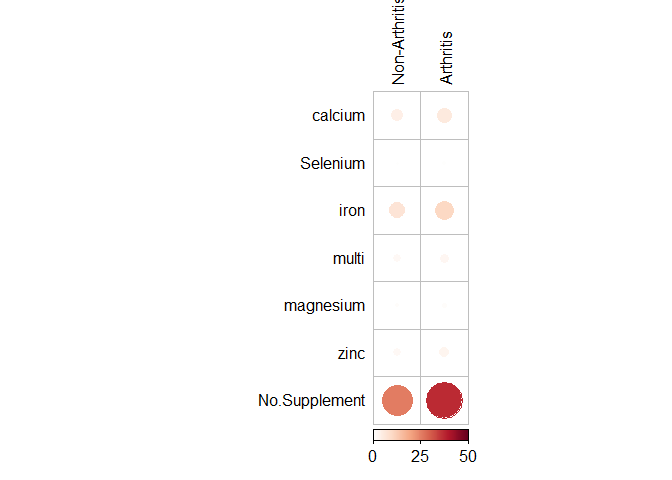


This figure demonstrates which supplement groups have the largest impact on the significance in the arthritic prevalence about these groups.

corrplot(pearson, p.mat = res1$p, insig = "blank",pch.col=1,is.cor=F,tl.col=1, cl.pos="b", number.digits=2,number.cex=0.5,cl.cex=1,cl.length=3, cl.lim=c(-6,6),col=rev(col2(200)))


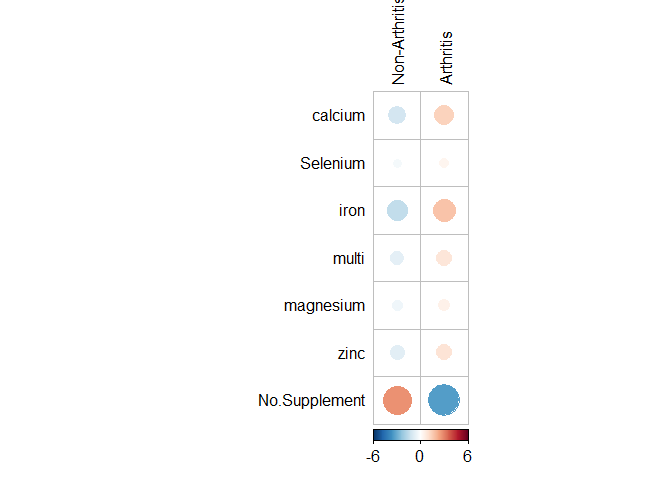


This figure demonstrates the directional relationship between supplement use and arthritis prevalence. No supplement use was associated with a lower prevalence of arthritis.

#### Headache

This code investigates the association between headache prevalence and supplement use.

calcium<-table(initialdata$headache,by=initialdata$calcium)[,2]
Selenium<-table(initialdata$headache,by=initialdata$Selenium)[,2]
iron<-table(initialdata$headache,by=initialdata$iron)[,2]
multi<-table(initialdata$headache,by=initialdata$multi)[,2]
magnesium<-table(initialdata$headache,by=initialdata$magnesium)[,2]
zinc<-table(initialdata$headache,by=initialdata$zinc)[,2]
No.Supplement<-table(initialdata$headache,by=initialdata$No.Supplement)[,2]


Headache.table<-data.frame(t(cbind(calcium,Selenium,iron,multi,magnesium,zinc,No.Supplement)))
names(Headache.table)<-c("Non-Headache","Headache")
Headache.table$percent<-Headache.table[,2]/(Headache.table[,2]+Headache.table[,1])*100
Headache.table

## Non-Headache Headache percent
## calcium 524 65 11.035654
## Selenium 38 5 11.627907
## iron 101 10 9.009009
## multi 476 48 9.160305
## magnesium 148 14 8.641975
## zinc 61 4 6.153846
## No.Supplement 613 42 6.412214

chisq.test(Headache.table[,1:2])

## Warning in chisq.test(Headache.table[, 1:2]): Chi-squared approximation may be
## incorrect

##
## Pearson's Chi-squared test
##
## data: Headache.table[, 1:2]
## X-squared = 9.4559, df = 6, p-value = 0.1495

chisq<-chisq.test(Headache.table[,1:2])

## Warning in chisq.test(Headache.table[, 1:2]): Chi-squared approximation may be
## incorrect

chisq

##
## Pearson's Chi-squared test
##
## data: Headache.table[, 1:2]
## X-squared = 9.4559, df = 6, p-value = 0.1495

contrib<-100*chisq$residuals^2/chisq$statistic
contrib

## Non-Headache Headache
## calcium 3.571535174 37.25415147
## Selenium 0.413241928 4.31046501
## iron 0.008746632 0.09123482
## multi 0.103106907 1.07549279
## magnesium 0.002120662 0.02212031
## zinc 0.507044536 5.28890604
## No.Supplement 4.142459162 43.20937456

res1 <- cor.mtest(contrib, conf.level = 0.95)

col2 <- colorRampPalette(c("#67001F", "#B2182B", "#D6604D", "#F4A582",
 "#FDDBC7", "#FFFFFF", "#D1E5F0", "#92C5DE",
 "#4393C3", "#2166AC", "#053061"))


pearson<-chisq$residuals


corrplot(contrib, p.mat = res1$p, insig = "blank",pch.col=1,is.cor=F,tl.col=1, cl.pos="b", number.digits=2,number.cex=0.5,cl.cex=1,cl.length=3, cl.lim=c(0,60), col=rev(col2(200)))


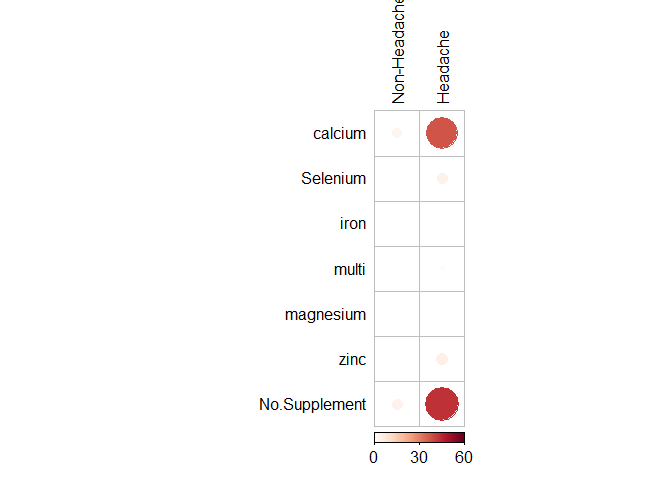


This figure demonstrates which supplement user groups have the largest impact on the significance differences in headache prevalence.

corrplot(pearson, p.mat = res1$p, insig = "blank",pch.col=1,is.cor=F,tl.col=1, cl.pos="b", number.digits=2,number.cex=0.5,cl.cex=1,cl.length=3, cl.lim=c(-5,5),col=rev(col2(200)))


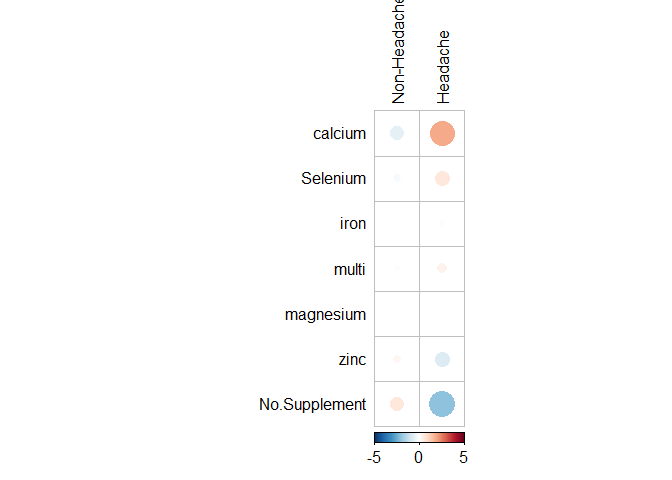


This code demonstrates the directional association between supplement user groups and headache prevalence. No supplement use was associated with the lowest headache prevalence while calcium use was associated with elevated headache prevalence.

#### Cardiovascular disease

This code investigates the association between supplement use and cardiovascular disease.

calcium<-table(initialdata$vasc,by=initialdata$calcium)[,2]
Selenium<-table(initialdata$vasc,by=initialdata$Selenium)[,2]
iron<-table(initialdata$vasc,by=initialdata$iron)[,2]
multi<-table(initialdata$vasc,by=initialdata$multi)[,2]
magnesium<-table(initialdata$vasc,by=initialdata$magnesium)[,2]
zinc<-table(initialdata$vasc,by=initialdata$zinc)[,2]
No.Supplement<-table(initialdata$vasc,by=initialdata$No.Supplement)[,2]

Cardiovascular.Pathology.Table<-data.frame(t(cbind(calcium,Selenium,iron,multi,magnesium,zinc,No.Supplement)))
names(Cardiovascular.Pathology.Table)<-c("Non-cardiovascular","Cardiovascular")
Cardiovascular.Pathology.Table$percent<-Cardiovascular.Pathology.Table[,2]/(Cardiovascular.Pathology.Table[,2]+Cardiovascular.Pathology.Table[,1])*100
Cardiovascular.Pathology.Table

## Non-cardiovascular Cardiovascular percent
## calcium 243 346 58.74363
## Selenium 18 25 58.13953
## iron 41 70 63.06306
## multi 202 322 61.45038
## magnesium 71 91 56.17284
## zinc 25 40 61.53846
## No.Supplement 242 413 63.05344

chisq<-chisq.test(Cardiovascular.Pathology.Table[,1:2])

chisq

##
## Pearson's Chi-squared test
##
## data: Cardiovascular.Pathology.Table[, 1:2]
## X-squared = 4.3704, df = 6, p-value = 0.6267

contrib<-100*chisq$residuals^2/chisq$statistic
contrib

## Non-cardiovascular Cardiovascular
## calcium 14.8150452 9.5441990
## Selenium 1.8028684 1.1614500
## iron 3.2643950 2.1029997
## multi 1.2199418 0.7859151
## magnesium 20.4223051 13.1565271
## zinc 0.1964943 0.1265862
## No.Supplement 19.0979358 12.3033374

res1 <- cor.mtest(contrib, conf.level = 0.95)

col2 <- colorRampPalette(c("#67001F", "#B2182B", "#D6604D", "#F4A582",
 "#FDDBC7", "#FFFFFF", "#D1E5F0", "#92C5DE",
 "#4393C3", "#2166AC", "#053061"))


pearson<-chisq$residuals


corrplot(contrib, p.mat = res1$p, insig = "blank",pch.col=1,is.cor=F,tl.col=1, cl.pos="b", number.digits=2,number.cex=0.5,cl.cex=1,cl.length=3, cl.lim=c(0,50), col=rev(col2(200)))


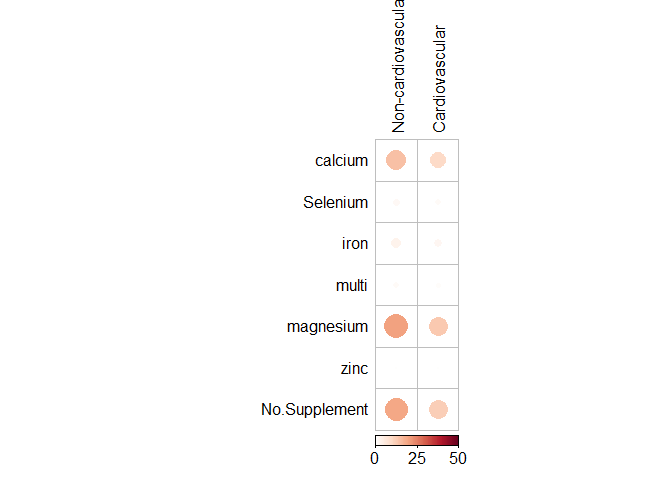


This figure demonstrates which supplement user groups have the largest impact on the significance differences in cardiovascular disease prevalence.

corrplot(pearson, p.mat = res1$p, insig = "blank",pch.col=1,is.cor=F,tl.col=1, cl.pos="b", number.digits=2,number.cex=0.5,cl.cex=1,cl.length=3, cl.lim=c(-4,4),col=rev(col2(200)))


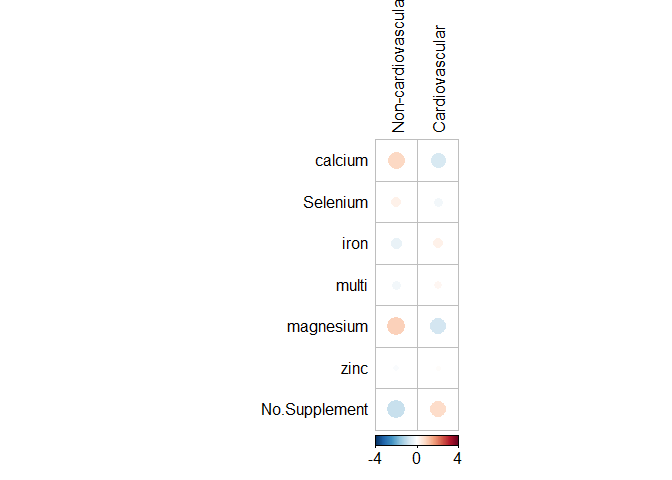


This code demonstrates the directional association between supplement user groups and cardiovascular disease prevalence. No supplement use was associated with the highest cardiovascular disease prevalence while calcium and magnesium use was associated with lower cardiovascular disease prevalence.

#### Diabetes

his code investigates the association between supplement use and diabetes prevalance.

calcium<-table(initialdata$diab,by=initialdata$calcium)[,2]
Selenium<-table(initialdata$diab,by=initialdata$Selenium)[,2]
iron<-table(initialdata$diab,by=initialdata$iron)[,2]
multi<-table(initialdata$diab,by=initialdata$multi)[,2]
magnesium<-table(initialdata$diab,by=initialdata$magnesium)[,2]
zinc<-table(initialdata$diab,by=initialdata$zinc)[,2]
No.Supplement<-table(initialdata$headache,by=initialdata$No.Supplement)[,2]


Diabetes.Pathology.Table<-data.frame(t(cbind(calcium,Selenium,iron,multi,magnesium,zinc,No.Supplement)))
names(Diabetes.Pathology.Table)<-c("Non-Diabetes","Diabetes")
Diabetes.Pathology.Table$percent<-Diabetes.Pathology.Table[,2]/(Cardiovascular.Pathology.Table[,2]+Diabetes.Pathology.Table[,1])*100
Diabetes.Pathology.Table

## Non-Diabetes Diabetes percent
## calcium 552 37 4.120267
## Selenium 41 2 3.030303
## iron 97 14 8.383234
## multi 477 47 5.882353
## magnesium 153 9 3.688525
## zinc 58 7 7.142857
## No.Supplement 613 42 4.093567

chisq.test(Diabetes.Pathology.Table[,1:2])

## Warning in chisq.test(Diabetes.Pathology.Table[, 1:2]): Chi-squared
## approximation may be incorrect

##
## Pearson's Chi-squared test
##
## data: Diabetes.Pathology.Table[, 1:2]
## X-squared = 10.704, df = 6, p-value = 0.09797

## MMSE

Mini-mental state examination (MMSE) is a cognitive assessment with a focus on memory that is often used as the primary measure of Alzheimer’s disease progression in clinical trials. It is a score out of 30 with higher scores corresponding to better cognitive performance.

### Dependent variable check

Those with no MMSE score were removed and variables were checked for correct categorization.

data<-read.csv("C:/Users/mqbssjrn/Dropbox/Science/Projects/Epidemiology ADNI/Zinc/CleanedFinalDataZinc2.csv", header=T)
MMSEdata<-data[!is.na(data$MMSE),]
MMSEdata$ID<-as.factor(MMSEdata$ID)
MMSEdata$APOE4<-as.factor(MMSEdata$APOE4)

### Generating model matrix for each supplement MMSE

#### Building the models for supplement use

This code constructs negative binomial generalized linear mixed models (GLMM) with all significant nuisance variables and each supplement user group independently and then compares them to a baseline model (with no supplement variables) using log-likelihood chi-squared comparison methods.

magnesium<-glmmadmb(neg.b.MMSE~M+AGE+ APOE4+Gender+ edu.cat + diagn+ edu.cat*M+diagn*M+APOE4*M+Gender*M+magnesium*M+ (1|ID), family="nbinom1", data=MMSEdata)

calcium<-glmmadmb(neg.b.MMSE~M+AGE+ APOE4+Gender+ edu.cat + diagn+ edu.cat*M+diagn*M+APOE4*M+Gender*M+calcium*M+ (1|ID), family="nbinom1", data=MMSEdata)

zinc<-glmmadmb(neg.b.MMSE~M+AGE+ APOE4+Gender+ edu.cat + diagn+ edu.cat*M+diagn*M+APOE4*M+Gender*M+zinc*M+ (1|ID), family="nbinom1", data=MMSEdata)

Selenium<-glmmadmb(neg.b.MMSE~M+AGE+ APOE4+Gender+ edu.cat + diagn+ edu.cat*M+diagn*M+APOE4*M+Gender*M+Selenium*M+ (1|ID), family="nbinom1", data=MMSEdata)

multi<-glmmadmb(neg.b.MMSE~M+AGE+ APOE4+Gender+ edu.cat + diagn+ edu.cat*M+diagn*M+APOE4*M+Gender*M+multi*M+ (1|ID), family="nbinom1", data=MMSEdata)

iron<-glmmadmb(neg.b.MMSE~M+AGE+ APOE4+Gender+ edu.cat + diagn+ edu.cat*M+diagn*M+APOE4*M+Gender*M+iron*M+ (1|ID), family="nbinom1", data=MMSEdata)

Supplement<-glmmadmb(neg.b.MMSE~M+AGE+ APOE4+Gender+ edu.cat + diagn+ edu.cat*M+diagn*M+APOE4*M+Gender*M+Supplement*M+ (1|ID), family="nbinom1", data=MMSEdata)

Base<-glmmadmb(neg.b.MMSE~M+AGE+ APOE4+Gender+ edu.cat + diagn+ edu.cat*M+diagn*M+APOE4*M+Gender*M+ (1|ID), family="nbinom1", data=MMSEdata)

anova(magnesium,Base)

## Warning in anova.glmmadmb(magnesium, Base): rearranging models in order of
## increasing complexity

## Analysis of Deviance Table
##
## Model 1: neg.b.MMSE ~ M + AGE + APOE4 + Gender + edu.cat + diagn + edu.cat * M + diagn * M + APOE4 * M + Gender * M
## Model 2: neg.b.MMSE ~ M + AGE + APOE4 + Gender + edu.cat + diagn + edu.cat * M + diagn * M + APOE4 * M + Gender * M + magnesium * M
## NoPar LogLik Df Deviance Pr(>Chi)
## 1 23 -16950
## 2 25 -16948 2 4.8 0.09072 .
## ---
## Signif. codes: 0 '***' 0.001 '**' 0.01 '*' 0.05 '.' 0.1 ' ' 1

anova(calcium,Base)

## Warning in anova.glmmadmb(calcium, Base): rearranging models in order of
## increasing complexity

## Analysis of Deviance Table
##
## Model 1: neg.b.MMSE ~ M + AGE + APOE4 + Gender + edu.cat + diagn + edu.cat * M + diagn * M + APOE4 * M + Gender * M
## Model 2: neg.b.MMSE ~ M + AGE + APOE4 + Gender + edu.cat + diagn + edu.cat * M + diagn * M + APOE4 * M + Gender * M + calcium * M
## NoPar LogLik Df Deviance Pr(>Chi)
## 1 23 -16950
## 2 25 -16947 2 6.8 0.03337 *
## ---
## Signif. codes: 0 '***' 0.001 '**' 0.01 '*' 0.05 '.' 0.1 ' ' 1

anova(zinc,Base)

## Warning in anova.glmmadmb(zinc, Base): rearranging models in order of
## increasing complexity

## Analysis of Deviance Table
##
## Model 1: neg.b.MMSE ~ M + AGE + APOE4 + Gender + edu.cat + diagn + edu.cat * M + diagn * M + APOE4 * M + Gender * M
## Model 2: neg.b.MMSE ~ M + AGE + APOE4 + Gender + edu.cat + diagn + edu.cat * M + diagn * M + APOE4 * M + Gender * M + zinc * M
## NoPar LogLik Df Deviance Pr(>Chi)
## 1 23 -16950
## 2 25 -16947 2 12.82 0.005081 *
## ---
## Signif. codes: 0 '***' 0.001 '**' 0.01 '*' 0.05 '.' 0.1 ' ' 1

anova(Selenium,Base)

## Warning in anova.glmmadmb(Selenium, Base): rearranging models in order of
## increasing complexity

## Analysis of Deviance Table
##
## Model 1: neg.b.MMSE ~ M + AGE + APOE4 + Gender + edu.cat + diagn + edu.cat * M + diagn * M + APOE4 * M + Gender * M
## Model 2: neg.b.MMSE ~ M + AGE + APOE4 + Gender + edu.cat + diagn + edu.cat * M + diagn * M + APOE4 * M + Gender * M + Selenium * M
## NoPar LogLik Df Deviance Pr(>Chi)
## 1 23 -16950
## 2 25 -16948 2 4.8 0.09072 .
## ---
## Signif. codes: 0 '***' 0.001 '**' 0.01 '*' 0.05 '.' 0.1 ' ' 1

anova(multi,Base)

## Warning in anova.glmmadmb(multi, Base): rearranging models in order of
## increasing complexity

## Analysis of Deviance Table
##
## Model 1: neg.b.MMSE ~ M + AGE + APOE4 + Gender + edu.cat + diagn + edu.cat * M + diagn * M + APOE4 * M + Gender * M
## Model 2: neg.b.MMSE ~ M + AGE + APOE4 + Gender + edu.cat + diagn + edu.cat * M + diagn * M + APOE4 * M + Gender * M + multi * M
## NoPar LogLik Df Deviance Pr(>Chi)
## 1 23 -16950
## 2 25 -16950 2 0.2 0.9048

anova(iron,Base)

## Warning in anova.glmmadmb(iron, Base): rearranging models in order of
## increasing complexity

## Analysis of Deviance Table
##
## Model 1: neg.b.MMSE ~ M + AGE + APOE4 + Gender + edu.cat + diagn + edu.cat * M + diagn * M + APOE4 * M + Gender * M
## Model 2: neg.b.MMSE ~ M + AGE + APOE4 + Gender + edu.cat + diagn + edu.cat * M + diagn * M + APOE4 * M + Gender * M + iron * M
## NoPar LogLik Df Deviance Pr(>Chi)
## 1 23 -16950
## 2 25 -16947 2 6.2 0.04505 *
## ---
## Signif. codes: 0 '***' 0.001 '**' 0.01 '*' 0.05 '.' 0.1 ' ' 1

anova(Supplement,Base)

## Warning in anova.glmmadmb(Supplement, Base): rearranging models in order of
## increasing complexity

## Analysis of Deviance Table
##
## Model 1: neg.b.MMSE ~ M + AGE + APOE4 + Gender + edu.cat + diagn + edu.cat * M + diagn * M + APOE4 * M + Gender * M
## Model 2: neg.b.MMSE ~ M + AGE + APOE4 + Gender + edu.cat + diagn + edu.cat * M + diagn * M + APOE4 * M + Gender * M + Supplement * M
## NoPar LogLik Df Deviance Pr(>Chi)
## 1 23 -16950
## 2 25 -16949 2 1.4 0.4966

#### Summary of models

This code produces summaries of each supplement GLMM.

summary(magnesium)

##
## Call:
## glmmadmb(formula = neg.b.MMSE ~ M + AGE + APOE4 + Gender + edu.cat +
## diagn + edu.cat * M + diagn * M + APOE4 * M + Gender * M +
## magnesium * M + (1 | ID), data = MMSEdata, family = "nbinom1")
##
## AIC: 33945
##
## Coefficients:
## Estimate Std. Error z value Pr(>|z|)
## (Intercept) -0.638218 0.059600 -10.71 < 2e-16 ***
## M 0.010692 0.000834 12.82 < 2e-16 ***
## AGE 0.017533 0.002599 6.75 1.5e-11 ***
## APOE41 0.125857 0.042525 2.96 0.00308 **
## APOE42 0.229623 0.064917 3.54 0.00040 ***
## GenderMale 0.088890 0.040260 2.21 0.02725 *
## edu.cat2tertiary 0.192205 0.049594 3.88 0.00011 ***
## edu.cat3mid 0.353488 0.055672 6.35 2.2e-10 ***
## edu.cat4early 0.357966 0.057544 6.22 4.9e-10 ***
## diagn2EMCI 0.707251 0.065789 10.75 < 2e-16 ***
## diagn3LMCI 1.242780 0.055540 22.38 < 2e-16 ***
## diagn4AD 2.115763 0.061946 34.15 < 2e-16 ***
## magnesium -0.112330 0.066663 -1.69 0.09198 .
## M:edu.cat2tertiary -0.003525 0.000740 -4.76 1.9e-06 ***
## M:edu.cat3mid -0.002070 0.000810 -2.56 0.01059 *
## M:edu.cat4early -0.002622 0.000817 -3.21 0.00133 **
## M:diagn2EMCI -0.003302 0.001040 -3.18 0.00150 **
## M:diagn3LMCI 0.002963 0.000724 4.09 4.2e-05 ***
## M:diagn4AD 0.008693 0.001434 6.06 1.3e-09 ***
## M:APOE41 0.005990 0.000628 9.53 < 2e-16 ***
## M:APOE42 0.007648 0.000920 8.31 < 2e-16 ***
## M:GenderMale -0.001940 0.000596 -3.26 0.00113 **
## M:magnesium -0.000590 0.000859 -0.69 0.49260
## ---
## Signif. codes: 0 '***' 0.001 '**' 0.01 '*' 0.05 '.' 0.1 ' ' 1
##
## Number of observations: total=8878, ID=1619
## Random effect variance(s):

## Warning in .local(x, sigma, ...): 'sigma' and 'rdig' arguments are present
## for compatibility only: ignored

## Group=ID
## Variance StdDev
## (Intercept) 0.415 0.6442
##
## Negative binomial dispersion parameter: 1.0597 (std. err.: 0.018237)
##
## Log-likelihood: -16947.5

summary(calcium)

##
## Call:
## glmmadmb(formula = neg.b.MMSE ~ M + AGE + APOE4 + Gender + edu.cat +
## diagn + edu.cat * M + diagn * M + APOE4 * M + Gender * M +
## calcium * M + (1 | ID), data = MMSEdata, family = "nbinom1")
##
## AIC: 33943
##
## Coefficients:
## Estimate Std. Error z value Pr(>|z|)
## (Intercept) -0.596870 0.064256 -9.29 < 2e-16 ***
## M 0.009872 0.000908 10.87 < 2e-16 ***
## AGE 0.017914 0.002599 6.89 5.4e-12 ***
## APOE41 0.125674 0.042439 2.96 0.00306 **
## APOE42 0.233798 0.064884 3.60 0.00031 ***
## GenderMale 0.060902 0.042528 1.43 0.15213
## edu.cat2tertiary 0.193039 0.049595 3.89 9.9e-05 ***
## edu.cat3mid 0.357290 0.055644 6.42 1.4e-10 ***
## edu.cat4early 0.356382 0.057551 6.19 5.9e-10 ***
## diagn2EMCI 0.706762 0.065756 10.75 < 2e-16 ***
## diagn3LMCI 1.243677 0.055466 22.42 < 2e-16 ***
## diagn4AD 2.109152 0.061991 34.02 < 2e-16 ***
## calcium -0.098797 0.043046 -2.30 0.02172 *
## M:edu.cat2tertiary -0.003654 0.000742 -4.92 8.5e-07 ***
## M:edu.cat3mid -0.002115 0.000810 -2.61 0.00899 **
## M:edu.cat4early -0.002714 0.000818 -3.32 0.00091 ***
## M:diagn2EMCI -0.003167 0.001041 -3.04 0.00236 **
## M:diagn3LMCI 0.003034 0.000723 4.19 2.7e-05 ***
## M:diagn4AD 0.009067 0.001436 6.31 2.7e-10 ***
## M:APOE41 0.005836 0.000618 9.44 < 2e-16 ***
## M:APOE42 0.007470 0.000922 8.10 5.5e-16 ***
## M:GenderMale -0.001458 0.000628 -2.32 0.02029 *
## M:calcium 0.001172 0.000607 1.93 0.05361 .
## ---
## Signif. codes: 0 '***' 0.001 '**' 0.01 '*' 0.05 '.' 0.1 ' ' 1
##
## Number of observations: total=8878, ID=1619
## Random effect variance(s):

## Warning in .local(x, sigma, ...): 'sigma' and 'rdig' arguments are present
## for compatibility only: ignored

## Group=ID
## Variance StdDev
## (Intercept) 0.4145 0.6438
##
## Negative binomial dispersion parameter: 1.0591 (std. err.: 0.018237)
##
## Log-likelihood: -16946.5

summary(zinc)

##
## Call:
## glmmadmb(formula = neg.b.MMSE ~ M + AGE + APOE4 + Gender + edu.cat +
## diagn + edu.cat * M + diagn * M + APOE4 * M + Gender * M +
## zinc * M + (1 | ID), data = MMSEdata, family = "nbinom1")
##
## AIC: 33944.2
##
## Coefficients:
## Estimate Std. Error z value Pr(>|z|)
## (Intercept) -0.652901 0.058791 -11.11 < 2e-16 ***
## M 0.010598 0.000823 12.87 < 2e-16 ***
## AGE 0.017688 0.002600 6.80 1.0e-11 ***
## APOE41 0.125451 0.042497 2.95 0.00316 **
## APOE42 0.229463 0.064976 3.53 0.00041 ***
## GenderMale 0.094476 0.040176 2.35 0.01870 *
## edu.cat2tertiary 0.191208 0.049710 3.85 0.00012 ***
## edu.cat3mid 0.353553 0.055711 6.35 2.2e-10 ***
## edu.cat4early 0.358668 0.057586 6.23 4.7e-10 ***
## diagn2EMCI 0.710291 0.065807 10.79 < 2e-16 ***
## diagn3LMCI 1.245268 0.055508 22.43 < 2e-16 ***
## diagn4AD 2.122853 0.061754 34.38 < 2e-16 ***
## zinc -0.071166 0.099721 -0.71 0.47545
## M:edu.cat2tertiary -0.003384 0.000745 -4.54 5.5e-06 ***
## M:edu.cat3mid -0.001962 0.000810 -2.42 0.01547 *
## M:edu.cat4early -0.002696 0.000818 -3.30 0.00098 ***
## M:diagn2EMCI -0.003217 0.001040 -3.09 0.00198 **
## M:diagn3LMCI 0.003138 0.000726 4.32 1.6e-05 ***
## M:diagn4AD 0.008776 0.001429 6.14 8.1e-10 ***
## M:APOE41 0.005915 0.000617 9.58 < 2e-16 ***
## M:APOE42 0.007510 0.000920 8.17 3.1e-16 ***
## M:GenderMale -0.001867 0.000590 -3.16 0.00156 **
## M:zinc -0.002074 0.000740 -2.80 0.00511 **
## ---
## Signif. codes: 0 '***' 0.001 '**' 0.01 '*' 0.05 '.' 0.1 ' ' 1
##
## Number of observations: total=8878, ID=1619
## Random effect variance(s):

## Warning in .local(x, sigma, ...): 'sigma' and 'rdig' arguments are present
## for compatibility only: ignored

## Group=ID
## Variance StdDev
## (Intercept) 0.4159 0.6449
##
## Negative binomial dispersion parameter: 1.0594 (std. err.: 0.018222)
##
## Log-likelihood: -16947.1

summary(Selenium)

##
## Call:
## glmmadmb(formula = neg.b.MMSE ~ M + AGE + APOE4 + Gender + edu.cat +
## diagn + edu.cat * M + diagn * M + APOE4 * M + Gender * M +
## Selenium * M + (1 | ID), data = MMSEdata, family = "nbinom1")
##
## AIC: 33945
##
## Coefficients:
## Estimate Std. Error z value Pr(>|z|)
## (Intercept) -0.652553 0.058992 -11.06 < 2e-16 ***
## M 0.010777 0.000831 12.98 < 2e-16 ***
## AGE 0.017721 0.002599 6.82 9.3e-12 ***
## APOE41 0.125002 0.042497 2.94 0.00327 **
## APOE42 0.228198 0.064961 3.51 0.00044 ***
## GenderMale 0.094681 0.040168 2.36 0.01842 *
## edu.cat2tertiary 0.192349 0.049698 3.87 0.00011 ***
## edu.cat3mid 0.352283 0.055785 6.32 2.7e-10 ***
## edu.cat4early 0.356853 0.057728 6.18 6.3e-10 ***
## diagn2EMCI 0.708626 0.065875 10.76 < 2e-16 ***
## diagn3LMCI 1.245981 0.055493 22.45 < 2e-16 ***
## diagn4AD 2.124003 0.061726 34.41 < 2e-16 ***
## Selenium -0.102694 0.122390 -0.84 0.40143
## M:edu.cat2tertiary -0.003755 0.000749 -5.02 5.3e-07 ***
## M:edu.cat3mid -0.002140 0.000811 -2.64 0.00828 **
## M:edu.cat4early -0.002830 0.000826 -3.43 0.00061 ***
## M:diagn2EMCI -0.003400 0.001042 -3.26 0.00110 **
## M:diagn3LMCI 0.003001 0.000723 4.15 3.3e-05 ***
## M:diagn4AD 0.008685 0.001430 6.07 1.3e-09 ***
## M:APOE41 0.005940 0.000618 9.61 < 2e-16 ***
## M:APOE42 0.007737 0.000922 8.39 < 2e-16 ***
## M:GenderMale -0.001855 0.000590 -3.14 0.00168 **
## M:Selenium -0.002183 0.001370 -1.59 0.11098
## ---
## Signif. codes: 0 '***' 0.001 '**' 0.01 '*' 0.05 '.' 0.1 ' ' 1
##
## Number of observations: total=8878, ID=1619
## Random effect variance(s):

## Warning in .local(x, sigma, ...): 'sigma' and 'rdig' arguments are present
## for compatibility only: ignored

## Group=ID
## Variance StdDev
## (Intercept) 0.4155 0.6446
##
## Negative binomial dispersion parameter: 1.06 (std. err.: 0.018238)
##
## Log-likelihood: -16947.5

summary(multi)

##
## Call:
## glmmadmb(formula = neg.b.MMSE ~ M + AGE + APOE4 + Gender + edu.cat +
## diagn + edu.cat * M + diagn * M + APOE4 * M + Gender * M +
## multi * M + (1 | ID), data = MMSEdata, family = "nbinom1")
##
## AIC: 33949.6
##
## Coefficients:
## Estimate Std. Error z value Pr(>|z|)
## (Intercept) -0.655284 0.060855 -10.77 < 2e-16 ***
## M 0.010449 0.000887 11.78 < 2e-16 ***
## AGE 0.017733 0.002602 6.82 9.4e-12 ***
## APOE41 0.125337 0.042525 2.95 0.0032 **
## APOE42 0.230228 0.064998 3.54 0.0004 ***
## GenderMale 0.093904 0.040264 2.33 0.0197 *
## edu.cat2tertiary 0.192811 0.049652 3.88 0.0001 ***
## edu.cat3mid 0.355277 0.055721 6.38 1.8e-10 ***
## edu.cat4early 0.357916 0.057620 6.21 5.2e-10 ***
## diagn2EMCI 0.710312 0.066155 10.74 < 2e-16 ***
## diagn3LMCI 1.247559 0.055542 22.46 < 2e-16 ***
## diagn4AD 2.125022 0.061881 34.34 < 2e-16 ***
## multi -0.003514 0.041497 -0.08 0.9325
## M:edu.cat2tertiary -0.003562 0.000741 -4.81 1.5e-06 ***
## M:edu.cat3mid -0.002054 0.000809 -2.54 0.0112 *
## M:edu.cat4early -0.002616 0.000817 -3.20 0.0014 **
## M:diagn2EMCI -0.003195 0.001058 -3.02 0.0025 **
## M:diagn3LMCI 0.003040 0.000731 4.16 3.2e-05 ***
## M:diagn4AD 0.008861 0.001438 6.16 7.2e-10 ***
## M:APOE41 0.005898 0.000618 9.54 < 2e-16 ***
## M:APOE42 0.007583 0.000920 8.25 < 2e-16 ***
## M:GenderMale -0.001894 0.000594 -3.19 0.0014 **
## M:multi 0.000236 0.000582 0.41 0.6848
## ---
## Signif. codes: 0 '***' 0.001 '**' 0.01 '*' 0.05 '.' 0.1 ' ' 1
##
## Number of observations: total=8878, ID=1619
## Random effect variance(s):

## Warning in .local(x, sigma, ...): 'sigma' and 'rdig' arguments are present
## for compatibility only: ignored

## Group=ID
## Variance StdDev
## (Intercept) 0.4162 0.6451
##
## Negative binomial dispersion parameter: 1.0595 (std. err.: 0.018241)
##
## Log-likelihood: -16949.8

summary(iron)

##
## Call:
## glmmadmb(formula = neg.b.MMSE ~ M + AGE + APOE4 + Gender + edu.cat +
## diagn + edu.cat * M + diagn * M + APOE4 * M + Gender * M +
## iron * M + (1 | ID), data = MMSEdata, family = "nbinom1")
##
## AIC: 33943.6
##
## Coefficients:
## Estimate Std. Error z value Pr(>|z|)
## (Intercept) -0.668083 0.059212 -11.28 < 2e-16 ***
## M 0.010745 0.000826 13.00 < 2e-16 ***
## AGE 0.017734 0.002607 6.80 1.0e-11 ***
## APOE41 0.131173 0.042624 3.08 0.00209 **
## APOE42 0.232867 0.065087 3.58 0.00035 ***
## GenderMale 0.094782 0.040257 2.35 0.01855 *
## edu.cat2tertiary 0.191256 0.049715 3.85 0.00012 ***
## edu.cat3mid 0.349116 0.055852 6.25 4.1e-10 ***
## edu.cat4early 0.353085 0.057720 6.12 9.5e-10 ***
## diagn2EMCI 0.709469 0.065883 10.77 < 2e-16 ***
## diagn3LMCI 1.245153 0.055609 22.39 < 2e-16 ***
## diagn4AD 2.132314 0.061908 34.44 < 2e-16 ***
## iron 0.121259 0.075887 1.60 0.11007
## M:edu.cat2tertiary -0.003464 0.000740 -4.68 2.9e-06 ***
## M:edu.cat3mid -0.001866 0.000812 -2.30 0.02161 *
## M:edu.cat4early -0.002427 0.000821 -2.96 0.00310 **
## M:diagn2EMCI -0.003220 0.001039 -3.10 0.00194 **
## M:diagn3LMCI 0.003173 0.000726 4.37 1.2e-05 ***
## M:diagn4AD 0.008702 0.001428 6.09 1.1e-09 ***
## M:APOE41 0.005679 0.000623 9.11 < 2e-16 ***
## M:APOE42 0.007450 0.000920 8.10 5.6e-16 ***
## M:GenderMale -0.001869 0.000590 -3.17 0.00153 **
## M:iron -0.001918 0.000826 -2.32 0.02023 *
## ---
## Signif. codes: 0 '***' 0.001 '**' 0.01 '*' 0.05 '.' 0.1 ' ' 1
##
## Number of observations: total=8878, ID=1619
## Random effect variance(s):

## Warning in .local(x, sigma, ...): 'sigma' and 'rdig' arguments are present
## for compatibility only: ignored

## Group=ID
## Variance StdDev
## (Intercept) 0.4178 0.6464
##
## Negative binomial dispersion parameter: 1.0577 (std. err.: 0.018219)
##
## Log-likelihood: -16946.8

summary(Supplement)

##
## Call:
## glmmadmb(formula = neg.b.MMSE ~ M + AGE + APOE4 + Gender + edu.cat +
## diagn + edu.cat * M + diagn * M + APOE4 * M + Gender * M +
## Supplement * M + (1 | ID), data = MMSEdata, family = "nbinom1")
##
## AIC: 33948.4
##
## Coefficients:
## Estimate Std. Error z value Pr(>|z|)
## (Intercept) -0.628333 0.066606 -9.43 < 2e-16 ***
## M 0.010797 0.000987 10.94 < 2e-16 ***
## AGE 0.017849 0.002603 6.86 7.0e-12 ***
## APOE41 0.123918 0.042523 2.91 0.00357 **
## APOE42 0.228148 0.064983 3.51 0.00045 ***
## GenderMale 0.087720 0.040632 2.16 0.03086 *
## edu.cat2tertiary 0.190767 0.049659 3.84 0.00012 ***
## edu.cat3mid 0.355656 0.055717 6.38 1.7e-10 ***
## edu.cat4early 0.355393 0.057640 6.17 7.0e-10 ***
## diagn2EMCI 0.708793 0.065908 10.75 < 2e-16 ***
## diagn3LMCI 1.247208 0.055530 22.46 < 2e-16 ***
## diagn4AD 2.120304 0.062191 34.09 < 2e-16 ***
## Supplement -0.037599 0.040245 -0.93 0.35018
## M:edu.cat2tertiary -0.003519 0.000741 -4.75 2.1e-06 ***
## M:edu.cat3mid -0.002044 0.000810 -2.52 0.01160 *
## M:edu.cat4early -0.002619 0.000817 -3.21 0.00135 **
## M:diagn2EMCI -0.003320 0.001044 -3.18 0.00148 **
## M:diagn3LMCI 0.002957 0.000725 4.08 4.5e-05 ***
## M:diagn4AD 0.008713 0.001443 6.04 1.6e-09 ***
## M:APOE41 0.005893 0.000617 9.55 < 2e-16 ***
## M:APOE42 0.007635 0.000921 8.29 < 2e-16 ***
## M:GenderMale -0.001884 0.000594 -3.17 0.00152 **
## M:Supplement -0.000234 0.000651 -0.36 0.71872
## ---
## Signif. codes: 0 '***' 0.001 '**' 0.01 '*' 0.05 '.' 0.1 ' ' 1
##
## Number of observations: total=8878, ID=1619
## Random effect variance(s):

## Warning in .local(x, sigma, ...): 'sigma' and 'rdig' arguments are present
## for compatibility only: ignored

## Group=ID
## Variance StdDev
## (Intercept) 0.4161 0.645
##
## Negative binomial dispersion parameter: 1.0594 (std. err.: 0.018229)
##
## Log-likelihood: -16949.2

#### Evaluating the significance of the main effect and interaction effect of supplement use.

This code evaluates the interaction effect of supplement with time and the main effect of supplement independently using the methods described above.

magnesium.main<-glmmadmb(neg.b.MMSE~M+AGE+ APOE4+Gender+ edu.cat + diagn+ edu.cat*M+diagn*M+APOE4*M+Gender*M+magnesium+ (1|ID), family="nbinom1", data=MMSEdata)

magnesium.prog<-glmmadmb(neg.b.MMSE~M+AGE+ APOE4+Gender+ edu.cat + diagn+ edu.cat*M+diagn*M+APOE4*M+Gender*M+magnesium*M+ (1|ID), family="nbinom1", data=MMSEdata)

calcium.main<-glmmadmb(neg.b.MMSE~M+AGE+ APOE4+Gender+ edu.cat + diagn+ edu.cat*M+diagn*M+APOE4*M+Gender*M+calcium+ (1|ID), family="nbinom1", data=MMSEdata)

calcium.prog<-glmmadmb(neg.b.MMSE~M+AGE+ APOE4+Gender+ edu.cat + diagn+ edu.cat*M+diagn*M+APOE4*M+Gender*M+calcium*M+ (1|ID), family="nbinom1", data=MMSEdata)

zinc.main<-glmmadmb(neg.b.MMSE~M+AGE+ APOE4+Gender+ edu.cat + diagn+ edu.cat*M+diagn*M+APOE4*M+Gender*M+zinc+ (1|ID), family="nbinom1", data=MMSEdata)

zinc.prog<-glmmadmb(neg.b.MMSE~M+AGE+ APOE4+Gender+ edu.cat + diagn+ edu.cat*M+diagn*M+APOE4*M+Gender*M+zinc*M+ (1|ID), family="nbinom1", data=MMSEdata)

Selenium.main<-glmmadmb(neg.b.MMSE~M+AGE+ APOE4+Gender+ edu.cat + diagn+ edu.cat*M+diagn*M+APOE4*M+Gender*M+Selenium + (1|ID), family="nbinom1", data=MMSEdata)

Selenium.prog<-glmmadmb(neg.b.MMSE~M+AGE+ APOE4+Gender+ edu.cat + diagn+ edu.cat*M+diagn*M+APOE4*M+Gender*M+Selenium*M+ (1|ID), family="nbinom1", data=MMSEdata)

multi.main<-glmmadmb(neg.b.MMSE~M+AGE+ APOE4+Gender+ edu.cat + diagn+ edu.cat*M+diagn*M+APOE4*M+Gender*M+ multi+ (1|ID), family="nbinom1", data=MMSEdata)

multi.prog<-glmmadmb(neg.b.MMSE~M+AGE+ APOE4+Gender+ edu.cat + diagn+ edu.cat*M+diagn*M+APOE4*M+Gender*M+ multi*M+ (1|ID), family="nbinom1", data=MMSEdata)

iron.main<-glmmadmb(neg.b.MMSE~M+AGE+ APOE4+Gender+ edu.cat + diagn+ edu.cat*M+diagn*M+APOE4*M+Gender*M+iron + (1|ID), family="nbinom1", data=MMSEdata)

iron.prog<-glmmadmb(neg.b.MMSE~M+AGE+ APOE4+Gender+ edu.cat + diagn+ edu.cat*M+diagn*M+APOE4*M+Gender*M+iron*M+ (1|ID), family="nbinom1", data=MMSEdata)

Supplement.main<-glmmadmb(neg.b.MMSE~M+AGE+ APOE4+Gender+ edu.cat + diagn+ edu.cat*M+diagn*M+APOE4*M+Gender*M+Supplement + (1|ID), family="nbinom1", data=MMSEdata)

Supplement.prog<-glmmadmb(neg.b.MMSE~M+AGE+ APOE4+Gender+ edu.cat + diagn+ edu.cat*M+diagn*M+APOE4*M+Gender*M+Supplement*M+ (1|ID), family="nbinom1", data=MMSEdata)

Base<-glmmadmb(neg.b.MMSE~M+AGE+ APOE4+Gender+ edu.cat + diagn+ edu.cat*M+diagn*M+APOE4*M+Gender*M+ (1|ID), family="nbinom1", data=MMSEdata)

anova(magnesium.main,Base)

## Warning in anova.glmmadmb(magnesium.main, Base): rearranging models in
## order of increasing complexity

## Analysis of Deviance Table
##
## Model 1: neg.b.MMSE ~ M + AGE + APOE4 + Gender + edu.cat + diagn + edu.cat * M + diagn * M + APOE4 * M + Gender * M
## Model 2: neg.b.MMSE ~ M + AGE + APOE4 + Gender + edu.cat + diagn + edu.cat * M + diagn * M + APOE4 * M + Gender * M + magnesium
## NoPar LogLik Df Deviance Pr(>Chi)
## 1 23 -16950
## 2 24 -16948 1 4.4 0.03594 *
## ---
## Signif. codes: 0 '***' 0.001 '**' 0.01 '*' 0.05 '.' 0.1 ' ' 1

anova(calcium.main,Base)

## Warning in anova.glmmadmb(calcium.main, Base): rearranging models in order
## of increasing complexity

## Analysis of Deviance Table
##
## Model 1: neg.b.MMSE ~ M + AGE + APOE4 + Gender + edu.cat + diagn + edu.cat * M + diagn * M + APOE4 * M + Gender * M
## Model 2: neg.b.MMSE ~ M + AGE + APOE4 + Gender + edu.cat + diagn + edu.cat * M + diagn * M + APOE4 * M + Gender * M + calcium
## NoPar LogLik Df Deviance Pr(>Chi)
## 1 23 -16950
## 2 24 -16948 1 3 0.08326 .
## ---
## Signif. codes: 0 '***' 0.001 '**' 0.01 '*' 0.05 '.' 0.1 ' ' 1

anova(zinc.main,Base)

## Warning in anova.glmmadmb(zinc.main, Base): rearranging models in order of
## increasing complexity

## Analysis of Deviance Table
##
## Model 1: neg.b.MMSE ~ M + AGE + APOE4 + Gender + edu.cat + diagn + edu.cat * M + diagn * M + APOE4 * M + Gender * M
## Model 2: neg.b.MMSE ~ M + AGE + APOE4 + Gender + edu.cat + diagn + edu.cat * M + diagn * M + APOE4 * M + Gender * M + zinc
## NoPar LogLik Df Deviance Pr(>Chi)
## 1 23 -16950
## 2 24 -16949 1 2.2 0.138

anova(Selenium.main,Base)

## Warning in anova.glmmadmb(Selenium.main, Base): rearranging models in order
## of increasing complexity

## Analysis of Deviance Table
##
## Model 1: neg.b.MMSE ~ M + AGE + APOE4 + Gender + edu.cat + diagn + edu.cat * M + diagn * M + APOE4 * M + Gender * M
## Model 2: neg.b.MMSE ~ M + AGE + APOE4 + Gender + edu.cat + diagn + edu.cat * M + diagn * M + APOE4 * M + Gender * M + Selenium
## NoPar LogLik Df Deviance Pr(>Chi)
## 1 23 -16950
## 2 24 -16949 1 2.2 0.138

anova(multi.main,Base)

## Warning in anova.glmmadmb(multi.main, Base): rearranging models in order of
## increasing complexity

## Analysis of Deviance Table
##
## Model 1: neg.b.MMSE ~ M + AGE + APOE4 + Gender + edu.cat + diagn + edu.cat * M + diagn * M + APOE4 * M + Gender * M
## Model 2: neg.b.MMSE ~ M + AGE + APOE4 + Gender + edu.cat + diagn + edu.cat * M + diagn * M + APOE4 * M + Gender * M + multi
## NoPar LogLik Df Deviance Pr(>Chi)
## 1 23 -16950
## 2 24 -16950 1 0 1

anova(iron.main,Base)

## Warning in anova.glmmadmb(iron.main, Base): rearranging models in order of
## increasing complexity

## Analysis of Deviance Table
##
## Model 1: neg.b.MMSE ~ M + AGE + APOE4 + Gender + edu.cat + diagn + edu.cat * M + diagn * M + APOE4 * M + Gender * M
## Model 2: neg.b.MMSE ~ M + AGE + APOE4 + Gender + edu.cat + diagn + edu.cat * M + diagn * M + APOE4 * M + Gender * M + iron
## NoPar LogLik Df Deviance Pr(>Chi)
## 1 23 -16950
## 2 24 -16950 1 0.8 0.3711

anova(Supplement.main,Base)

## Warning in anova.glmmadmb(Supplement.main, Base): rearranging models in
## order of increasing complexity

## Analysis of Deviance Table
##
## Model 1: neg.b.MMSE ~ M + AGE + APOE4 + Gender + edu.cat + diagn + edu.cat * M + diagn * M + APOE4 * M + Gender * M
## Model 2: neg.b.MMSE ~ M + AGE + APOE4 + Gender + edu.cat + diagn + edu.cat * M + diagn * M + APOE4 * M + Gender * M + Supplement
## NoPar LogLik Df Deviance Pr(>Chi)
## 1 23 -16950
## 2 24 -16949 1 1.2 0.2733

anova(magnesium.prog,magnesium.main)

## Warning in anova.glmmadmb(magnesium.prog, magnesium.main): rearranging
## models in order of increasing complexity

## Analysis of Deviance Table
##
## Model 1: neg.b.MMSE ~ M + AGE + APOE4 + Gender + edu.cat + diagn + edu.cat * M + diagn * M + APOE4 * M + Gender * M + magnesium
## Model 2: neg.b.MMSE ~ M + AGE + APOE4 + Gender + edu.cat + diagn + edu.cat * M + diagn * M + APOE4 * M + Gender * M + magnesium * M
## NoPar LogLik Df Deviance Pr(>Chi)
## 1 24 -16948
## 2 25 -16948 1 0.4 0.5271

anova(calcium.prog,calcium.main)

## Warning in anova.glmmadmb(calcium.prog, calcium.main): rearranging models
## in order of increasing complexity

## Analysis of Deviance Table
##
## Model 1: neg.b.MMSE ~ M + AGE + APOE4 + Gender + edu.cat + diagn + edu.cat * M + diagn * M + APOE4 * M + Gender * M + calcium
## Model 2: neg.b.MMSE ~ M + AGE + APOE4 + Gender + edu.cat + diagn + edu.cat * M + diagn * M + APOE4 * M + Gender * M + calcium * M
## NoPar LogLik Df Deviance Pr(>Chi)
## 1 24 -16948
## 2 25 -16947 1 3.8 0.05125 .
## ---
## Signif. codes: 0 '***' 0.001 '**' 0.01 '*' 0.05 '.' 0.1 ' ' 1

anova(zinc.prog,zinc.main)

## Warning in anova.glmmadmb(zinc.prog, zinc.main): rearranging models in
## order of increasing complexity

## Analysis of Deviance Table
##
## Model 1: neg.b.MMSE ~ M + AGE + APOE4 + Gender + edu.cat + diagn + edu.cat * M + diagn * M + APOE4 * M + Gender * M + zinc
## Model 2: neg.b.MMSE ~ M + AGE + APOE4 + Gender + edu.cat + diagn + edu.cat * M + diagn * M + APOE4 * M + Gender * M + zinc * M
## NoPar LogLik Df Deviance Pr(>Chi)
## 1 24 -16949
## 2 25 -16943 1 7.4 0.00540 .
## ---
## Signif. codes: 0 '***' 0.001 '**' 0.01 '*' 0.05 '.' 0.1 ' ' 1

anova(Selenium.prog,Selenium.main)

## Warning in anova.glmmadmb(Selenium.prog, Selenium.main): rearranging models
## in order of increasing complexity

## Analysis of Deviance Table
##
## Model 1: neg.b.MMSE ~ M + AGE + APOE4 + Gender + edu.cat + diagn + edu.cat * M + diagn * M + APOE4 * M + Gender * M + Selenium
## Model 2: neg.b.MMSE ~ M + AGE + APOE4 + Gender + edu.cat + diagn + edu.cat * M + diagn * M + APOE4 * M + Gender * M + Selenium * M
## NoPar LogLik Df Deviance Pr(>Chi)
## 1 24 -16949
## 2 25 -16948 1 2.6 0.1069

anova(multi.prog,multi.main)

## Warning in anova.glmmadmb(multi.prog, multi.main): rearranging models in
## order of increasing complexity

## Analysis of Deviance Table
##
## Model 1: neg.b.MMSE ~ M + AGE + APOE4 + Gender + edu.cat + diagn + edu.cat * M + diagn * M + APOE4 * M + Gender * M + multi
## Model 2: neg.b.MMSE ~ M + AGE + APOE4 + Gender + edu.cat + diagn + edu.cat * M + diagn * M + APOE4 * M + Gender * M + multi * M
## NoPar LogLik Df Deviance Pr(>Chi)
## 1 24 -16950
## 2 25 -16950 1 0.2 0.6547

anova(iron.prog,iron.main)

## Warning in anova.glmmadmb(iron.prog, iron.main): rearranging models in
## order of increasing complexity

## Analysis of Deviance Table
##
## Model 1: neg.b.MMSE ~ M + AGE + APOE4 + Gender + edu.cat + diagn + edu.cat * M + diagn * M + APOE4 * M + Gender * M + iron
## Model 2: neg.b.MMSE ~ M + AGE + APOE4 + Gender + edu.cat + diagn + edu.cat * M + diagn * M + APOE4 * M + Gender * M + iron * M
## NoPar LogLik Df Deviance Pr(>Chi)
## 1 24 -16950
## 2 25 -16947 1 5.4 0.02014 *
## ---
## Signif. codes: 0 '***' 0.001 '**' 0.01 '*' 0.05 '.' 0.1 ' ' 1

anova(Supplement.prog,Supplement.main)

## Warning in anova.glmmadmb(Supplement.prog, Supplement.main): rearranging
## models in order of increasing complexity

## Analysis of Deviance Table
##
## Model 1: neg.b.MMSE ~ M + AGE + APOE4 + Gender + edu.cat + diagn + edu.cat * M + diagn * M + APOE4 * M + Gender * M + Supplement
## Model 2: neg.b.MMSE ~ M + AGE + APOE4 + Gender + edu.cat + diagn + edu.cat * M + diagn * M + APOE4 * M + Gender * M + Supplement * M
## NoPar LogLik Df Deviance Pr(>Chi)
## 1 24 -16949
## 2 25 -16949 1 0.2 0.6547

#### Generating the model matrices for each Supplement

This code generates model matrices for each supplement which can then be used to build predictive plots of cognitive decline for each supplement model.

##### Magnesium

newdat.magnesium<-expand.grid(ID = 120, M = c(0,6,12,18,24,36,48,60,72,84,96,108,120), AGE = 0,APOE4 = c("0","1","2"), Gender = c("Male","Female"), edu.cat = c(levels(MMSEdata$edu.cat)), diagn=c(levels(MMSEdata$diagn)),magnesium = c(0,1))

mm <- model.matrix(delete.response(terms(magnesium)),newdat.magnesium)


newdat.magnesium$manualEstimate = drop(mm %*% fixef(magnesium))
varMatrix <- mm %*% vcov(magnesium) %*% t(mm)
VarPointEstimates<-diag(varMatrix)

newdat.magnesium$manualSE = sqrt(VarPointEstimates)
newdat.magnesium$predictSE = predict(magnesium, newdata = newdat.magnesium,type = 'link',se.fit = TRUE)$se.fit
newdat.magnesium$MMSEestimate<-30-exp(newdat.magnesium$manualEstimate)
newdat.magnesium$UCI = 30-exp((newdat.magnesium$manualEstimate +1.96*newdat.magnesium$predictSE))
newdat.magnesium$LCI = 30-exp((newdat.magnesium$manualEstimate -1.96*newdat.magnesium$predictSE))

newdat.magnesium.only<-newdat.magnesium[newdat.magnesium$magnesium==1,]
newdat.magnesium.only$Supplement<-rep("magnesium",length(newdat.magnesium.only$magnesium))

newdat.magnesium.only<-subset(newdat.magnesium.only,select=-c(magnesium))

##### Calcium

newdat.calcium<-expand.grid(ID = 120, M = c(0,6,12,18,24,36,48,60,72,84,96,108,120), AGE = 0,APOE4 = c("0","1","2"), Gender = c("Male","Female"), edu.cat = c(levels(MMSEdata$edu.cat)), diagn=c(levels(MMSEdata$diagn)),calcium = c(0,1))

mm <- model.matrix(delete.response(terms(calcium)),newdat.calcium)
newdat.calcium$manualEstimate = drop(mm %*% fixef(calcium))
varMatrix <- mm %*% vcov(calcium) %*% t(mm)
VarPointEstimates<-diag(varMatrix)

newdat.calcium$manualSE = sqrt(VarPointEstimates)
newdat.calcium$predictSE = predict(calcium, newdata = newdat.calcium,type = 'link',se.fit = TRUE)$se.fit
newdat.calcium$MMSEestimate<-30-exp(newdat.calcium$manualEstimate)
newdat.calcium$UCI = 30-exp((newdat.calcium$manualEstimate +1.96*newdat.calcium$predictSE))
newdat.calcium$LCI = 30-exp((newdat.calcium$manualEstimate -1.96*newdat.calcium$predictSE))

newdat.calcium.only<-newdat.calcium[newdat.calcium$calcium==1,]
newdat.calcium.only$Supplement<-rep("calcium",length(newdat.calcium.only$calcium))

newdat.calcium.only<-subset(newdat.calcium.only,select=-c(calcium))

##### Zinc

newdat.zinc<-expand.grid(ID = 120, M = c(0,6,12,18,24,36,48,60,72,84,96,108,120), AGE = 0,APOE4 = c("0","1","2"), Gender = c("Male","Female"), edu.cat = c(levels(MMSEdata$edu.cat)), diagn=c(levels(MMSEdata$diagn)),zinc = c(0,1))

mm <- model.matrix(delete.response(terms(zinc)),newdat.zinc)
newdat.zinc$manualEstimate = drop(mm %*% fixef(zinc))
varMatrix <- mm %*% vcov(zinc) %*% t(mm)
VarPointEstimates<-diag(varMatrix)

newdat.zinc$manualSE = sqrt(VarPointEstimates)
newdat.zinc$predictSE = predict(zinc, newdata = newdat.zinc,type = 'link',se.fit = TRUE)$se.fit
newdat.zinc$MMSEestimate<-30-exp(newdat.zinc$manualEstimate)
newdat.zinc$UCI = 30-exp((newdat.zinc$manualEstimate +1.96*newdat.zinc$predictSE))
newdat.zinc$LCI = 30-exp((newdat.zinc$manualEstimate -1.96*newdat.zinc$predictSE))

newdat.zinc.only<-newdat.zinc[newdat.zinc$zinc==1,]
newdat.zinc.only$Supplement<-rep("zinc",length(newdat.zinc.only$zinc))
newdat.zinc.only<-subset(newdat.zinc.only,select=-c(zinc))

##### Selenium

newdat.Selenium<-expand.grid(ID = 120, M = c(0,6,12,18,24,36,48,60,72,84,96,108,120), AGE = 0,APOE4 = c("0","1","2"), Gender = c("Male","Female"), edu.cat = c(levels(MMSEdata$edu.cat)), diagn=c(levels(MMSEdata$diagn)),Selenium = c(0,1))

mm <- model.matrix(delete.response(terms(Selenium)),newdat.Selenium)
newdat.Selenium$manualEstimate = drop(mm %*% fixef(Selenium))
varMatrix <- mm %*% vcov(Selenium) %*% t(mm)
VarPointEstimates<-diag(varMatrix)

newdat.Selenium$manualSE = sqrt(VarPointEstimates)
newdat.Selenium$predictSE = predict(Selenium, newdata = newdat.Selenium,type = 'link',se.fit = TRUE)$se.fit
newdat.Selenium$MMSEestimate<-30-exp(newdat.Selenium$manualEstimate)
newdat.Selenium$UCI = 30-exp((newdat.Selenium$manualEstimate +1.96*newdat.Selenium$predictSE))
newdat.Selenium$LCI = 30-exp((newdat.Selenium$manualEstimate -1.96*newdat.Selenium$predictSE))

newdat.Selenium.only<-newdat.Selenium[newdat.Selenium$Selenium==1,]
newdat.Selenium.only$Supplement<-rep("Selenium",length(newdat.Selenium.only$Selenium))

newdat.Selenium.only<-subset(newdat.Selenium.only,select=-c(Selenium))

##### Multivitamin

newdat.multi<-expand.grid(ID = 120, M = c(0,6,12,18,24,36,48,60,72,84,96,108,120), AGE = 0,APOE4 = c("0","1","2"), Gender = c("Male","Female"), edu.cat = c(levels(MMSEdata$edu.cat)), diagn=c(levels(MMSEdata$diagn)),multi = c(0,1))

mm <- model.matrix(delete.response(terms(multi)),newdat.multi)
newdat.multi$manualEstimate = drop(mm %*% fixef(multi))
varMatrix <- mm %*% vcov(multi) %*% t(mm)
VarPointEstimates<-diag(varMatrix)

newdat.multi$manualSE = sqrt(VarPointEstimates)
newdat.multi$predictSE = predict(multi, newdata = newdat.multi,type = 'link',se.fit = TRUE)$se.fit
newdat.multi$MMSEestimate<-30-exp(newdat.multi$manualEstimate)
newdat.multi$UCI = 30-exp((newdat.multi$manualEstimate +1.96*newdat.multi$predictSE))
newdat.multi$LCI = 30-exp((newdat.multi$manualEstimate -1.96*newdat.multi$predictSE))

newdat.multi.only<-newdat.multi[newdat.multi$multi==1,]
newdat.multi.only$Supplement<-rep("multi",length(newdat.multi.only$multi))

newdat.multi.only<-subset(newdat.multi.only,select=-c(multi))

##### Iron

newdat.iron<-expand.grid(ID = 120, M = c(0,6,12,18,24,36,48,60,72,84,96,108,120), AGE = 0,APOE4 = c("0","1","2"), Gender = c("Male","Female"), edu.cat = c(levels(MMSEdata$edu.cat)), diagn=c(levels(MMSEdata$diagn)),iron = c(0,1))

mm <- model.matrix(delete.response(terms(iron)),newdat.iron)
newdat.iron$manualEstimate = drop(mm %*% fixef(iron))
varMatrix <- mm %*% vcov(iron) %*% t(mm)
VarPointEstimates<-diag(varMatrix)

newdat.iron$manualSE = sqrt(VarPointEstimates)
newdat.iron$predictSE = predict(iron, newdata = newdat.iron,type = 'link',se.fit = TRUE)$se.fit
newdat.iron$MMSEestimate<-30-exp(newdat.iron$manualEstimate)
newdat.iron$UCI = 30-exp((newdat.iron$manualEstimate +1.96*newdat.iron$predictSE))
newdat.iron$LCI = 30-exp((newdat.iron$manualEstimate -1.96*newdat.iron$predictSE))

newdat.iron.only<-newdat.iron[newdat.iron$iron==1,]
newdat.iron.only$Supplement<-rep("iron",length(newdat.iron.only$iron))

newdat.iron.only<-subset(newdat.iron.only,select=-c(iron))

##### Supplement

newdat.Supplement<-expand.grid(ID = 120, M = c(0,6,12,18,24,36,48,60,72,84,96,108,120), AGE = 0,APOE4 = c("0","1","2"), Gender = c("Male","Female"), edu.cat = c(levels(MMSEdata$edu.cat)), diagn=c(levels(MMSEdata$diagn)),Supplement = c(0,1))

mm <- model.matrix(delete.response(terms(Supplement)),newdat.Supplement)
newdat.Supplement$manualEstimate = drop(mm %*% fixef(Supplement))
varMatrix <- mm %*% vcov(Supplement) %*% t(mm)
VarPointEstimates<-diag(varMatrix)

newdat.Supplement$manualSE = sqrt(VarPointEstimates)
newdat.Supplement$predictSE = predict(Supplement, newdata = newdat.Supplement,type = 'link',se.fit = TRUE)$se.fit
newdat.Supplement$MMSEestimate<-30-exp(newdat.Supplement$manualEstimate)
newdat.Supplement$UCI = 30-exp((newdat.Supplement$manualEstimate +1.96*newdat.Supplement$predictSE))
newdat.Supplement$LCI = 30-exp((newdat.Supplement$manualEstimate -1.96*newdat.Supplement$predictSE))

newdat.Supplement.only<-newdat.Supplement[newdat.Supplement$Supplement==1,]
newdat.Supplement.only$Supplement<-rep("Supplement",length(newdat.Supplement.only$Supplement))

##### Non-Supplement

newdat.No.Supplement<-newdat.Supplement[newdat.Supplement$Supplement==0,]
newdat.No.Supplement$Supplement<-rep("No-Supplement",length(newdat.Supplement.only$Supplement))

#### Combining the model matrix

combined<-rbind(newdat.multi.only,newdat.iron.only,newdat.No.Supplement,newdat.Supplement.only,newdat.magnesium.only,newdat.calcium.only,newdat.Selenium.only,newdat.zinc.only)
combined$Supplement<-as.factor(combined$Supplement)
levels(combined$Supplement)

## [1] "calcium" "iron" "magnesium" "multi"
## [5] "No-Supplement" "Selenium" "Supplement" "zinc"

write.csv(combined,"CombinedMMSEestimates.csv")

### Cleaning model matrices

newdat<-read.csv("CombinedMMSEestimates.csv",header=T)
str(newdat)

## 'data.frame': 9984 obs. of 15 variables:
## $ X : int 1249 1250 1251 1252 1253 1254 1255 1256 1257 1258 ...
## $ ID : int 120 120 120 120 120 120 120 120 120 120 ...
## $ M : int 0 6 12 18 24 36 48 60 72 84 ...
## $ AGE : int 0 0 0 0 0 0 0 0 0 0 ...
## $ APOE4 : int 0 0 0 0 0 0 0 0 0 0 ...
## $ Gender : Factor w/ 2 levels "Female","Male": 2 2 2 2 2 2 2 2 2 2 ...
## $ edu.cat : Factor w/ 4 levels "1post","2tertiary",..: 1 1 1 1 1 1 1 1 1 1 ...
## $ diagn : Factor w/ 4 levels "1CN","2EMCI",..: 1 1 1 1 1 1 1 1 1 1 ...
## $ manualEstimate: num -0.659 -0.595 -0.531 -0.466 -0.402 ...
## $ manualSE : num 0.0756 0.0768 0.0785 0.0807 0.0832 ...
## $ predictSE : num 0.0756 0.0768 0.0785 0.0807 0.0832 ...
## $ MMSEestimate : num 29.5 29.4 29.4 29.4 29.3 ...
## $ UCI : num 29.4 29.4 29.3 29.3 29.2 ...
## $ LCI : num 29.6 29.5 29.5 29.5 29.4 ...
## $ Supplement : Factor w/ 8 levels "calcium","iron",..: 4 4 4 4 4 4 4 4 4 4 ...

newdat$Supplement<-as.factor(newdat$Supplement)

levels(newdat$Supplement)

## [1] "calcium" "iron" "magnesium" "multi"
## [5] "No-Supplement" "Selenium" "Supplement" "zinc"

### Graphing LMCI with and without supplement use

This code graphs predicted cognitive decline of a MCI male individual with and without different supplements included in the model.

#### Zinc

red<-newdat[newdat$APOE4==0 & newdat$Gender=="Male" & newdat$edu.cat=="1post"& newdat$diagn=="3LMCI"& newdat$Supplement=="zinc",]
blue<-newdat[newdat$APOE4==0 & newdat$Gender=="Male" & newdat$edu.cat=="1post"& newdat$diagn=="3LMCI"& newdat$Supplement=="No-Supplement",]

plotdat<-rbind(red,blue)


Month<-c(0,6,12,18,24,36,48,60,72,84,96,108,120)
yaxismin<-max((floor(min(plotdat$MMSEestimate))-1),5)

xmax<-min(min(max(blue$M[which(blue$UCI>5, arr.ind=TRUE)]),120),min(max(red$M[which(red$UCI>5, arr.ind=TRUE)]),120))

#pdf("MMSEZinc.pdf", width=16/2.54, heigh=12/2.54 )
plot(Month,red$MMSEestimate,pch=NA,ylab="MMSE", xlab="Month",ylim=c(yaxismin, 30),xlim=c(-2,xmax),axes = FALSE)
axis(1,seq(0,xmax,by=12))
axis(2,at=seq(30,yaxismin,by=-3), cex.axis=0.8)
polygon(c(rev(Month), Month), c(rev(red$LCI), red$UCI), col = rgb(0.8,0,0,0.2), border = NA)
polygon(c(rev(Month), Month), c(rev(blue$LCI), blue$UCI), col = rgb(0,0,0.8,0.2), border = NA)
lines(Month,red$MMSEestimate,col="#db4c4c",lty=1,lwd=3)
lines(Month,blue$MMSEestimate,col="#66a3e0",lty=1,lwd=3)
legend("bottomleft", bty = "n", legend = as.character(c("Non-User", "Zinc User")),
 pch = 15, col = c("#7cafe2","#f78a8a"), cex = 1, horiz=F)


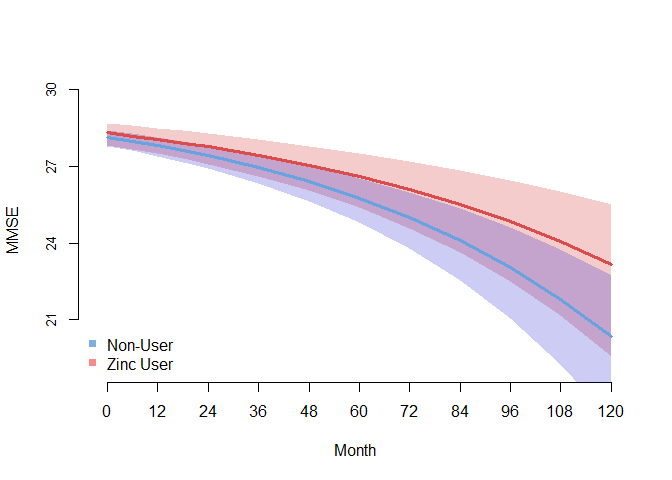


This figure depicts the predicted cognitive decline of a male MCI individual with and without the zinc supplement variable included in the model.

#dev.off()

#### Magnesium

red<-newdat[newdat$APOE4==0 & newdat$Gender=="Male" & newdat$edu.cat=="1post"& newdat$diagn=="3LMCI"& newdat$Supplement=="magnesium",]
blue<-newdat[newdat$APOE4==0 & newdat$Gender=="Male" & newdat$edu.cat=="1post"& newdat$diagn=="3LMCI"& newdat$Supplement=="No-Supplement",]

plotdat<-rbind(red,blue)


Month<-c(0,6,12,18,24,36,48,60,72,84,96,108,120)
yaxismin<-max((floor(min(plotdat$MMSEestimate))-1),5)

xmax<-min(min(max(blue$M[which(blue$UCI>5, arr.ind=TRUE)]),120),min(max(red$M[which(red$UCI>5, arr.ind=TRUE)]),120))

#pdf("MMSEMagnesium.pdf", width=16/2.54, heigh=12/2.54 )
plot(Month,red$MMSEestimate,pch=NA,ylab="MMSE", xlab="Month",ylim=c(yaxismin, 30),xlim=c(-2,xmax),axes = FALSE)
axis(1,seq(0,xmax,by=12))
axis(2,at=seq(30,yaxismin,by=-3), cex.axis=0.8)
polygon(c(rev(Month), Month), c(rev(red$LCI), red$UCI), col = rgb(0.8,0,0,0.2), border = NA)
polygon(c(rev(Month), Month), c(rev(blue$LCI), blue$UCI), col = rgb(0,0,0.8,0.2), border = NA)
lines(Month,red$MMSEestimate,col="#db4c4c",lty=1,lwd=3)
lines(Month,blue$MMSEestimate,col="#66a3e0",lty=1,lwd=3)
legend("bottomleft", bty = "n", legend = as.character(c("Non-User", "Magnesium User")),
 pch = 15, col = c("#7cafe2","#f78a8a"), cex = 1, horiz=F)


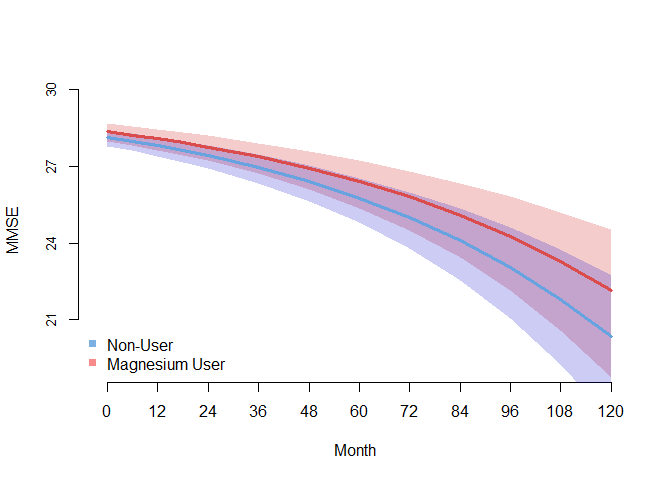


This figure depicts the predicted cognitive decline of a male MCI individual with and without the magnesium supplement variable included in the model.

#dev.off()

#### Calcium

red<-newdat[newdat$APOE4==0 & newdat$Gender=="Male" & newdat$edu.cat=="1post"& newdat$diagn=="3LMCI"& newdat$Supplement=="calcium",]
blue<-newdat[newdat$APOE4==0 & newdat$Gender=="Male" & newdat$edu.cat=="1post"& newdat$diagn=="3LMCI"& newdat$Supplement=="No-Supplement",]

plotdat<-rbind(red,blue)


Month<-c(0,6,12,18,24,36,48,60,72,84,96,108,120)
yaxismin<-max((floor(min(plotdat$MMSEestimate))-1),5)

xmax<-min(min(max(blue$M[which(blue$UCI>5, arr.ind=TRUE)]),120),min(max(red$M[which(red$UCI>5, arr.ind=TRUE)]),120))

#pdf("MMSEcalcium.pdf", width=16/2.54, heigh=12/2.54 )
plot(Month,red$MMSEestimate,pch=NA,ylab="MMSE", xlab="Month",ylim=c(yaxismin, 30),xlim=c(-2,xmax),axes = FALSE)
axis(1,seq(0,xmax,by=12))
axis(2,at=seq(30,yaxismin,by=-3), cex.axis=0.8)
polygon(c(rev(Month), Month), c(rev(red$LCI), red$UCI), col = rgb(0.8,0,0,0.2), border = NA)
polygon(c(rev(Month), Month), c(rev(blue$LCI), blue$UCI), col = rgb(0,0,0.8,0.2), border = NA)
lines(Month,red$MMSEestimate,col="#db4c4c",lty=1,lwd=3)
lines(Month,blue$MMSEestimate,col="#66a3e0",lty=1,lwd=3)
legend("bottomleft", bty = "n", legend = as.character(c("Non-User", "Calcium User")),
 pch = 15, col = c("#7cafe2","#f78a8a"), cex = 1, horiz=F)


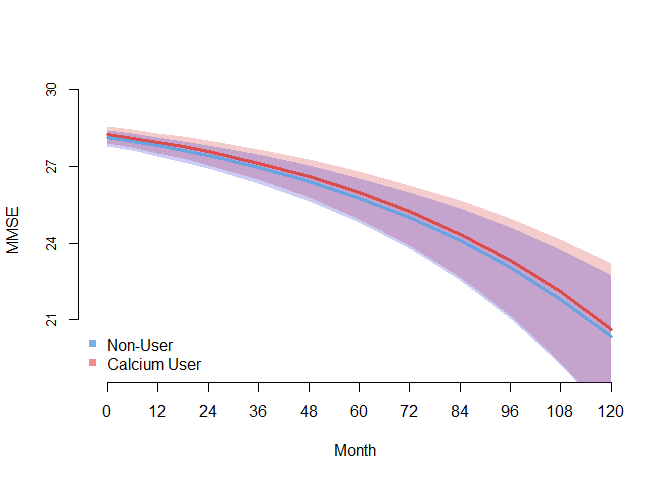
 This figure depicts the predicted cognitive decline of a male MCI individual with and without the calcium supplement variable included in the model.

#dev.off()

#### Selenium

red<-newdat[newdat$APOE4==0 & newdat$Gender=="Male" & newdat$edu.cat=="1post"& newdat$diagn=="3LMCI"& newdat$Supplement=="Selenium",]
blue<-newdat[newdat$APOE4==0 & newdat$Gender=="Male" & newdat$edu.cat=="1post"& newdat$diagn=="3LMCI"& newdat$Supplement=="No-Supplement",]

plotdat<-rbind(red,blue)


Month<-c(0,6,12,18,24,36,48,60,72,84,96,108,120)
yaxismin<-max((floor(min(plotdat$MMSEestimate))-1),5)

xmax<-min(min(max(blue$M[which(blue$UCI>5, arr.ind=TRUE)]),120),min(max(red$M[which(red$UCI>5, arr.ind=TRUE)]),120))

#pdf("MMSEselenium.pdf", width=16/2.54, heigh=12/2.54 )
plot(Month,red$MMSEestimate,pch=NA,ylab="MMSE", xlab="Month",ylim=c(yaxismin, 30),xlim=c(-2,xmax),axes = FALSE)
axis(1,seq(0,xmax,by=12))
axis(2,at=seq(30,yaxismin,by=-3), cex.axis=0.8)
polygon(c(rev(Month), Month), c(rev(red$LCI), red$UCI), col = rgb(0.8,0,0,0.2), border = NA)
polygon(c(rev(Month), Month), c(rev(blue$LCI), blue$UCI), col = rgb(0,0,0.8,0.2), border = NA)
lines(Month,red$MMSEestimate,col="#db4c4c",lty=1,lwd=3)
lines(Month,blue$MMSEestimate,col="#66a3e0",lty=1,lwd=3)
legend("bottomleft", bty = "n", legend = as.character(c("Non-User", "Selenium User")),
 pch = 15, col = c("#7cafe2","#f78a8a"), cex = 1, horiz=F)


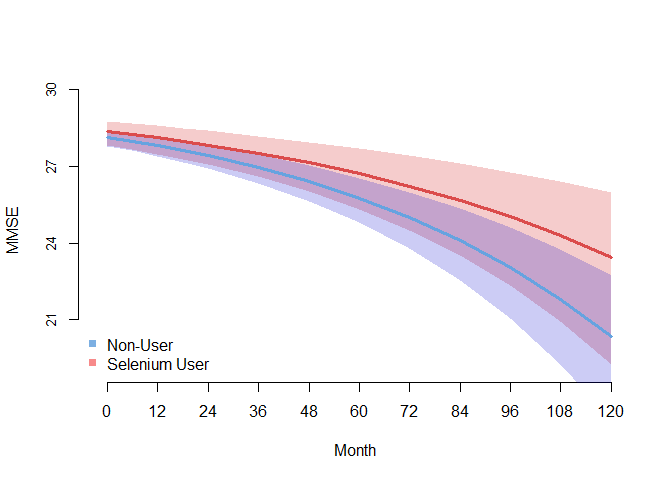


This figure depicts the predicted cognitive decline of a male MCI individual with and without the selenium supplement variable included in the model.

#dev.off()

#### Iron

red<-newdat[newdat$APOE4==0 & newdat$Gender=="Male" & newdat$edu.cat=="1post"& newdat$diagn=="3LMCI"& newdat$Supplement=="iron",]
blue<-newdat[newdat$APOE4==0 & newdat$Gender=="Male" & newdat$edu.cat=="1post"& newdat$diagn=="3LMCI"& newdat$Supplement=="No-Supplement",]

plotdat<-rbind(red,blue)


Month<-c(0,6,12,18,24,36,48,60,72,84,96,108,120)
yaxismin<-max((floor(min(plotdat$MMSEestimate))-1),5)

xmax<-min(min(max(blue$M[which(blue$UCI>5, arr.ind=TRUE)]),120),min(max(red$M[which(red$UCI>5, arr.ind=TRUE)]),120))

#pdf("MMSEiron.pdf", width=16/2.54, heigh=12/2.54 )
plot(Month,red$MMSEestimate,pch=NA,ylab="MMSE", xlab="Month",ylim=c(yaxismin, 30),xlim=c(-2,xmax),axes = FALSE)
axis(1,seq(0,xmax,by=12))
axis(2,at=seq(30,yaxismin,by=-3), cex.axis=0.8)
polygon(c(rev(Month), Month), c(rev(red$LCI), red$UCI), col = rgb(0.8,0,0,0.2), border = NA)
polygon(c(rev(Month), Month), c(rev(blue$LCI), blue$UCI), col = rgb(0,0,0.8,0.2), border = NA)
lines(Month,red$MMSEestimate,col="#db4c4c",lty=1,lwd=3)
lines(Month,blue$MMSEestimate,col="#66a3e0",lty=1,lwd=3)
legend("bottomleft", bty = "n", legend = as.character(c("Non-User", "iron User")),
 pch = 15, col = c("#7cafe2","#f78a8a"), cex = 1, horiz=F)


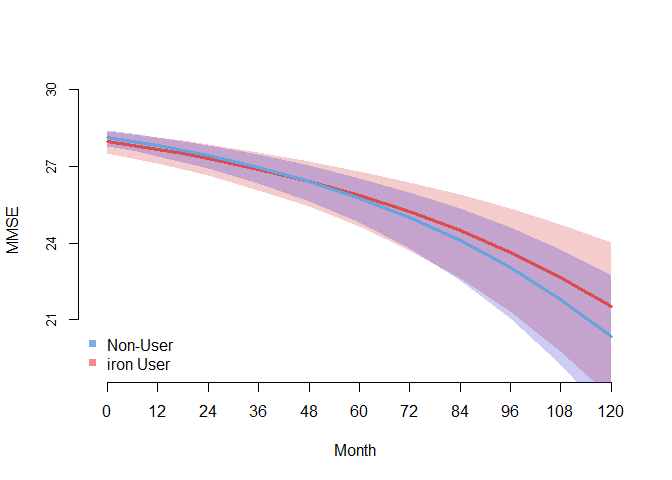


This figure depicts the predicted cognitive decline of a male MCI individual with and without the iron supplement variable included in the model.

#dev.off()

#### Supplement

red<-newdat[newdat$APOE4==0 & newdat$Gender=="Male" & newdat$edu.cat=="1post"& newdat$diagn=="3LMCI"& newdat$Supplement=="Supplement",]
blue<-newdat[newdat$APOE4==0 & newdat$Gender=="Male" & newdat$edu.cat=="1post"& newdat$diagn=="3LMCI"& newdat$Supplement=="No-Supplement",]

plotdat<-rbind(red,blue)


Month<-c(0,6,12,18,24,36,48,60,72,84,96,108,120)
yaxismin<-max((floor(min(plotdat$MMSEestimate))-1),5)

xmax<-min(min(max(blue$M[which(blue$UCI>5, arr.ind=TRUE)]),120),min(max(red$M[which(red$UCI>5, arr.ind=TRUE)]),120))

#pdf("MMSESupplement.pdf", width=16/2.54, heigh=12/2.54 )
plot(Month,red$MMSEestimate,pch=NA,ylab="MMSE", xlab="Month",ylim=c(yaxismin, 30),xlim=c(-2,xmax),axes = FALSE)
axis(1,seq(0,xmax,by=12))
axis(2,at=seq(30,yaxismin,by=-3), cex.axis=0.8)
polygon(c(rev(Month), Month), c(rev(red$LCI), red$UCI), col = rgb(0.8,0,0,0.2), border = NA)
polygon(c(rev(Month), Month), c(rev(blue$LCI), blue$UCI), col = rgb(0,0,0.8,0.2), border = NA)
lines(Month,red$MMSEestimate,col="#db4c4c",lty=1,lwd=3)
lines(Month,blue$MMSEestimate,col="#66a3e0",lty=1,lwd=3)
legend("bottomleft", bty = "n", legend = as.character(c("Non-User", "Supplement User")),
 pch = 15, col = c("#7cafe2","#f78a8a"), cex = 1, horiz=F)


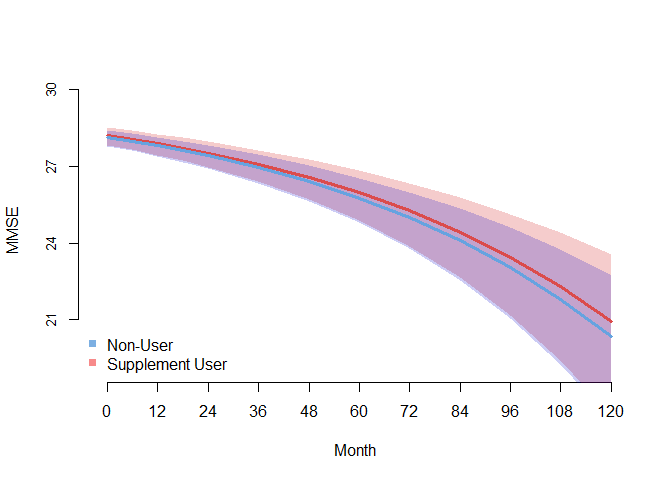


This figure depicts the predicted cognitive decline of a male MCI individual with and without the any supplment variable included in the model.

#dev.off()

#### Multi-vitamin

red<-newdat[newdat$APOE4==0 & newdat$Gender=="Male" & newdat$edu.cat=="1post"& newdat$diagn=="3LMCI"& newdat$Supplement=="multi",]
blue<-newdat[newdat$APOE4==0 & newdat$Gender=="Male" & newdat$edu.cat=="1post"& newdat$diagn=="3LMCI"& newdat$Supplement=="No-Supplement",]

plotdat<-rbind(red,blue)


Month<-c(0,6,12,18,24,36,48,60,72,84,96,108,120)
yaxismin<-max((floor(min(plotdat$MMSEestimate))-1),5)

xmax<-min(min(max(blue$M[which(blue$UCI>5, arr.ind=TRUE)]),120),min(max(red$M[which(red$UCI>5, arr.ind=TRUE)]),120))

#pdf("MMSEMutli.pdf", width=16/2.54, heigh=12/2.54 )
plot(Month,red$MMSEestimate,pch=NA,ylab="MMSE", xlab="Month",ylim=c(yaxismin, 30),xlim=c(-2,xmax),axes = FALSE)
axis(1,seq(0,xmax,by=12))
axis(2,at=seq(30,yaxismin,by=-3), cex.axis=0.8)
polygon(c(rev(Month), Month), c(rev(red$LCI), red$UCI), col = rgb(0.8,0,0,0.2), border = NA)
polygon(c(rev(Month), Month), c(rev(blue$LCI), blue$UCI), col = rgb(0,0,0.8,0.2), border = NA)
lines(Month,red$MMSEestimate,col="#db4c4c",lty=1,lwd=3)
lines(Month,blue$MMSEestimate,col="#66a3e0",lty=1,lwd=3)
legend("bottomleft", bty = "n", legend = as.character(c("Non-User", "Multi-vitamin User")),
 pch = 15, col = c("#7cafe2","#f78a8a"), cex = 1, horiz=F)


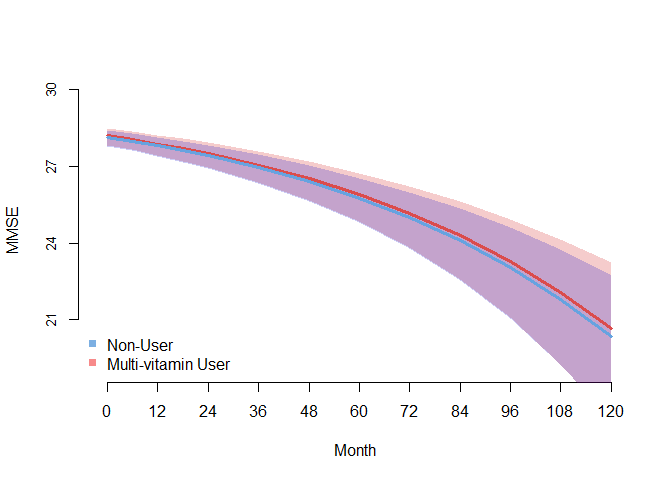


This figure depicts the predicted cognitive decline of a male MCI individual with and without the multivitamin supplement variable included in the model.

#dev.off()
